# Persistent Room Temperature Phosphorescence from Triarylboranes: A Combined Experimental and Theoretical Study

**DOI:** 10.1002/anie.202007610

**Published:** 2020-08-04

**Authors:** Zhu Wu, Jörn Nitsch, Julia Schuster, Alexandra Friedrich, Katharina Edkins, Marcel Loebnitz, Fabian Dinkelbach, Vladimir Stepanenko, Frank Würthner, Christel M. Marian, Lei Ji, Todd B. Marder

**Affiliations:** ^1^ Institut für Anorganische Chemie and Institute for Sustainable Chemistry & Catalysis with Boron Julius-Maximilians-Universität Würzburg Am Hubland 97074 Würzburg Germany; ^2^ School of Health Sciences The University of Manchester Oxford Road Manchester M13 9PL UK; ^3^ Institut für Theoretische Chemie und Computerchemie Heinrich-Heine-Universität Düsseldorf Universitätsstr. 1 40225 Düsseldorf Germany; ^4^ Institut für Organische Chemie Julius-Maximilians-Universität Würzburg Am Hubland 97074 Würzburg Germany; ^5^ Frontiers Science Center for Flexible Electronics (FSCFE) & Shaanxi Institute of Flexible Electronics (SIFE) Northwestern Polytechnical University 127 West Youyi Road 710072 Xi'an China

**Keywords:** AIE, boron, El-Sayed's rule, non-radiative transition, RTP

## Abstract

Achieving highly efficient phosphorescence in purely organic luminophors at room temperature remains a major challenge due to slow intersystem crossing (ISC) rates in combination with effective non‐radiative processes in those systems. Most room temperature phosphorescent (RTP) organic materials have O‐ or N‐lone pairs leading to low lying (n, π*) and (π, π*) excited states which accelerate *k*_isc_ through El‐Sayed's rule. Herein, we report the first persistent RTP with lifetimes up to 0.5 s from simple triarylboranes which have no lone pairs. RTP is only observed in the crystalline state and in highly doped PMMA films which are indicative of aggregation induced emission (AIE). Detailed crystal structure analysis suggested that intermolecular interactions are important for efficient RTP. Furthermore, photophysical studies of the isolated molecules in a frozen glass, in combination with DFT/MRCI calculations, show that (σ, B p)→(π, B p) transitions accelerate the ISC process. This work provides a new approach for the design of RTP materials without (n, π*) transitions.

## Graphical abstract

Efficient intersystem crossing (ISC) in combination with strong intermolecular interactions suppresses the nonradiative decay rate *k*_nr_ and leads to the first reported persistent room temperature phosphorescence (RTP) from purely organic triarylborane phosphors, which do not possess O‐ or N‐lone pairs. Transitions between local σ‐ and π‐excitation also accelerate *k*_isc_.

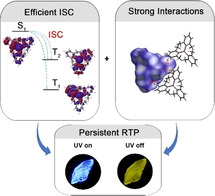

## Introduction

**Figure 1:**
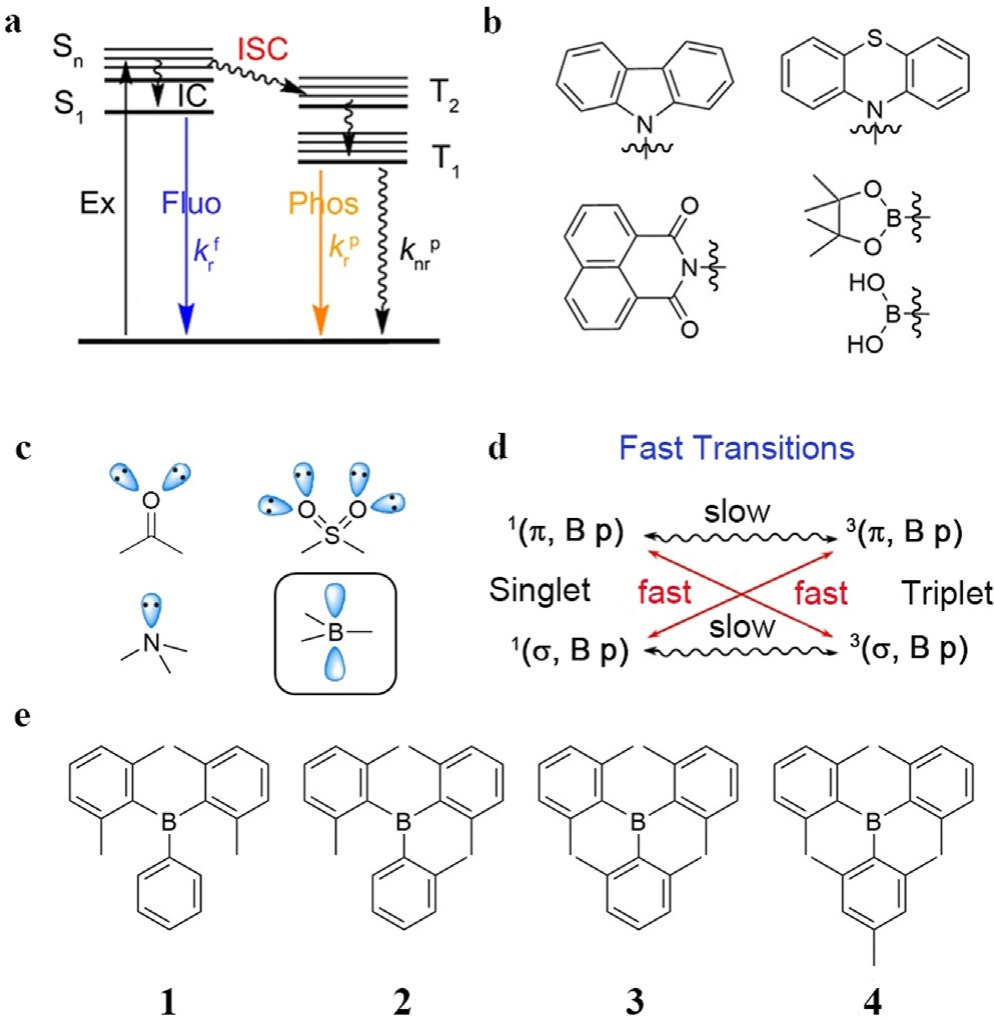
a) Jablonski‐diagram. b) The structural features of reported RTP materials. c) Typical functional groups having lone pairs in organic phosphors, and the empty p_z_ orbital on three‐coordinate boron. d) Fast transitions between (σ, B p) and (π, B p). e) Molecular structures of compounds **1**–**4**.

Luminophores with ultralong room temperature phosphorescence (RTP) have attracted much attention because of a variety of applications in time‐gated biological imaging,^1^ anti‐counterfeiting,^2^ watch dials, safety signs, and optoelectronic devices.^3^ Unlike metal‐containing materials, in which the heavy atom effect can efficiently accelerate the intersystem crossing (ISC) process from singlet to triplet excited states,^4^ RTP from purely organic molecules is relatively rare because the formation of the triplet states is usually not efficient as ISC is slow. In addition, radiative decay from T_1_ to the S_0_ ground state is also spin forbidden, and is very slow compared to the non‐radiative relaxation from T_1_ in an unrestricted environment.^5^ Designing purely organic systems showing ultralong RTP is a challenge.^6^ Key approaches involve reducing the nonradiative decay rate (*k*_nr_(T_1_)) from T_1_ by avoiding collisions with quenching species such as oxygen, and minimizing vibrational relaxation (Figure 1 a).^7^ For example, Tang and co‐workers reported purely organic luminophores which phosphoresce in the crystalline state.^8^ Huang and colleagues proposed that effective stabilization of triplet excited states through strong coupling in H‐aggregated molecules enables their lifetimes to become orders of magnitude longer than those of conventional organic fluorophores.^9^ Adachi and co‐workers developed efficient persistent RTP materials by minimizing nonradiative decay rates in organic amorphous host–guest materials.^10^ Very recently, Wang and co‐workers have achieved ultralong RTP from *N*‐phenyl‐2‐naphthylamine by confining it in a crystalline dibromobiphenyl matrix.^11^ To increase the population of triplet excitons, heteroatoms with lone pairs are usually introduced into organic systems to enhance spin–orbit coupling (El‐Sayed's rule),^12^ which is why most RTP phosphors are limited to phenothiazine, carbazole, and naphthylimide derivatives (Figure 1 b).1b, 4d, 13 Thus, intersystem crossing usually involves ^1^(n, π*)→^3^(π, π*) transitions. Recently, arylboronic acids and esters, which also contain lone pairs on their hydroxy or alkoxy groups, have been reported to show RTP with lifetimes up to several seconds in the solid state.^14^ Thus far, ultralong RTP from purely organic phosphors without lone pairs has rarely been reported,^15^ as *k*_isc_ is slow.

In fact, organic compounds without lone pairs,^16^ such as triarylboranes, can show phosphorescence in a frozen optical glass at 77 K (Figure 1 c).^17^ This indicates that *k*_isc_ in a photo‐excited triarylborane molecule can compete with fluorescence, for which the rate constant is usually on the order of 10^7^ s^−1^. Therefore, we propose that *k*_isc_ can also be accelerated by (σ, B p)→(π, B p) transitions, which would be the inversion of the normally observed ^1^(n, π*)→^3^(π, π*) ISC process (Figure 1 d). However, probably due to the fact that the non‐radiative decay rate from T_1_ *k*_nr_^p^ at RT is usually much faster than the phosphorescence, RTP from triarylboranes has not been reported. Only if *k*_nr_^p^ is suppressed to a large extent, might we observe RTP from triarylboranes. In 1955, Wittig et al. reported that some triarylboranes, including tris(2‐methylphenyl)borane, showed a yellowish‐white emission under UV light.^18^ However, no lifetimes were reported and, when we prepared tris(2‐methylphenyl)borane, it showed only blue fluorescence; in other words, no phosphorescence at room temperature was detected (Supporting Information, Figures S14 and S15). Given our interest in the linear and nonlinear optical properties of 3‐coordinate organoboron compounds,^19^ we examined the triarylboranes **1**–**4** (Figure 1 e). Crystalline samples of **3** (tris(2,6‐dimethylphenyl)borane) show ultralong (*τ*_p_=478 ms), intense, yellow phosphorescence under ambient conditions, and it is thus, to the best of our knowledge, the first triarylboron compound without lone pairs to display ultralong RTP.

## Results and Discussion

**Figure 2:**
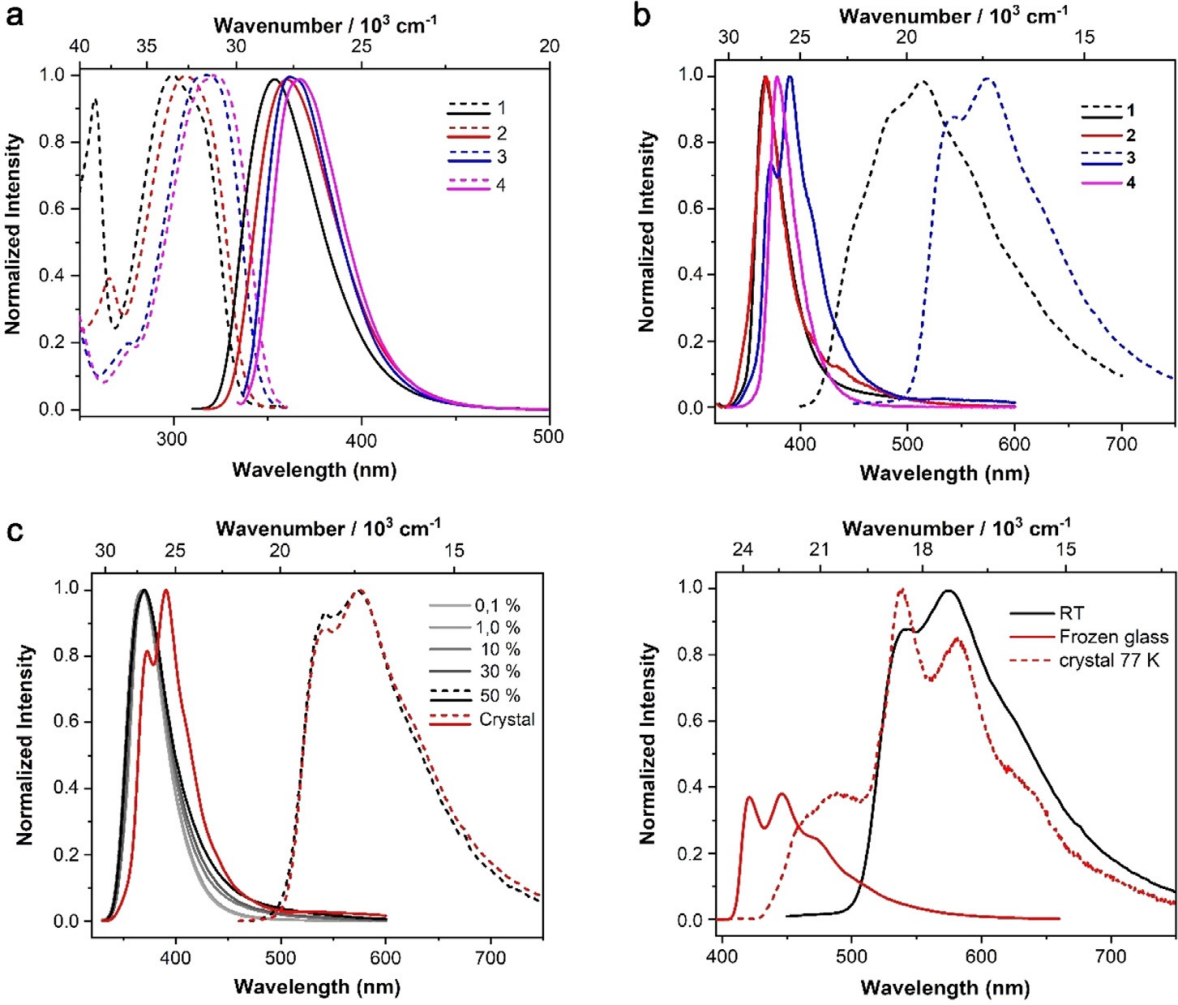
a) Normalized UV/Vis absorption (dashed lines) and fluorescence emission (solid lines) spectra of **1**–**4** in hexane solution at room temperature (*λ*_exc_=290 nm). b) Photoluminescence (PL) emission spectra (solid lines) of crystalline **1**–**4**, and time‐gated phosphorescence emission (dashed lines) spectra at room temperature (*λ*_exc_=305 nm). c) Total PL emission (solid lines) and time‐gated phosphorescence emission (dashed lines) spectra of **3** at 0.1, 1.0, 10, 30, and 50 % loadings in PMMA films and in the crystalline state at room temperature (*λ*_exc_=305 nm). d) Time‐gated phosphorescence emission spectra of compound **3** in the crystalline state at room temperature (solid black), frozen methylcylohexane glass matrix (solid red), and crystalline state (dashed line) at 77 K.

The synthesis and characterization of all compounds are given in the Supporting Information and the photophysical properties of **1**–**4** are summarized in Table 1. The important results of our quantum chemical studies are shown in brackets, and complete data are given in Tables S2 and S4 in the Supporting Information. The UV/Vis absorption and emission spectra were first measured in hexane. Compounds **1**–**4** all show a broad first absorption band between 280–350 nm in hexane, which can be assigned to B←π transitions, that is, a transition from the aryl ring π‐systems to the empty *p*‐orbital on the boron atom (Figure 2 a). Our calculations reveal that this band is formed by up to five electronic transitions, S_1_←S_0_ to S_4_←S_0_ in the *D*_3_‐symmetric compound **3** and S_1_←S_0_ to S_5_←S_0_ in the less symmetric compounds **1**, **2**, and **4** (Supporting Information, Figures S1–S5). The energies of the absorption maxima decrease in the order **1**>**2**>**3**>**4**. This indicates that introducing each methyl substituent, a weak σ‐donor, on the phenyl ring, redshifts the absorption spectra by 6–12 nm (580–1230 cm^−1^). The fluorescence spectra of the compounds in hexane show the same trend; their maxima redshift 2–7 nm (150–530 cm^−1^) for each methyl group added to the phenyl ring (Figure 2 a). However, the emission spectra of crystalline **1**–**4** are not related to their chemical structures in an obvious way (Figure 2 b). In general, the fluorescence spectra of the solid, crystalline samples of compounds **1**–**4** are all redshifted compared with those in hexane solution. The bathochromic shift of **3** (2060 cm^−1^) is considerably larger than those of **1**, **2**, and **4**. The bathochromic shifts of **2** (750 cm^−1^) and **4** (930 cm^−1^) are smaller than that of **1** (1150 cm^−1^). This indicates that intermolecular interactions in crystalline **1** and **3** are larger than those in **2** and **4**, which is one possible explanation for the slower nonradiative decay (*k*_nr_) from both S_1_ and T_1_, see below. In addition, compounds **1** and **3** may also have a higher probability of showing excimer emissions.

**Table 1:** Experimental and calculated (in brackets) photophysical properties of compounds **1**–**4** in hexane and the crystalline state at RT, and in a frozen methylcyclohexane glass at 77 K.

	State	*λ*_f_ [nm]	*Φ*_f_ [%]	*k*_r_^f^ [×10^7^ s^−1^]	*k*_nr_^f^ [×10^8^ s^−1^]	*k*_isc_ [s^−1^]	*λ*_p_ [nm]	*Φ*_p_ [%]	*τ*_p_ [s]
**1**	Crystalline^[a]^	369	3.4	2	6.0		524	0.3	0.09 (21 %), 0.68 (79 %)
	Crystalline^[b]^	368					471, 502, 541		2.27
	Frozen glass^[b]^	349 {383}				{1×10^7^}	404, 427 {425, 597}		1.45 {8}
									
**2**	Crystalline^[a]^	369	6.9	4	5.8		nd^[c]^		nd
	Crystalline^[b]^	352, 366					426, 449		0.22 (39 %), 1.22 (61 %)
	Frozen glass^[b]^	373 {391}				{6×10^6^}	417, 442 {448, 476}		1.57 {8}
									
**3**	Crystalline^[a]^	371, 390	17.0	10	5.9		540, 575	1.2	0.48
	Crystalline^[b]^	372, 392, 415					488, 538, 582, 630		0.52 (23 %), 1.64 (77 %)
	Frozen glass^[b]^	375 {404}				{5×10^7^}	421, 446 {456, 486}		1.48 {7}
									
**4**	Crystalline^[a]^	381	6.3	4	6.2		nd^[c]^		nd
	Crystalline^[b]^	370					456, 485		0.08 (32 %), 1.32 (68 %)
	Frozen glass^[b]^	374 {430}				{3×10^7^}	425, 452 {458, 489}		1.36 {5}

[a] Measured at RT. [b] Measured at 77 K. [c] Not detected (nd).

**Figure 3:**
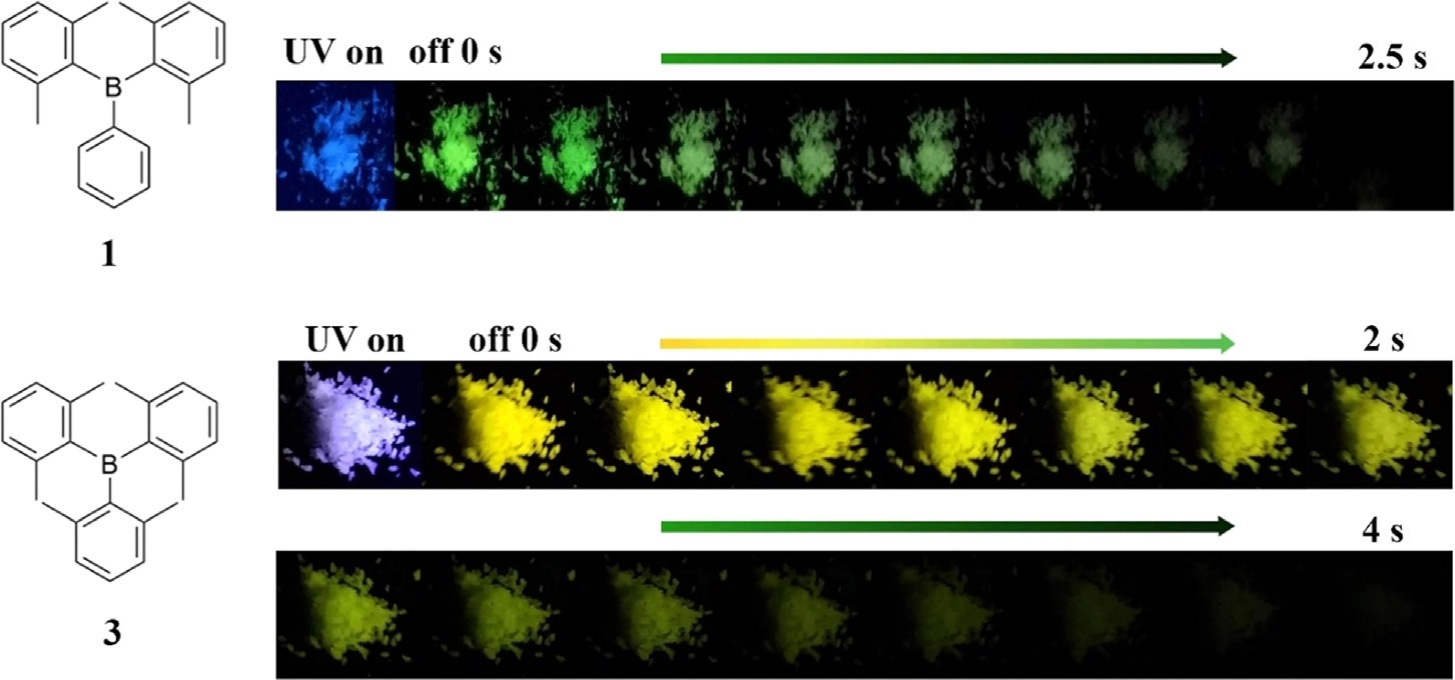
Photographs of crystalline **1** and **3** taken during and after irradiation (365 nm) under ambient conditions.

We noticed that upon exposure to a hand‐held UV‐lamp (*λ*=365 nm), crystalline **3** showed violet fluorescence which disappears immediately when the lamp is turned off. Persistent greenish‐yellow phosphorescence emission was then observed, which is visible to the naked eye for almost 4 s (Figure 3). Time‐gated emission spectroscopy revealed long‐lived (*τ*=478 ms) phosphorescence from crystalline **3**, with an emission maximum at 575 nm and a shoulder at 540 nm (Figures 2 b and S13 in the Supporting Information). To the best of our knowledge, this is the first triarylborane without any heavy atom6j to show long‐lived RTP, and one of the rare examples where free electron pairs are absent. In addition to the RTP from **3**, RTP was also observed from crystalline **1**, with a phosphorescence emission maximum at 515 nm and a lifetime of *τ*=680 ms. Compared to compound **3**, the phosphorescence quantum yield (*Φ*_P_) of **1** is 0.26 %, which is lower than that of **3** (1.14 %). We did not observe any phosphorescence from compounds **2** and **4** at room temperature.

Interestingly, we found that the photoluminescence behavior of **3** largely depends on its aggregation state. We investigated two different kinds of aggregation states, crystalline sample **A** and ball‐milled sample **B**. SEM pictures and powder X‐ray diffraction (pXRD) patterns clearly revealed the difference between the samples. In the SEM pictures of the ball‐milled powder, we can see smaller size particles with a larger surface area (Supporting Information, Figure S21). This is in agreement with the powder X‐ray diffraction pattern of the ball‐milled sample, which shows broader reflections compared to the diffraction pattern obtained from the crystalline sample **A** (Supporting Information, Figure S29), and this indicates that sample **B** contains much smaller crystallites than the sample **A**. We find that the emission maxima differ by 25 nm (1760 cm^−1^) and that the peak at 350 nm in the excitation spectrum of sample **A** decreases in intensity as the crystalline domains increase (Supporting Information, Figures S23 and S24). Although the fluorescence lifetime and the time‐gated phosphorescence emission spectra remained the same, the phosphorescence lifetime and quantum yield decreased significantly for the ball‐milled sample **B** compared with crystalline **A**, from 478 to 340 ms and 1.12 % to 0.2 %, respectively. In the ball‐milled powder, the exposed surface area is much larger, and phosphorescence is more sensitive to oxygen quenching, compared to the crystalline state. This hypothesis is supported by phosphorescence lifetime measurements under argon, for which the difference between the two samples disappears (Supporting Information, Figures S27 and S28).

**Figure 4:**
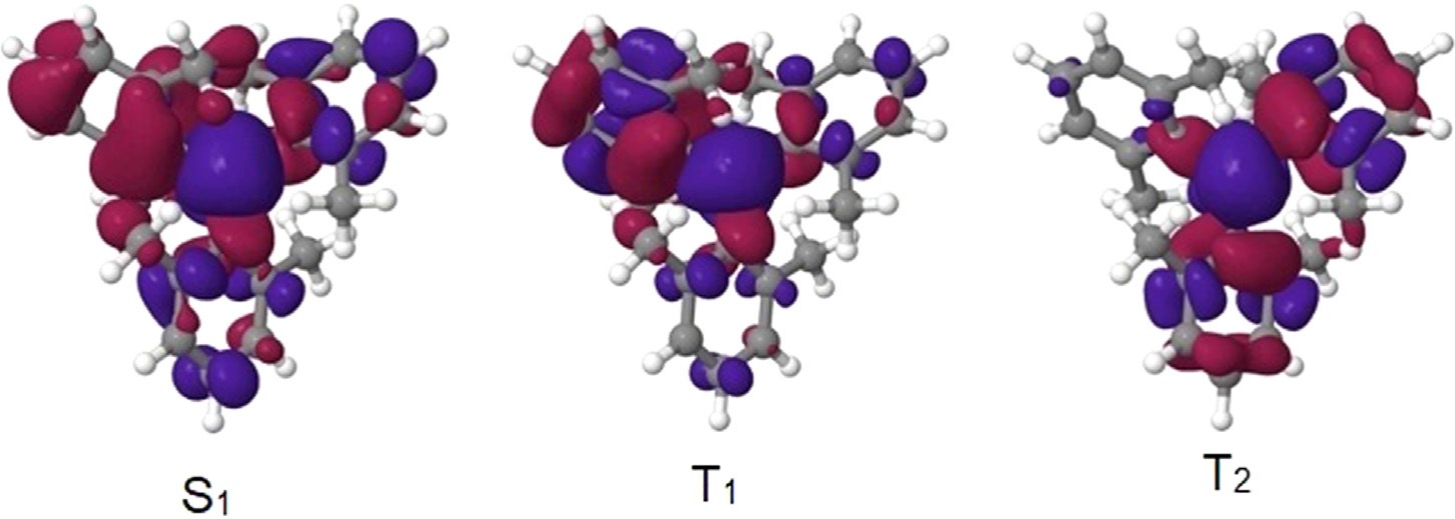
Difference densities (|isovalue|=0.001 [*e* Å^−3^]^1/2^) of low‐lying excited states of compound **3** at the TD‐DFT‐optimized geometry of the S_1_ state. The loss of electron density with respect to the S_0_ state is indicated in red and the gain in blue.

To understand further the relationship between molecular structure and phosphorescence, we measured the emission spectra of **1**–**4** in a frozen methylcyclohexane optical glass at 77 K (Supporting Information, Figures S30 and S31), where we can assume that there are no intermolecular interactions present (*c*<10^−5^ mol L^−1^). All four compounds show two well‐separated emission bands. We observed phosphorescence emissions (400–600 nm), which are all hypsochromically shifted in comparison to the emission from the solid at room temperature (by 5230–5670 cm^−1^). All compounds show similar vibrational fine structures except compound **1**, because **1** has low‐frequency vibrational modes according to our calculations, which broaden the emission bands. In addition, there are high energy fluorescence emission bands (330–400 nm), which show less vibrational fine structure (Supporting Information, Figure S30). The maxima of the computed emission spectra (Supporting Information, Figures S8 and S11) are redshifted (by 1220–1820 cm^−1^) with respect to the experimental spectra in a frozen glass while the energies of the 0‐0 transitions agree well. The redshifts of the maxima are partially attributed to the harmonic oscillator approximation, which overestimates the intensities at the long wavelength tail of the emission spectrum that stems from electric dipole transitions between the vibrational ground state of the electronically excited state and vibrationally excited levels of the electronic ground state. The calculated values for *k*_isc_ of **1**–**4** are circa 10^7^ s^−1^, thus ISC can compete with fluorescence. Noticeably, the major components of the phosphorescence lifetimes of all four compounds are similar, with a value of circa 1.5 s (Supporting Information, Figure S32). Up to six triplet states are located energetically below or very close to the S_1_ state as shown in Figures S6 and S7 in the Supporting Information. Some of the triplet potential energy surfaces cross the S_1_ energy profile along the linear interpolated path connecting the Franck–Condon point with the minimum of the S_1_ state. ISC is nevertheless fastest for a transition between S_1_ and T_2_ in **1**, **2**, **3**, and **4**. To understand the origin of the non‐negligible spin–orbit coupling (SOC) between these states, we computed and plotted the differences of the electron densities between the ground and excited‐state wave functions. S_1_ and T_1_ of compound **3**, for example (Figure 4), result from similar (π, B p) excitations, with T_1_ showing additional contributions from local (π, π*) excitations on xylyl ring a. For this transition,^12^ the SOC is very small. Comparing the difference densities of S_1_ and T_2_ instead, we see two major differences. First, in T_2_, most of the electron density has been transferred from the other two xylyl rings b and c. As the largest SOCs result from one‐center terms, excitations from different π systems to the same boron orbital yield negligible interaction matrix elements. The second, and more important, difference with regard to SOC is a contribution to the T_2_ wave function in which charge is transferred from a σ‐type orbital connecting xylyl ring a with boron. The change of orbital angular momentum on this carbon atom leads to stronger SOC than expected in the absence of (n, π*) excitations. This evidence clearly demonstrates that *k*_isc_ can also be accelerated by (σ, B p)→(π, B p) and (π, B p)→(σ, B p) transitions. An electronic matrix element |<T_2_|H_SO_|S_1_>|≈1 cm^−1^ is sufficient for ISC to proceed at a rate of circa 10^7^ s^−1^ (for more details, see Tables S1 and S2 in the Supporting Information). As the calculated fluorescence and ISC rate constants are of the same order of magnitude, the competition between the two processes is easily explained. T_2_ and T_1_ form a Jahn–Teller pair which is degenerate in D_3_‐symmetric geometries. The states are thus coupled by strong vibronic interactions that facilitate fast T_2_ → T_1_ internal conversion. Although triplet formation is likely to occur in all compounds, no phosphorescence was detected in solution at room temperature, likely due to rapid nonradiative decay *k*_nr_(T_1_) compared to slow *k*_p_. Our data indicate that triplet excited states are formed in all compounds **1**–**4** after excitation.

We further noticed that the RTP emission in crystalline samples of **3** is noticeably redshifted by 5230 cm^−1^ when compared to that in the frozen glass (Figure 2 d). Such a large shift makes it unlikely that it results from the suppression of the internal conversion (temperature effect) in the excited state, or by a less polar environment (environment effect) in the frozen glass. To examine how temperature influences the luminescent behavior of crystalline samples, we also measured the emission spectra of crystalline **1**–**4** at 77 K (Supporting Information, Figure S33). In crystalline **3**, a sharp fluorescence peak appears at 415 nm at 77 K, which is almost identical to the fluorescence in the frozen glass. However, a very broad phosphorescent emission ranging from 430 to 720 nm (Figure 2 d) is observed, which we assign to two phosphorescence bands, one at 488 nm and a second ranging from 500 to 720 nm. We noticed that the band at 488 nm is only visible at low temperature and is most likely not a vibrational band of the 500–720 nm emission, for which the range is identical to the spectrum at room temperature (Figure 2 d). We observe two lifetimes, one of 1.64 s, and a second of 0.52 s, which further support the existence of two independent triplet states. We note that the longer lifetime is almost identical to the lifetime in the frozen glass, in which we can assume the absence of any intermolecular interaction except with solvent matrix molecules. We assume that the band at 488 nm is phosphorescence which is caused by the population of the T_1_ state of the triarylboranes and which is only visible when the non‐radiative decay is suppressed. Therefore, it cannot be observed at higher temperatures, at which *k*_nr_(T_1_) dominate. This emission is also found in the frozen glass in which it is shifted by 67 nm (3260 cm^−1^), which is a reasonable shift if one considers the different environments of the frozen glass matrix and the crystalline sample. The emission between 500–720 nm, however, is the real RTP emission which is an aggregation induced phenomenon, in contrast to the phosphorescence at 488 nm. It is important to note that this emission is absent in the dilute frozen glass, in which we can assume that the emission resembles that of the isolated molecules. Furthermore, when **3** is embedded in a poly(methyl methacrylate) (PMMA) matrix, RTP is only observed in very highly doped films (≥50 wt %, Figure 2 c), further confirming the critical role of aggregation for this emission (Supporting Information, Figures S16–S20).

To understand the effect of the solid‐state structures and the intermolecular packing on the luminescence properties, the crystal structures of compounds **1**–**4** were obtained by single‐crystal X‐ray diffraction (Supporting Information, Figures S39–S42). If we compare the molecular geometries of compounds **1**–**4** in their crystal structures, we can observe the influence of additional methyl groups on the phenyl rings close to the central boron atom. While the B−C bond distances lie in a similar range for the bulkier *m*‐xylyl and mesityl groups (1.576–1.587 Å), the B−C (aryl) distances to the *o*‐tolyl group (B−C=1.570(2) Å in compound **2**) and the phenyl ring (B−C=1.569(2) Å in compound **1**) are slightly shorter (Supporting Information, Table S7). The effect of the bulkiness of the substituent and, hence, repulsion between methyl groups is further observed in the torsion angles between the aryl groups and the BC3 planes. While the torsion angles are in a similar range (50.0–54.9°) for the *m*‐xylyl and mesityl groups in compounds **3** and **4**, a significantly smaller torsion angle (41.9°) is observed for the *o*‐tolyl group in compound **2**, and a very small torsion angle of only 16.1° is observed for the phenyl group in compound **1**. These smaller torsion angles are compensated by larger torsion angles (56.7–65.3°) for the *m*‐xylyl groups in compounds **1** and **2** compared to those of compounds **3** and **4** (Table S7).

**Figure 5:**
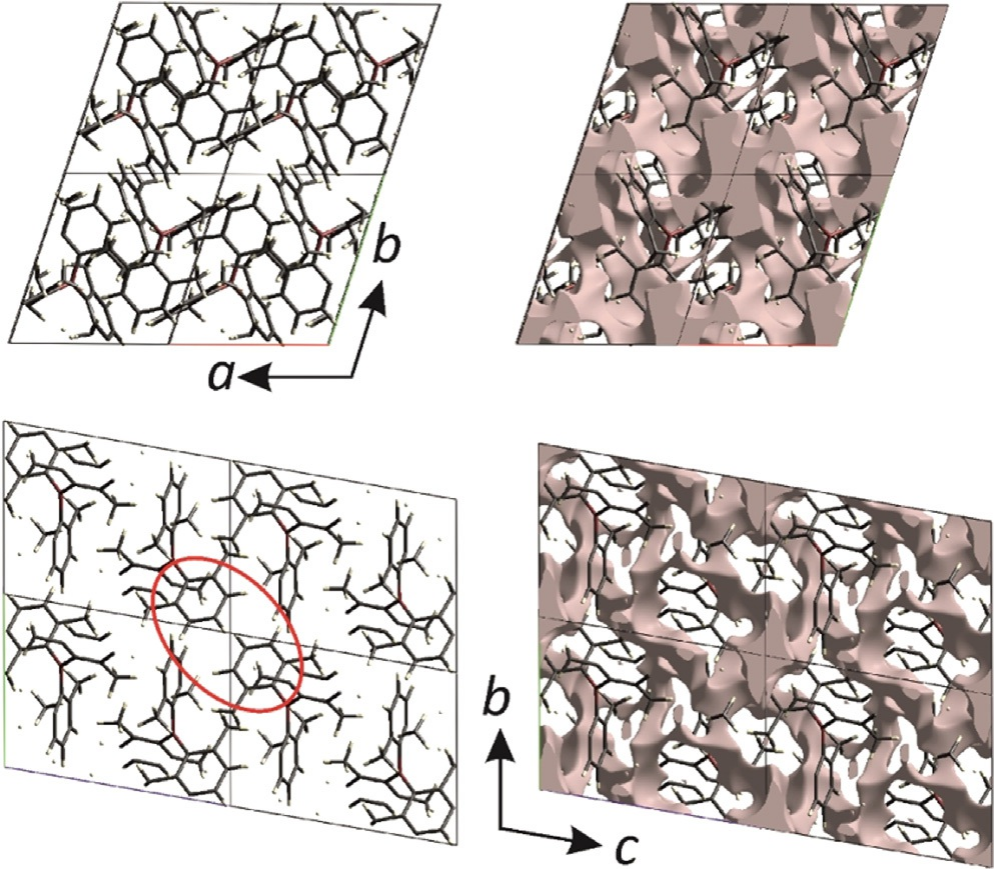
Crystal structure of compound **3** (left) projected along the *c* axis (top) and along the *a* axis (bottom), and plot of the surface of the crystal voids (0.002 au) from the Hirshfeld analysis (right). Four unit cells are shown in each case. Note the larger voids around the origin of the unit cell, as best observed in the centers of the drawings. The red ellipse encloses the aryl rings that are involved in a weak π⋅⋅⋅π interaction.

In order to compare and classify the types and magnitudes of the intermolecular interactions within single crystals of these four triarylboranes, which organize in a complex three‐dimensional arrangement, the concept of Hirshfeld surface analysis was applied (see Supporting Information for more details).^20^ The Hirshfeld surface is a special isosurface defined by the weighting function *w*(**r**)=0.5 for a particular molecule. This means that the Hirshfeld surface envelops the volume within which the particular molecule contributes more than half of the electron density. Hence, it also includes information on the nearest neighbors and closest contacts to the molecule. The molecules are most densely packed in compound **2**, as is clear from both the crystal packing coefficient *c*_k_, which corresponds to the ratio of volume occupied by all molecules in the unit cell to the unit cell volume, and the surface of the crystal's void per formula unit, which is obtained from the Hirshfeld analysis (Supporting Information, Table S8).^21^ Interestingly, compounds **1** and **3** show similar, intermediate packing densities, while compound **4** seems to have the loosest packing. While the surfaces of the voids seem to be spread well throughout the unit cells of compounds **1**, **2**, and **4**, a larger void of 9 Å^3^ is present in compound **3** around the origin of the unit cell (Figure 5). From comparison of the fluorescence emissions of compounds **1**–**4**, we can conclude that the RTP is not correlated with the packing density, as compound **2** is the densest packed compound. A deeper insight into the intermolecular interactions is required in order to provide an interpretation of the observed differences in emission behavior. Fingerprint analysis of the Hirshfeld surface and its breakdown into the individual relative contributions in crystals of **1**–**4**,^22^ exhibited a strong contribution of H⋅⋅⋅H interactions (75–83 %), followed by a significant amount of C⋅⋅⋅H interactions (17–25 %) in all four compounds (Supporting Information, Figures S4 and S44). Only a very weak contribution of C⋅⋅⋅C interactions is observed for compound **3** (0.2 %). While this analysis shows the relative contributions of the different types of intermolecular interactions, we are now interested in their strengths in the individual crystal structures. Compounds **1** and **3** exhibit several significant intermolecular C−H⋅⋅⋅C interactions, including strong, nearly linear interactions (C⋅⋅⋅H=2.835–2.841 Å, C−H⋅⋅⋅C=164–168°, Table S9 in the Supporting Information). In addition, compound **1** has a short H⋅⋅⋅H contact (2.241 Å) between two aryl rings, which is also demonstrated by the spike in the bottom left corner of its fingerprint plot (Supporting Information, Figure S44).

Compound **2**, although more densely packed than **1** and **3**, shows significantly fewer and weaker intermolecular C−H⋅⋅⋅C interactions. In addition, it shows a nearly linear, weak C−H⋅⋅⋅π interaction towards the centroid of an *m*‐xylyl ring (H⋅⋅⋅*π*=2.907 Å) and two close C⋅⋅⋅C contacts (C⋅⋅⋅C=3.334 and 3.384 Å), a strong one between two aryl rings, and a weak one between the same aryl and a methyl group (Table S9). These results are consistent with our analysis of the fluorescence emission in the crystalline states, wherein compounds **1** and **2** have the same emission maxima although one more methyl group is introduced to the phenyl ring in compound **2**. This may be explained by the presence of more and stronger interactions in **1** than in **2**. In crystals of compound **4**, intermolecular interactions are the weakest (Table S9). This is in agreement with the loosest packing mode. In addition to the strong C−H⋅⋅⋅C interactions, compound **3** also has a strong C⋅⋅⋅C interaction (C6⋅⋅⋅C6=3.319 Å) between two aryl rings with an approximately parallel alignment of their planes. This is the shortest nearest‐neighbor (nn) C⋅⋅⋅C distance in all of the compounds. The interplanar separation between the aryl planes is only 2.980 Å; however, the offset shift is large (4.221 Å), resulting in a centroid‐to‐centroid distance of 5.167 Å, the latter two values being too large for a typical offset face‐to‐face π⋅⋅⋅π stacking interaction between two arenes (Supporting Information, Table S10), which typically have values ranging from 3.3–3.8 Å for the interplanar separation, <4.0 Å for the offset, and <5.0 Å for the centroid‐to‐centroid distance.^23^ There is another arrangement of nearly parallel aryl rings, which has a longer C⋅⋅⋅C distance (3.495 Å) and interplanar separation (3.397 Å), but a smaller shift (3.493 Å) and, hence, a smaller centroid‐to‐centroid distance of 4.872 Å, all of those values being within the typical range of weak π⋅⋅⋅π interactions. The aryl rings, and hence the π⋅⋅⋅π interaction, are situated close to the voids, which are around the origin (Figure 5). It is proposed that, on compression of the crystal structure, the voids may shrink and, hence, the offset may also be reduced, enhancing the π⋅⋅⋅π interaction between these aryl rings. On the other hand, expansion of the molecule may also bring the rings closer together and enhance the π⋅⋅⋅π interaction. We assume that the aggregation of molecules forming C−H⋅⋅⋅C and π⋅⋅⋅π interactions is important for effective RTP in compounds **1** and **3**. A C⋅⋅⋅C offset aryl–aryl interaction is also present in both compounds **2** and **4** (Supporting Information, Table S10); however, the C−H⋅⋅⋅C interactions are much weaker in these compounds. In summary, the presence of both strong C−H⋅⋅⋅C and C⋅⋅⋅C contacts as well as weak π⋅⋅⋅π interactions in compound **3**, together with the void accumulation at the origin of the unit cell (Figure 5) may be the reason for the strong redshift and persistence of the aggregation‐induced phosphorescence emission of these crystals at room temperature and in highly doped PMMA‐films.

## Conclusion

We have prepared triarylboranes without lone pairs which exhibit long‐lived room‐temperature phosphorescence in the crystalline state and in highly doped PMMA films. Theoretical calculations revealed that the ISC process can be accelerated by transitions between local σ and π excitation, which is consistent with photophysical studies of the isolated molecules in a frozen glass and is an extension of El‐Sayed's rule. Moreover, the phosphorescent compounds **1** and **3** have the strongest interactions, especially when considering C−H⋅⋅⋅C interactions, which appear to play an important role in achieving persistent RTP and, at the same time, suppressing nonradiative decay. However, compounds **2** and **4** have fewer and weaker contacts in their crystalline states, and their nonradiative decay is fast, even though compound **2** has the densest packing. Thus, we do not observe RTP from crystals of compounds **2** and **4**. This study on triarylboranes provides an interesting example of how to expand the scope of purely organic phosphorescent materials.

## Conflict of interest

The authors declare no conflict of interest.

## Supporting information

As a service to our authors and readers, this journal provides supporting information supplied by the authors. Such materials are peer reviewed and may be re‐organized for online delivery, but are not copy‐edited or typeset. Technical support issues arising from supporting information (other than missing files) should be addressed to the authors.

Supplementary
