# Supplementary material for: Persistent Room Temperature Phosphorescence from Triarylboranes: A Combined Experimental and Theoretical Study
Source: Angew Chem Int Ed Engl. 2020 Aug 4;59(39):17137–44. doi: 10.1002/anie.202007610 (PMC7540320; doi:10.1002/anie.202007610)
Supplement: Supplementary file 1 — Supplementary [file ANIE-59-17137-s001.pdf]

Angewandte Chemie
Eine Zeitschrift der Gesellschaft Deutscher Chemiker

Supporting Information

Persistent Room Temperature Phosphorescence from Triarylboranes: A Combined Experimental and Theoretical Study

Zhu Wu, Jörn Nitsch, Julia Schuster, Alexandra Friedrich, Katharina Edkins, Marcel Loebnitz, Fabian Dinkelbach, Vladimir Stepanenko, Frank Würthner, Christel M. Marian, Lei Ji,* and Todd B. Marder**

anie_202007610_sm_miscellaneous_information.pdf

Supporting Information

Table of Contents

I. General information..........S2

II. Experimental procedures and characterization..........S5

III. DFT calculations..........S9

IV. Experimental photophysical spectra..........S24

V. Single-crystal X-ray diffraction..........S40

VI. ^{1}H, $^{13}C\{^{1}H\}$, ^{11}B NMR spectra, GC-MS and HRMS..........S57

VII. References..........S71

I. General information

Compounds **1-4** were prepared according to reported literature procedures.[1] Details are shown below. All starting materials were purchased from commercial sources and were used without further purification. The organic solvents for synthetic reactions and for photophysical measurements were HPLC grade, further treated to remove trace water using an Innovative Technology Inc. Pure-Solv Solvent Purification System and deoxygenated using the freeze-pump-thaw method. All synthetic reactions were performed in an Innovative Technology Inc. glovebox or under an argon atmosphere using standard Schlenk techniques. ^{1}H, ^{13}C and ^{11}B NMR spectra were measured on a Bruker Avance 500 MHz (^{1}H, 500 MHz; ^{13}C, 126 MHz; ^{11}B, 160 MHz) or Bruker Avance III 400 MHz (^{1}H, 400 MHz; ^{13}C, 101 MHz; ^{11}B, 128 MHz) NMR spectrometer. Mass spectra were recorded on Agilent 7890A/5975C Inert GC/MSD systems operating in EI mode. Elemental analyses were performed on a Leco CHNS-932 Elemental Analyser.

General photophysical measurements. All measurements were performed in standard quartz cuvettes (1 cm × 1 cm cross-section) except for ball-milled powder samples. They were prepared on 1 x 5 cm quartz plate (in air) or in a sealed cuvette (under argon). The emission signal showed no decrease in intensity or shape even after continuous irradiation over a period of 4 h. Samples recovered after each measurement showed the same photophysical properties when measured again, so there was no evidence for (photo)decomposition. UV-visible absorption spectra were recorded using an S6 Agilent 8453 diode array UV-visible spectrophotometer. The molar extinction coefficients were calculated from three independently prepared samples in hexane solutions. The emission spectra were recorded using an Edinburgh Instruments FLSP920 spectrometer equipped with a double monochromator for both excitation and emission, operating in right angle geometry mode, and all spectra were fully corrected for the spectral response of the instrument. All solutions used in photophysical measurements had concentrations lower than 10^{-5} M to minimize inner filter effects during fluorescence measurements.

Quantum yield measurements. The photoluminescent quantum yields were measured using a calibrated integrating sphere (inner diameter: 150 mm) from Edinburgh Instruments combined with the FLSP920 spectrometer described above.

For solution-state measurements, the longest-wavelength absorption maximum of the compound in the hexane was chosen as the excitation wavelength. For solid-state measurements, the excitation wavelength was 305 nm. The phosphorescence quantum yield of compounds **1** and **3** were obtained using the equation:

$$\Phi_{\mathrm{P}} = \frac{B}{A} \times \Phi_{\mathrm{PL}}$$

where A and B represent the integrated areas of the total photoluminescence and phosphorescence spectra, respectively. For the phosphorescence quantum yields, the phosphorescence component was separated from the total photoluminescence (PL) spectrum based on the phosphorescence spectrum obtained separately. Φ_{PL} represents the absolute photoluminescence quantum yields of compounds in solid state.

Lifetime measurements. Fluorescence lifetimes were recorded using the time-correlated single-photon counting (TCSPC) method using an Edinburgh Instruments FLSP920 spectrometer equipped with a high speed photomultiplier tube positioned after a single emission monochromator. Measurements were made in right-angle geometry mode, and the emission was collected through a polarizer set to the magic angle. Solutions were excited with a pulsed diode laser at a wavelength of 316 nm at repetition rates of 10 or 20 MHz. The instrument response functions (IRF) were *ca.* 230 ps FWHM. The phosphorescence lifetimes were measured using a μF920 pulsed 60 W Xenon microsecond flashlamp, with a repetition rate of 0.2 Hz at room temperature and 0.1 Hz at 77 K. Decays were recorded to 10000 counts in the peak channel with a record length of at least 2000 channels. Iterative reconvolution of the IRF with one decay function and non-linear least-squares analysis were used to analyze the data. The quality of all decay fits was judged to be satisfactory, based on the calculated values of the reduced χ^2 and Durbin-Watson parameters and visual inspection of the weighted residuals.

Scanning Electron Microscopy SEM images were recorded using a Zeiss Ultra Plus field emission scanning electron microscope equipped with GEMINI e-Beam column operated at 1-3 kV with an aperture size set to 10 or 30 μm to avoid excessive charging and radiation damage of the areas imaged.

Powder X-ray diffraction and Phase analysis Compound **3** was ground into a powder using an agate mortar until almost no room-temperature phosphorescence was observed (sample **A**). In addition, compound **3** was placed in a stainless steel vial (2 mL) and ground with 5 steel balls (3 mm diameter) for 5 minutes at a frequency of 15 Hz in a Lab Wizz LMLW 320/2 ball mill (sample **B**). Powder X-ray diffraction patterns were collected from two parts of each of the manually ground and the ball-milled powder samples in reflection geometry on a Bruker D8 Discover powder diffractometer with Da Vinci design and linear Lynx-Eye detector. X-ray radiation (Cu-Kα_1; λ = 1.5406 Å) was focused with a Goebel mirror and Cu-Kα_2 radiation was eliminated by a Ni-absorber. Data were collected from 5 – 60° 2θ in steps of 0.025° at ambient temperature. They were corrected for an offset in 2θ and exported using the Bruker AXS Diffrac-Suite. The diffraction patterns were then converted using CMPR software[2] for further processing with the GSAS program.[3] Cell parameters, background, scaling factor, zero shift and profile parameters were refined using the LeBail method. The starting values for the refinement were taken from the single-crystal structure refinement at 100 K.

Crystal structure determination Crystals suitable for single-crystal X-ray diffraction were selected, coated in perfluoropolyether oil, and mounted on MiTeGen sample holders. For the data collection at room temperature, a crystal was glued onto the holder. Diffraction data were collected on Bruker X8 Apex II 4-circle diffractometers with CCD area detectors using Mo-Kα radiation monochromated by graphite or multi-layer focusing mirrors. The crystals were cooled using an Oxford Cryostream low-temperature device. Data for compounds **1**, **2**, **3**, and **4** were collected at 100 K. Additional data for compound **3** were collected at 293, 240, 180, 120, and 83 K. The images were processed and corrected for Lorentz-polarization effects and absorption as implemented in the Bruker software packages. The structures were solved using the intrinsic phasing method (SHELXT)[4] and Fourier expansion technique. All non-hydrogen atoms were refined in anisotropic approximation, with hydrogen atoms 'riding' in idealized positions, by full-matrix least squares against F^2 of all data, using SHELXL[5] software and the SHELXLE graphical user interface.[6] Diamond[7] software was used for graphical representation. Other structural information was extracted using Mercury[8] and OLEX2[9] software. Hirshfeld surfaces were calculated and analyzed using the Crystal Explorer[10] program. Crystal

data and experimental details are listed in Table S6 for all compounds at 100 K and in Table S11 for compound **3** at other temperatures; full structural information has been deposited with Cambridge Crystallographic Data Centre. CCDC-1940099 (**1**), CCDC-1940100 (**2**), CCDC-1940101 – 1940106 (**3**), and CCDC-1940107 (**4**).

II. Experimental procedures and characterization

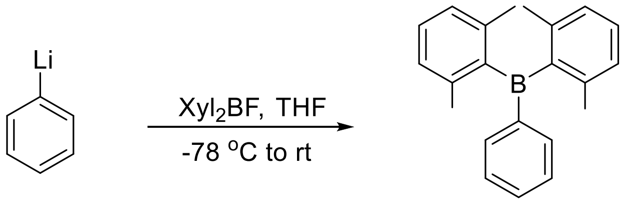

1

Bis(2,6-dimethylphenyl)(phenyl)borane (1): To a solution of bis(2,6-dimethylphenyl)-fluoroborane[11] (720 mg, 3.0 mmol) in anhydrous THF (20 mL) a hexane solution of PhLi (1.9 mL, 1.6 M, 3.1 mmol) was added dropwise by syringe at -78 °C. The reaction mixture was warmed to room temperature and stirred overnight. The reaction was quenched with a saturated solution of NaCl and the aqueous layer was extracted with Et_2O. The combined organic layer was dried over anhydrous Na_2SO_4, filtered, and concentrated under reduced pressure. The resulting crude material was subjected to silica gel column chromatography using *n*-hexane as eluent to afford 518 mg (1.74 mmol, 58%) of **1** as a white solid. Compound **1** (0.2 g) was dissolved in 2 mL of hexane. The solution was then transferred to two 1 mL GC vials into which hexane vapor was diffused at room temperature. Block-shaped crystals suitable for single-crystal X-ray diffraction formed after 48 h.

^{1}H NMR (400 MHz, $CDCl_3$, r.t., ppm): δ 7.53-7.47 (m, 3H), 7.38-7.34 (m, 2H), 7.24 (t, *J* = 8 Hz, 2H), 6.99 (d, *J* = 8 Hz, 4H), 2.05 (s, 12H). ^{13}C NMR (126 MHz, $CDCl_3$, r.t., ppm): δ 145.4 (br), 144.5 (br), 140.7, 136.5, 132.3, 129.0, 128.1, 127.3, 23.6. ^{11}B NMR (160 MHz, $CDCl_3$, r.t., ppm): δ 75 (br). MS (EI^+) m/z: 298 $[M]^+$. HRMS ($ASAP^+$): m/z calcd for $[C_{22}H_{23}B]$: 298.1887; found: 298.1882 (|Δ| = 1.68 ppm); Elem. Anal. Calcd (%) for $C_{22}H_{23}B$: C, 88.60; H 7.77; Found: C, 88.71; H, 7.92.

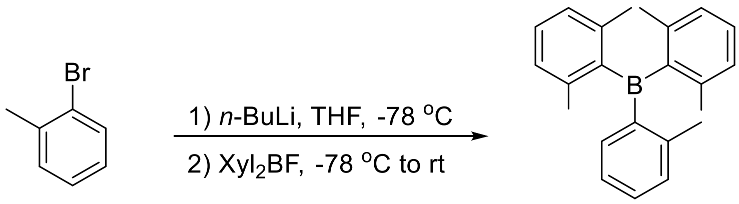

2

Bis(2,6-dimethylphenyl)(o-tolyl)borane (2): To a solution of 1-bromo-2-methylbenzene (462 mg, 2.7 mmol) in anhydrous THF (20 mL) a hexane solution of *n*-BuLi (1.7 mL, 1.6 M, 2.7 mmol) was added dropwise by syringe at -78 °C. The mixture was stirred at the same temperature for 1 h. A solution of bis(2,6-dimethylphenyl)fluoroborane (624 mg, 2.6 mmol) in anhydrous THF (5 mL) was added to the reaction mixture via syringe. The reaction mixture was warmed to room temperature and stirred overnight. The reaction was quenched with saturated solution of NaCl and the aqueous layer was extracted with Et_2O. The combined organic layer was dried over anhydrous Na_2SO_4, filtered, and concentrated under reduced pressure. The resulting crude material was subjected to silica gel column chromatography using *n*-hexane as eluent to afford 508 mg (1.63 mmol, 64%) of **2** as a white solid.

Compound **2** (0.2 g) was dissolved in 2 mL of hexane. The solution was then transferred to two 1 mL GC vials into which hexane vapor was diffused at room temperature. Block-shaped crystals suitable for single-crystal X-ray diffraction formed after 48 h.

^{1}H NMR (500 MHz, $CDCl_3$, r.t., ppm): δ 7.33-7.29 (t, *J* = 8 Hz, 1H), 7.21 (d, *J* = 8 Hz, 1H), 7.18-7.14 (m, 4H), 6.94 (d, *J* = 8 Hz, 4H), 2.08 (s, 3H), 2.02 (s, 12H). ^{13}C{^{1}H} NMR (126 MHz, $CDCl_3$, r.t., ppm): δ 147.4 (br), 146.0 (br), 142.7, 140.3, 135.0, 131.2, 130.0, 129.3, 127.5, 125.7, 23.2, 22.5. ^{11}B NMR (160 MHz, $CDCl_3$, r.t., ppm): δ 76 (br). MS (EI^+) m/z: 312 $[M]^+$. HRMS ($ASAP^+$): m/z calcd for $[C_{23}H_{25}B]$: 312.2158; found: 312.2145 (|Δ| = 4.16 ppm); Elem. Anal. Calcd (%) for $C_{23}H_{25}B$: C, 88.47; H, 8.07; Found: C, 88.72; H, 8.23.

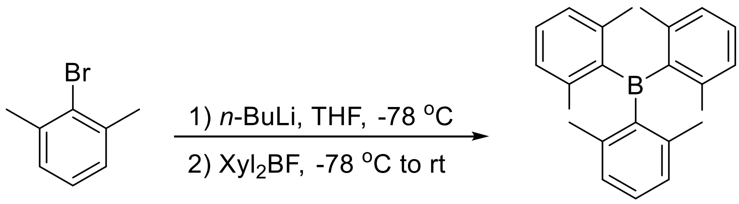

3

Tris(2,6-dimethylphenyl)borane (3): A solution of 2-bromo-m-xylene (1.1 mL, 8.3 mmol) in THF (10 mL) was treated with *t*-BuLi (1.7 M in pentane, 8.8 mL, 15 mmol) under argon at –78 °C. Then it was stirred at RT for 3 h before a solution of Xyl_2BF (1.8 g, 7.5 mmol) in THF (5 mL) was added at –78 °C and the reaction was allowed to warm to room temperature and stirred overnight. After 16 h, the reaction was quenched by adding 1 mL of water under argon. After removing the solvent and recrystallisation of the yellow solid from EtOH, the title compound was obtained as a white crystalline material (1.6 g, 64 %).

Compound **3** (0.2 g) was dissolved in 2 mL of hexane. The solution was then transferred to two 1 mL GC vials into which hexane vapor was diffused at room temperature. Block-shaped crystals suitable for single-crystal X-ray diffraction formed after 48 h.

^{1}H NMR (500 MHz, $CDCl_3$, r.t., ppm): δ 7.14 (t, J = 8 Hz, 3H), 6.91 (d, J = 8 Hz, 6H), 2.02 (s, 18H); ^{13}C{^{1}H} NMR: (75 MHz, $CDCl_3$) δ 147.2 (br), 140.9, 130.0, 128.2, 23.3. ^{11}B NMR (160 MHz, $CDCl_3$, r.t., ppm): δ 77 (br). MS (EI) m/z: 326. HRMS ($ASAP^+$): m/z calcd for [$C_{24}H_{27}B$]: 326.2200; found: 326.2194 (|Δ| = 1.84 ppm); Elem. Anal. calcd for $C_{24}H_{27}B$: C, 88.35; H, 8.34; Found C 88.51, H 7.86.

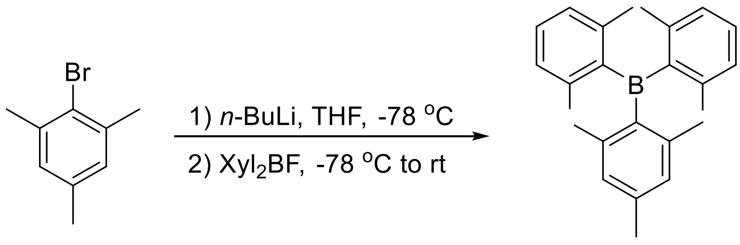

4

Bis(2,6-dimethylphenyl)(mesityl)borane (**4**): To a solution of 2-bromo-1,3,5-trimethylbenzene (617 mg, 3.1 mmol) in anhydrous THF (20 mL) a hexane solution of *n*-BuLi (1.9 mL, 1.6 M, 3.1 mmol) was added dropwise by syringe at -78 °C. The mixture was stirred at the same temperature for 1 h. A solution of bis(2,6-dimethylphenyl)fluoroborane (720 mg, 3.0 mmol) in anhydrous THF (5 mL) was added to the reaction mixture via syringe. The reaction mixture was warmed to room temperature and stirred overnight. The reaction was quenched with saturated solution of NaCl and the aqueous layer was extracted with Et_2O. The combined organic layer was dried over anhydrous Na_2SO_4, filtered, and concentrated under reduced pressure. The resulting crude material was subjected to silica gel column chromatography using *n*-hexane as eluent to afford 715 mg (2.1 mmol) of **4** in 71% yield as a white solid.

Compound **4** (0.2 g) was dissolved in 2 mL of hexane. The solution was then transferred to two 1 mL GC vials into which hexane vapor was diffused at room temperature. Block-shaped crystals suitable for single-crystal X-ray diffraction formed after 48 h.

^{1}H NMR (400 MHz, $CDCl_3$, r.t., ppm): δ 7.13 (t, *J* = 8 Hz, 2H), 6.91 (s, 2H), 6.90 (s, 2H), 6.75 (d, *J* = 1 Hz, 2H), 2.27 (s, 3H), 2.03 (s, 6H), 2.01 (s, 6H), 1.98 (s, 6H). ^{13}C{^{1}H} NMR (126 MHz, $CDCl_3$, r.t., ppm): δ 147.2 (br), 143.9 (br), 140.9, 140.5, 140.4, 139.6, 129.4, 128.8, 127.8, 127.8, 23.1, 22.9, 22.9, 21.4 ppm. ^{11}B NMR (160 MHz, $CDCl_3$, r.t., ppm): δ 78 (br). MS (EI^+) m/z: 340 $[M]^+$. HRMS ($ASAP^+$): m/z calcd for $[C_{25}H_{29}B]$: 340.2357; found: 340.2350 (|Δ| = 2.06 ppm); Elem. Anal. Calcd (%) for $C_{25}H_{29}B$: C, 88.23; H, 8.59; Found: C, 87.90; H, 8.74.

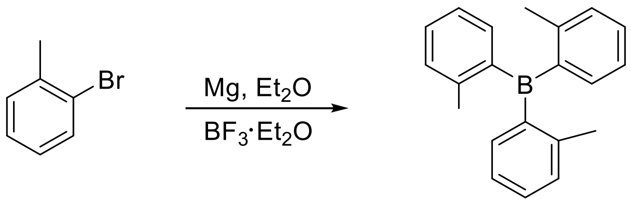

Tris(2-methylphenyl)borane: In a 200-mL, three-necked, round-bottomed flask maintained under argon and equipped with a reflux condenser, whose top was connected to an argon inlet and a pressure-equalizing dropping funnel, were placed 0.14 g (5.8 mmol) of magnesium turnings. With stirring, 50 mL of 3 g (17.5 mmol) of 1-bromo-2-methylbenzene anhydrous ether solution was added in one portion. The mixture was warmed to initiate the reaction and was then heated under reflux for another 2 h. Subsequently, 0.72 ml (5.8 mmol) $BF_3 \cdot Et_2O$ was added to the mixture. After 24 h, 150 mL of dry ether was added, while maintaining an argon atmosphere. The mixture was filtered and the filtrate was evaporated to dryness. The crude material was crystallized from pentane at –35 °C to afford 1.26 g (4.4 mmol, 76 %) of tris(2-methylphenyl)borane as white solids: ^{1}H NMR (500 MHz, $CDCl_3$, r.t., ppm): δ 7.34 (m, 3H), 7.18 (m, 9H), 2.10 (s, 9H). ^{13}C{^{1}H} NMR (126 MHz, $CDCl_3$, r.t., ppm): δ 146.3 (br.), 142.4, 134.8, 130.4, 129.8, 125.0, 23.1. ^{11}B NMR (160 MHz, $CDCl_3$, r.t., ppm): δ 73 (br). HRMS (ASAP$^+$): m/z calcd for [$C_{22}H_{21}B$]: 284.1731; found: 284.1723 (|Δ| = 2.82 ppm); Elem. Anal. Calcd (%) for $C_{22}H_{21}B$: C, 88.75; H, 7.45; Found: C, 88.51; H, 7.45.

III. DFT calculations

Computational methods and technical details for the quantum chemical calculations

The Turbomole program package was used for all geometry optimizations.[12] The equilibrium geometry of the electronic ground state was optimized with Kohn-Sham density functional theory (DFT)[13] employing the B3-LYP functional.[14] For the singlet excited state geometries, full linear response time dependent DFT (TD-DFT)[15] calculations were performed whereas the Tamm-Dancoff approximation was employed for the triplet states. Atomic orbitals were represented by split-valence basis sets with polarization functions (SVP) from the Turbomole basis set library.[16] Electronic excitation energies and transition dipole moments were calculated with a

redesigned variant of the DFT/MRCI[17] method. DFT/MRCI is a semi-empirical multireference configuration interaction approach based on Kohn-Sham orbitals and orbital energies of a closed shell BH-LYP[14b, 18] functional determinant. To avoid double counting of electron correlation, high-lying configurations with energies more than 1 E_h above the highest root in the reference space are discarded. The reference space was determined iteratively, starting with single and double excitations of 12 electrons within an active window of 12 frontier orbitals. At all geometries, 11 singlet and 10 triplet roots were determined. Absorption line spectra were broadened with Gaussians of 1000 cm^{-1} full width at half maximum (FWHM). Electronic spin–orbit coupling matrix elements (SOCMEs) and phosphorescence lifetimes were obtained with the SPOCK program.[19] Harmonic vibrational frequencies were determined numerically at the TDDFT or TDA level using the SNF code.[20] The FC profiles of the emission spectra were obtained using the Fourier transform approach implemented in the Vibes program.[21] Herein, a time interval of 300 fs and a grid of 16384 points were chosen. The correlation function was damped with a Gaussian function of 200 cm^{-1} FWHM and the temperature was set to 77 K. All spectra were normalized to one. Rate constants for intersystem crossing (ISC) of the respective S_1 states to the T_1 states of all compounds and a few other triplet states were computed for a temperature of 300 K using the Condon approximation. For the integration of the time correlation function in the VIBES program a time interval of 250 fs, an integration grid of 1000 points and smaller damping of width 0.3 cm^{-1} were chosen. The electronic SOCME is largest for the ISC from S_1 to T_2, but harmonic vibrational wavefunctions of the latter state could unfortunately not be determined due to (near) degeneracy of the T_1 and T_2 potentials at the T_2 minimum. For that reason, we used the modes of the T_1 (compounds **1** and **2**) or T_3 potentials (compounds **3** and **4**) to estimate the vibrational density of final states at the energies of the initial singlet states which are required in addition to the sum over squared SOCMEs to determine the ISC rate constants in the Condon approximation.[22]

Table S1. Spin–orbit coupling matrix elements (absolute values, cm^{-1}) of the S_1, T_1, and T_2 states at the respective S_1 minimum geometry.

SOCME	x	y	z	$(x^2 + y^2 + z^2)$
		**Compound 1**		
$\langle T_1 \| \hat{H}_{SO} \| S_1 \rangle$	0.039	0.149	0.232	0.078
$\langle T_2 \| \hat{H}_{SO} \| S_1 \rangle$	0.524	0.999	0.022	1.273
		**Compound 2**		
$\langle T_1 \| \hat{H}_{SO} \| S_1 \rangle$	0.154	0.167	0.147	0.073
$\langle T_2 \| \hat{H}_{SO} \| S_1 \rangle$	0.403	0.883	0.159	0.967
		**Compound 3**		
$\langle T_1 \| \hat{H}_{SO} \| S_1 \rangle$	0.024	0.039	0.002	0.002
$\langle T_2 \| \hat{H}_{SO} \| S_1 \rangle$	0.005	1.270	0.038	1.614
		**Compound 4**		
$\langle T_1 \| \hat{H}_{SO} \| S_1 \rangle$	0.048	0.060	0.030	0.007
$\langle T_2 \| \hat{H}_{SO} \| S_1 \rangle$	0.317	1.067	0.706	1.737

Table S2. Photophysical properties of compounds **1-4** in vacuum as obtained from quantum chemical calculations.

Compound	λ_{abs} /nm [a]	k_{isc} /s^{-1} [b]	λ_f /nm [c]	k_f/ s^{-1}	τ_f /ns [d]	λ_p /nm [c]	k_p / s^{-1}	τ_p /s [d]
**1**	303	≈$1*10^7$	(335), 383	$2*10^7$	45	(425), 597	0.13	8
**2**	308	≈$6*10^6$	368, 391	$2*10^7$	45	448, 476	0.13	8
**3**	314	≈$5*10^7$	367, 404	$3*10^7$	38	456, 486	0.14	7
**4**	316	≈$3*10^7$	381, 430	$4*10^7$	25	458, 489	0.20	5

[a] Absorption maximum of line spectrum broadened by Gaussian function of 1000 cm^{-1} full width at half maximum; [b] rate constant for the fastest of the open ISC channels; [c] maximum of 0-0 band, maximum of Franck-Condon spectrum at 77 K, entries given in parentheses indicate the position of a shoulder; [d] pure radiative lifetime assuming a quantum yield of **1**.

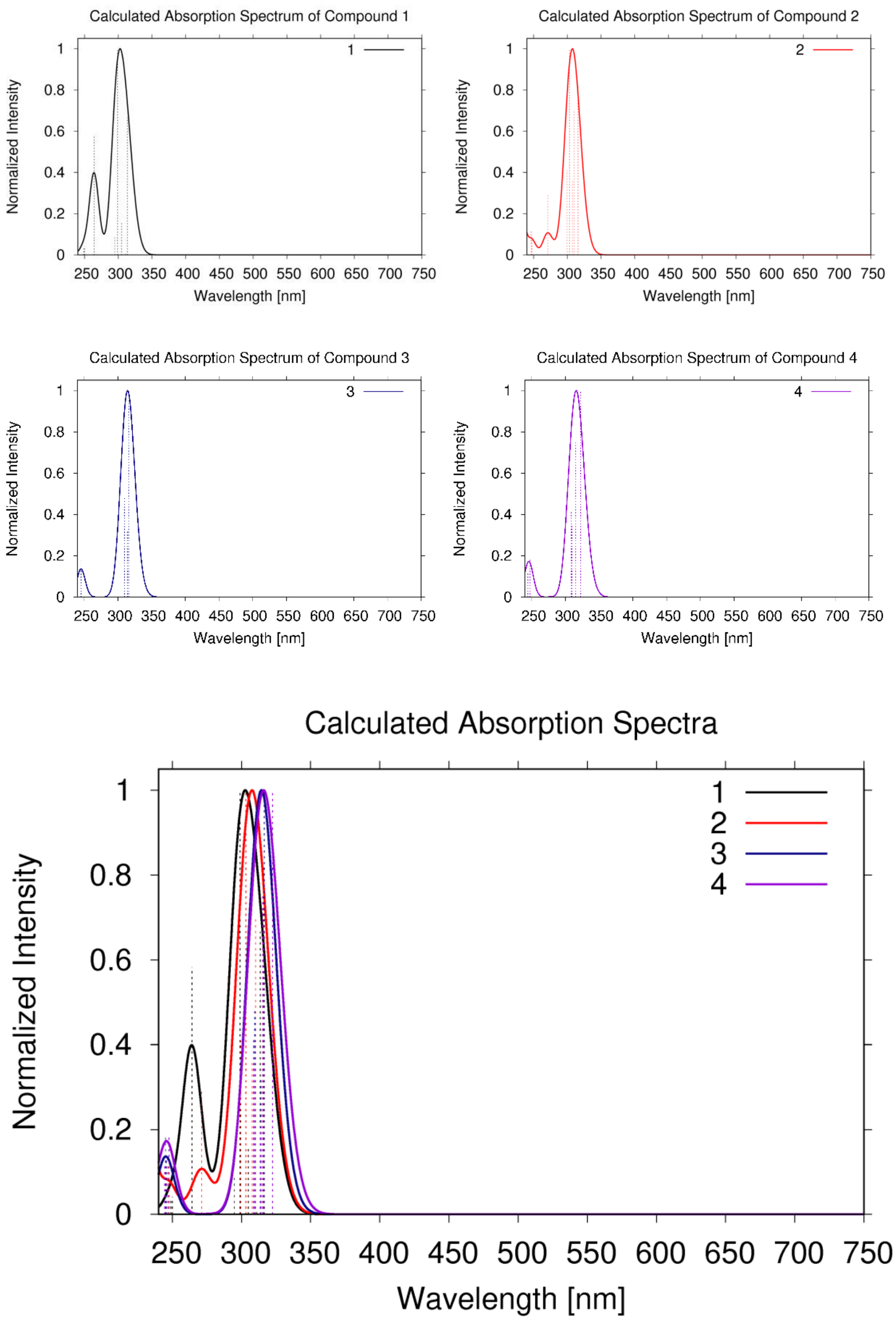

Figure S1. Calculated absorption spectra of the isolated compounds **1**, **2**, **3**, and **4**.

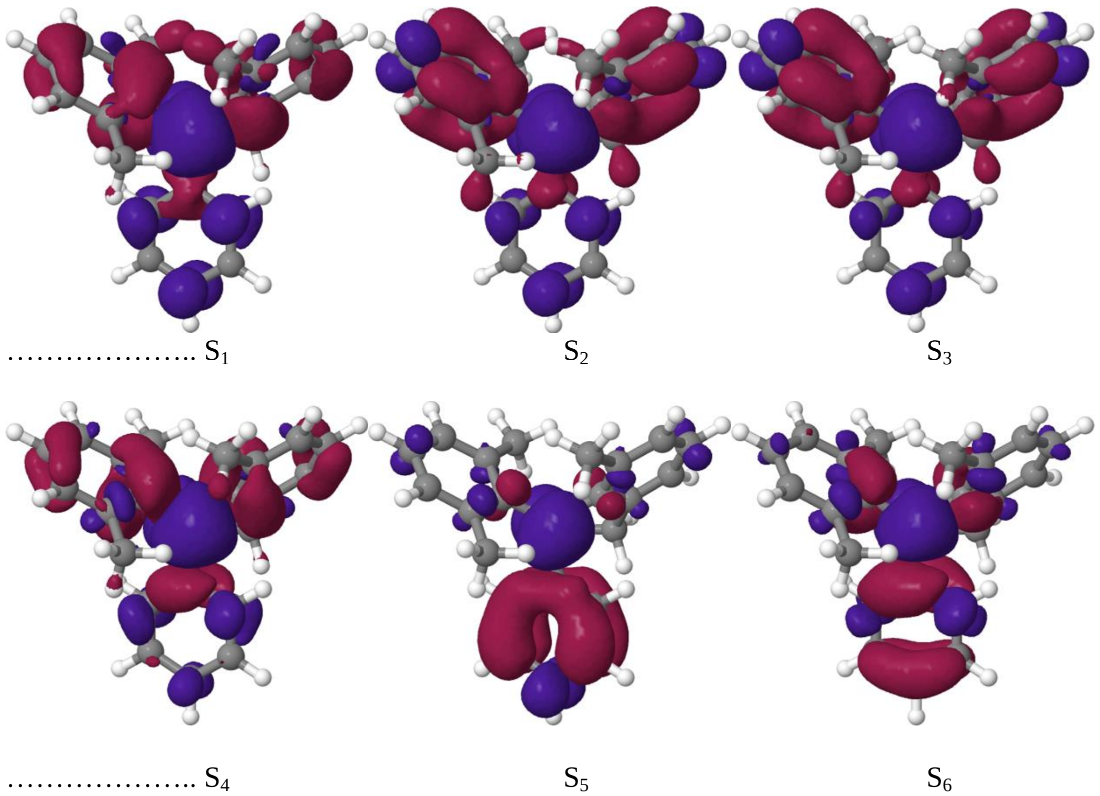

Figure S2. Difference densities (|isovalue| = 0.001) of low-lying singlet excited states of compound **1** at the DFT-optimized ground-state geometry. A loss of electron density with respect to the S_0 state is indicated in red, a gain in blue.

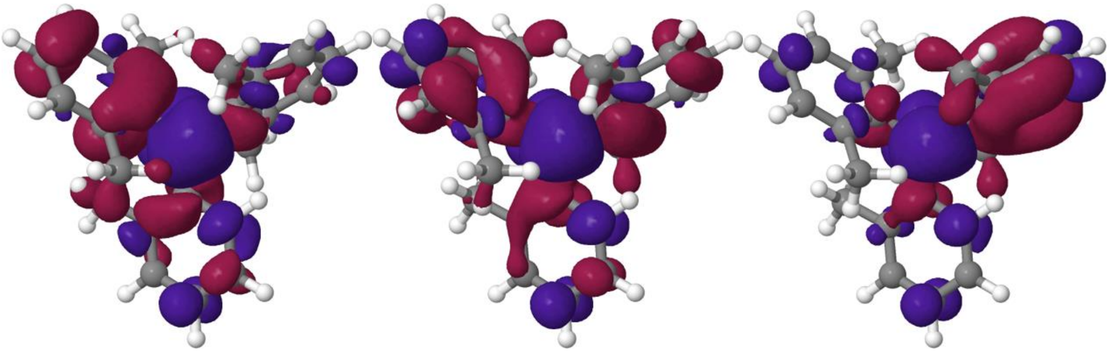

……………….. S_1 S_2 S_3

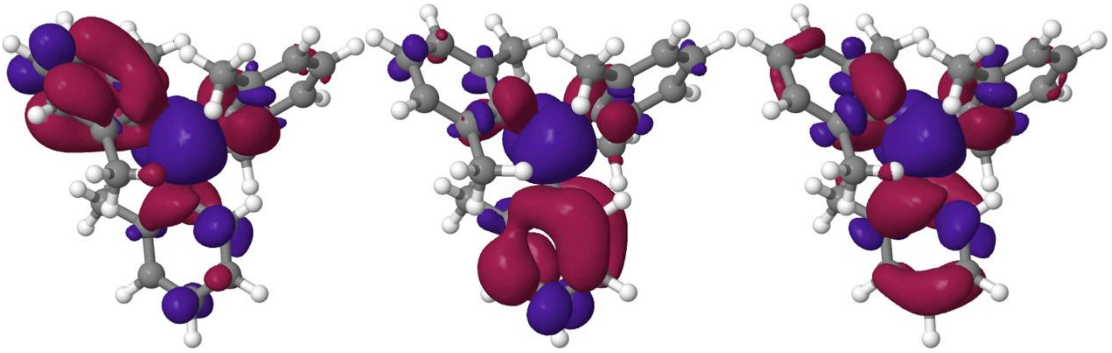

……………….. S_4 S_5 S_6

Figure S3. Difference densities (|isovalue| = 0.001) of low-lying singlet excited states of compound **2** at the DFT-optimized ground-state geometry. A loss of electron density with respect to the S_0 state is indicated in red, a gain in blue.

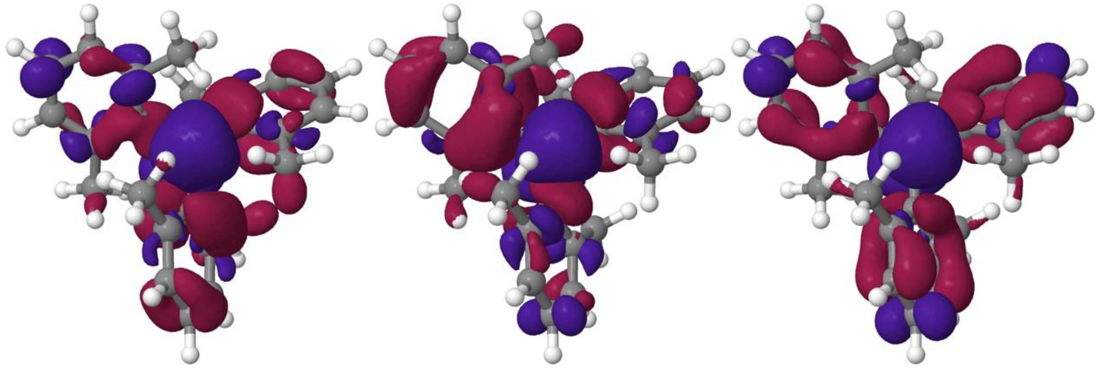

……………….. S_1 S_2 S_3

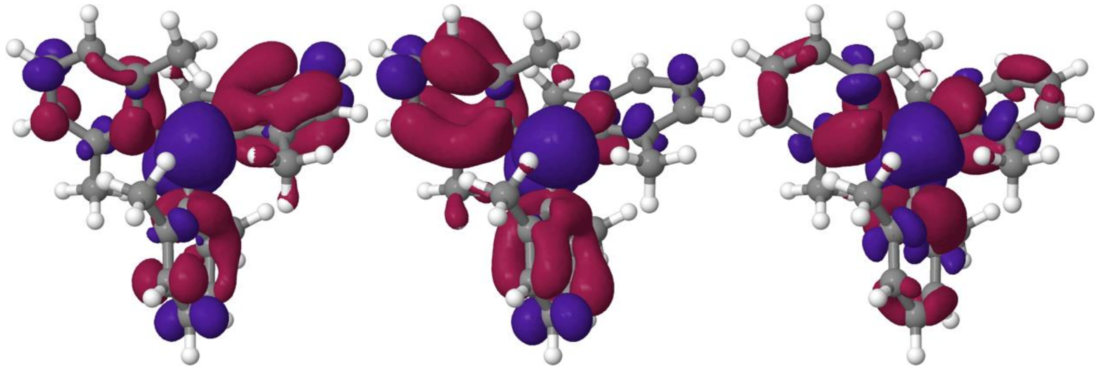

……………….. S_4 S_5 S_6

Figure S4. Difference densities (|isovalue| = 0.001) of low-lying singlet excited states of compound **3** at the DFT-optimized ground-state geometry. A loss of electron density with respect to the S_0 state is indicated in red, a gain in blue.

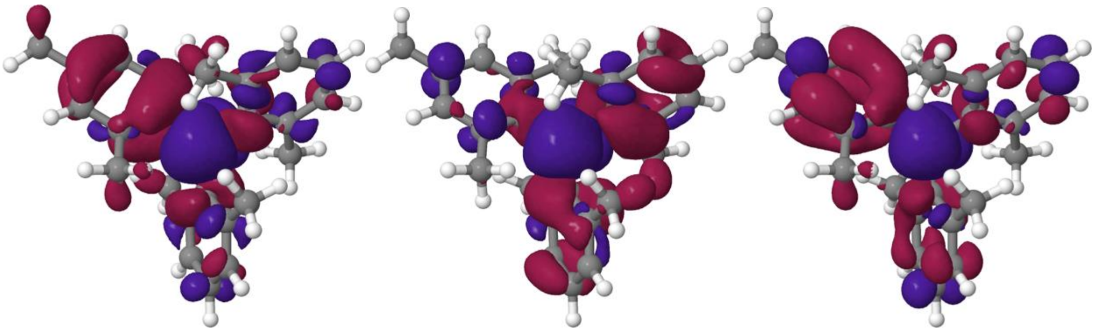

……………….. S_1 S_2 S_3

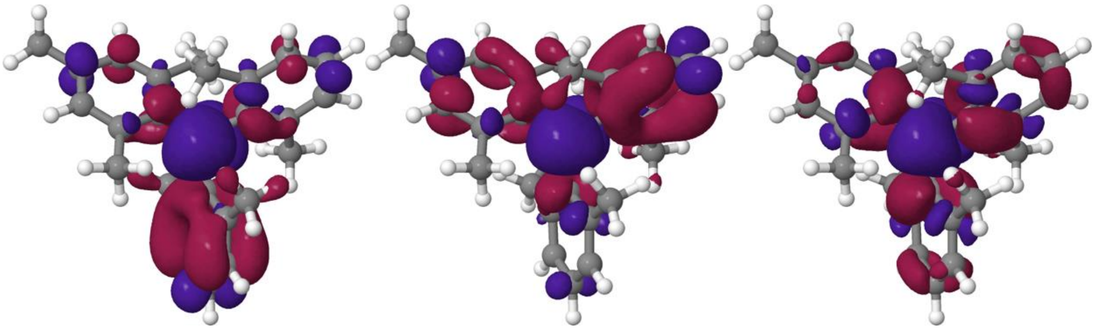

……………….. S_4 S_5 S_6

Figure S5. Difference densities (|isovalue| = 0.001) of low-lying singlet excited states of compound **4** at the DFT-optimized ground-state geometry. A loss of electron density with respect to the S_0 state is indicated in red, a gain in blue.

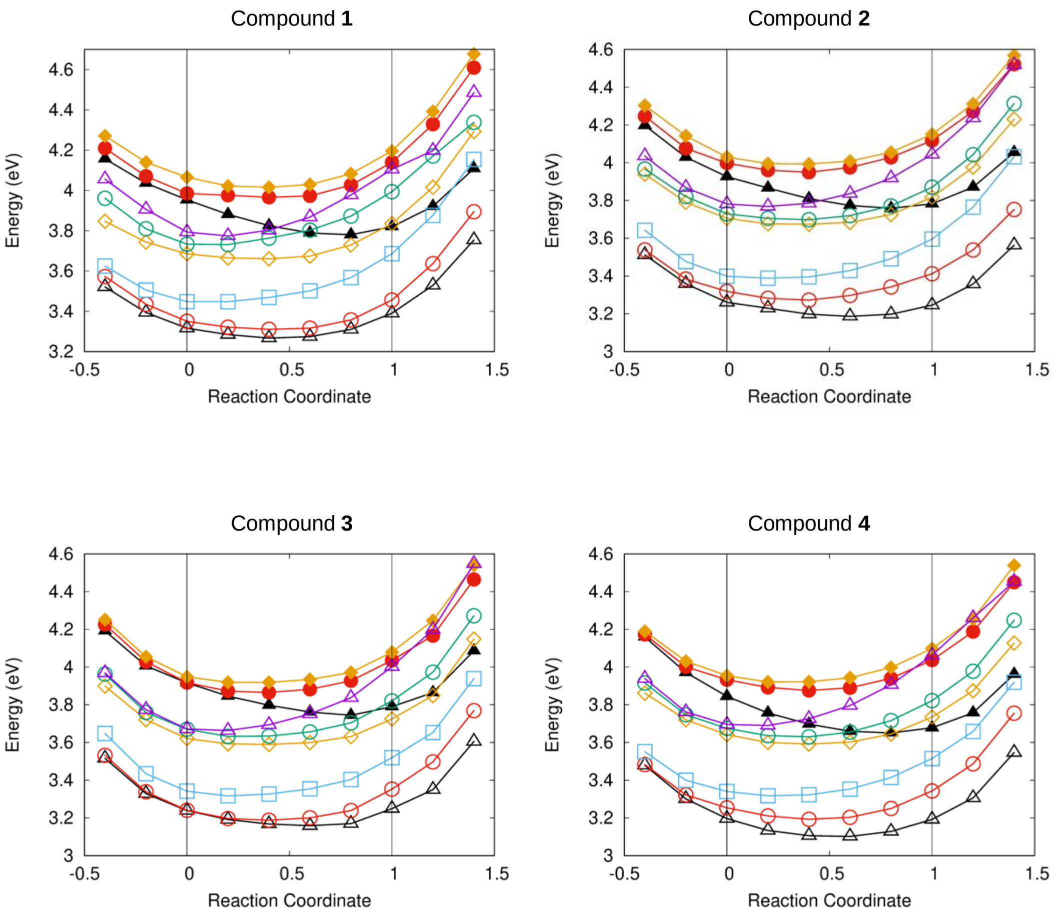

Figure S6. DFT/MRCI potential energy profiles of low-lying singlet (filled symbols) and triplet (open symbols) states of compounds **1**, **2**, **3**, and **4**. The reaction coordinate connects the DFT-optimized S_0 geometry (RC = 0) and the TDDFT-optimized geometry of the first excited singlet state S_1 (RC = 1) and is extrapolated on both sides. Potential energy curve crossings are observed between the S_1 state (full black triangles) and excited triplet states along the relaxation path from the Franck-Condon geometry.

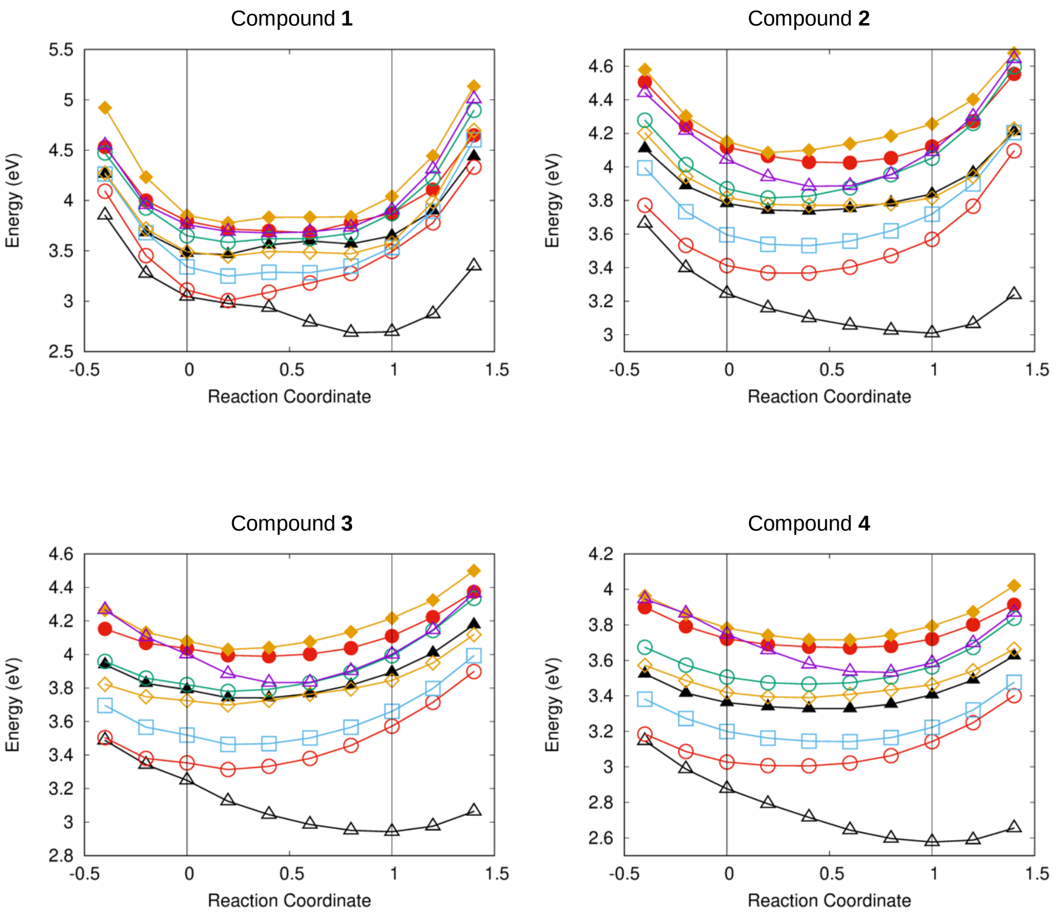

Figure S7. DFT/MRCI potential energy profiles of low-lying singlet (filled symbols) and triplet (open symbols) states of compounds **1**, **2**, **3**, and **4**. The reaction coordinate connects the TDDFT-optimized geometries of the S_1 (RC = 0) and T_1 (RC = 1) states and is extrapolated on both sides.

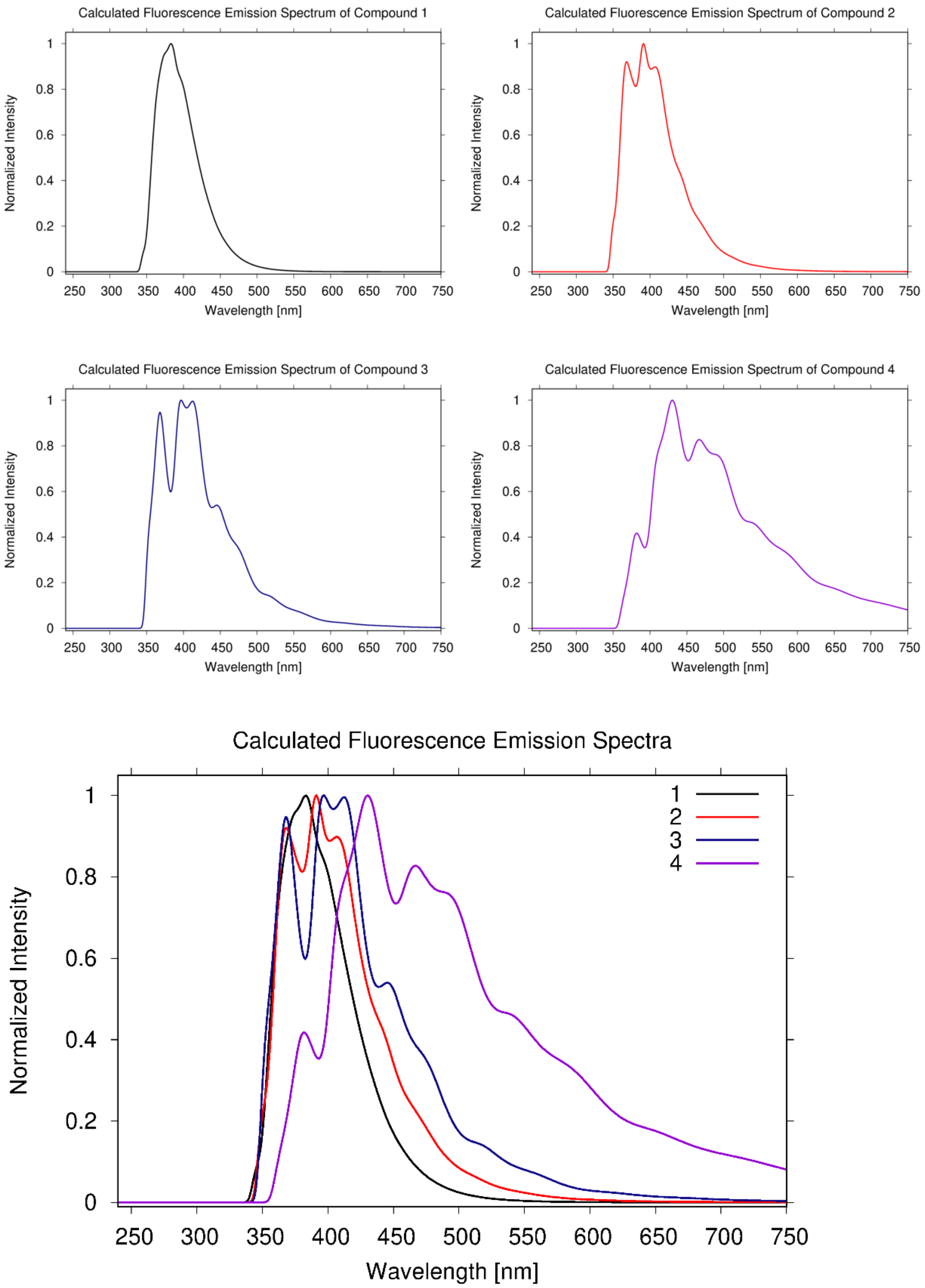

Figure S8. Calculated fluorescence spectra of the isolated compounds **1**, **2**, **3**, and **4** at 77 K.

Table S3. Selected bond lengths (Å) and angles (°) of the isolated compounds **1**, **2**, **3**, and **4** as obtained from geometry optimizations of the S_0, S_1, and T_1 states. The electronic excitation in the S_1 and T_1 states mainly involves residue R2.

1: R1=phenyl, R2=R3=xylyl
2: R1=tolyl, R2=R3=xylyl
3: R1=R2=R3=xylyl
4: R1=R3=xylyl, R2=mesityl

Compound **1**	S_0	S_1	T_1
B-R1	1.574	1.532	1.568
B-R2	1.589	1.620	1.567
B-R3	1.589	1.592	1.602
∠R1(C)-B-(C-C)R2	55.3	70.5	38.4
∠R2(C)-B-(C-C)R3	55.4	49.8	61.5
∠R3(C)-B-(C-C)R1	28.3	20.3	20.6
∠R2(C)-B-(C-C)R1	28.3	13.2	25.5
∠R3(C)-B-(C-C)R2	55.5	67.1	43.4
∠R1(C)-B-(C-C)R3	55.2	48.4	62.0
Compound **2**	S_0	S_1	T_1
B-R1	1.580	1.546	1.574
B-R2	1.588	1.610	1.562
B-R3	1.591	1.597	1.605
∠R1(C)-B-(C-C)R2	51.9	59.2	39.0
∠R2(C)-B-(C-C)R3	53.0	46.8	55.5
∠R3(C)-B-(C-C)R1	38.1	25.3	31.5
∠R2(C)-B-(C-C)R1	38.7	24.2	36.9
∠R3(C)-B-(C-C)R2	54.2	60.9	44.1
∠R1(C)-B-(C-C)R3	55.4	51.6	58.5
Compound **3**	S_0	S_1	T_1
B-R1	1.590	1.581	1.596
B-R2	1.590	1.611	1.557
B-R3	1.590	1.579	1.595
∠R1(C)-B-(C-C)R2	50.1	56.4	43.4
∠R2(C)-B-(C-C)R3	49.9	38.9	48.8
∠R3(C)-B-(C-C)R1	49.8	43.6	47.8
∠R2(C)-B-(C-C)R1	49.9	40.3	49.2
∠R3(C)-B-(C-C)R2	50.3	56.8	43.6
∠R1(C)-B-(C-C)R3	49.6	42.3	47.5
Compound **4**	S_0	S_1	T_1
B-R1	1.591	1.575	1.593
B-R2	1.587	1.619	1.564
B-R3	1.591	1.577	1.596
∠R1(C)-B-(C-C)R2	49.4	58.9	43.9
∠R2(C)-B-(C-C)R3	49.6	39.3	48.1
∠R3(C)-B-(C-C)R1	49.8	42.2	46.1
∠R2(C)-B-(C-C)R1	49.9	38.6	47.1
∠R3(C)-B-(C-C)R2	49.4	59.0	44.3
∠R1(C)-B-(C-C)R3	49.7	42.8	47.6

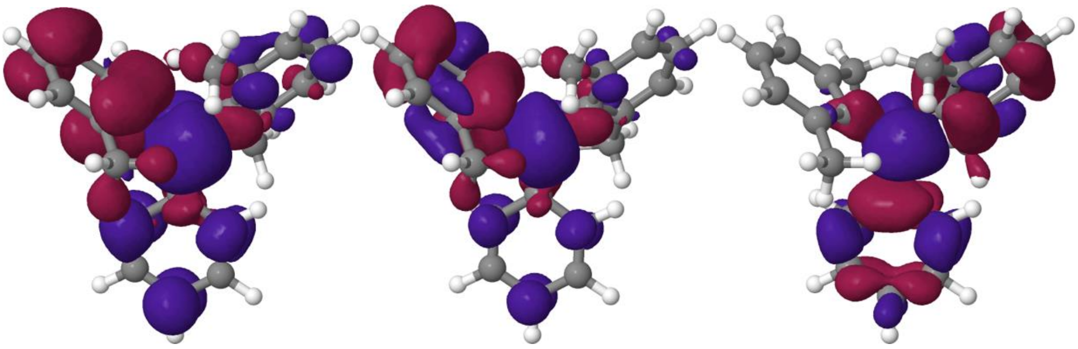

Compound **1** S$_1$ T$_1$ T$_2$

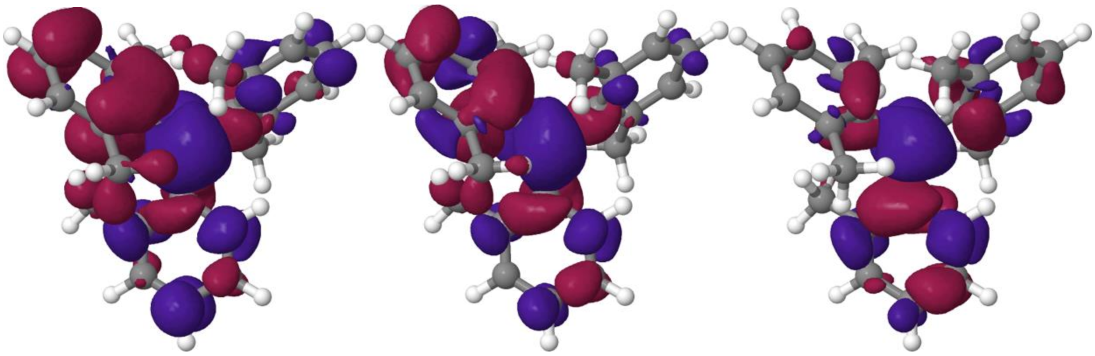

Compound **2** S$_1$ T$_1$ T$_2$

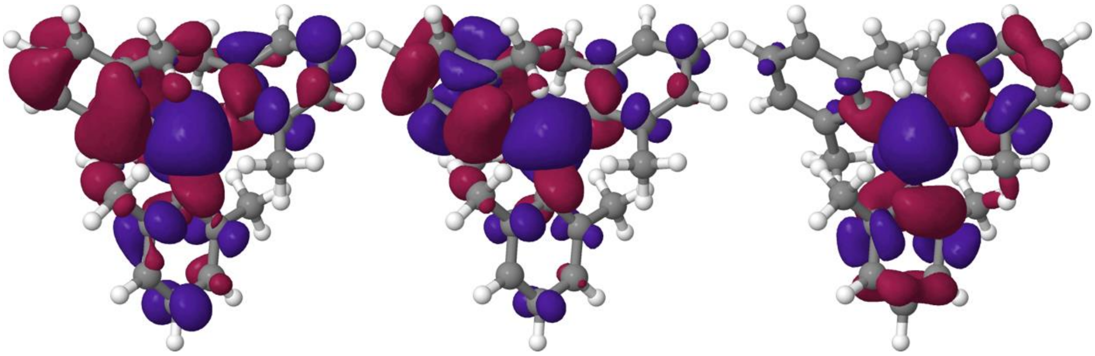

Compound **3** S$_1$ T$_1$ T$_2$

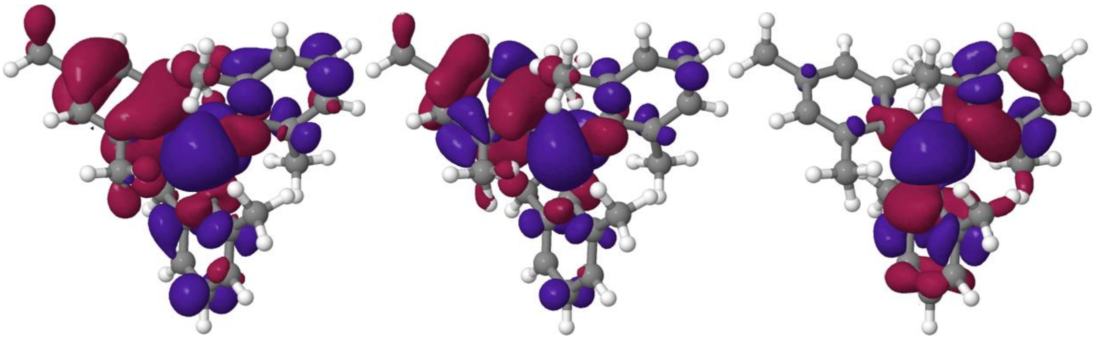

Compound **4** S$_1$ T$_1$ T$_2$

Figure S9. Difference densities (|isovalue| = 0.001) of low-lying excited states of compounds **1**, **2**, **3**, and **4** at the TDDFT-optimized geometry of the S$_1$ state. A loss of electron density with respect to the S$_0$ state is indicated in red, a gain in blue.

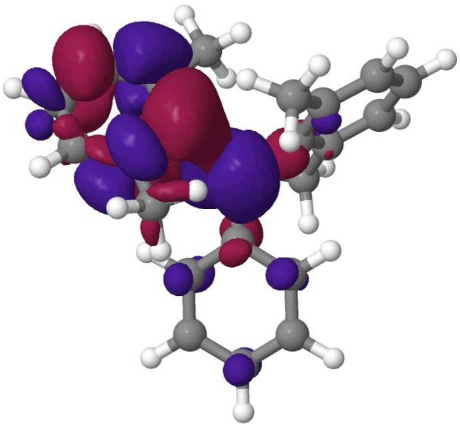

Compound **1** T_1

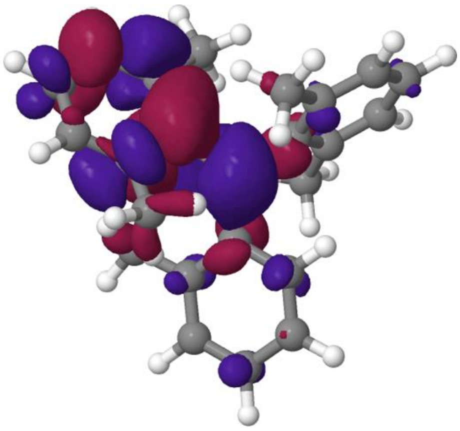

Compound **2** T_1

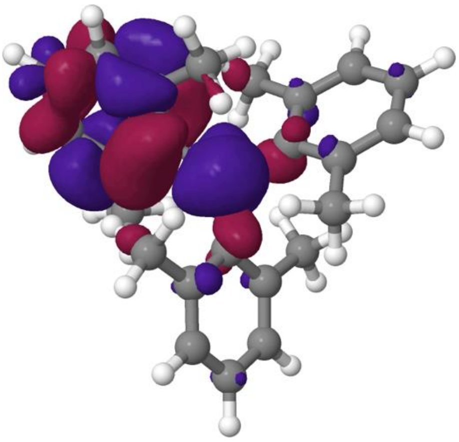

Compound **3** T_1

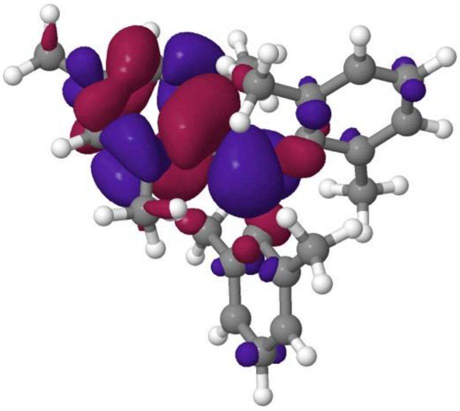

Compound **4** T_1

Figure S10. Difference densities (|isovalue| = 0.001) of the T_1 state of compounds **1**, **2**, **3**, and **4** at its TDDFT-TDA-optimized minimum geometry. A loss of electron density with respect to the S_0 state is indicated in red, a gain in blue.

Calculated minimum energy structures of the S_1 and T_1 states reveal opposite trends of the B–C bond lengths. In the S_1 state, the bond connecting boron and the aryl group from which electron density is transferred to boron (B-R2 in Table S3) is elongated upon excitation while the other two B–C bonds shorten with respect to the electronic ground-state structure. In the T_1 state, which exhibits more local (π, π*) contributions to the excitations than S_1, the first B–C bond shortens and the other two are elongated (Figures S9 and S10). In compounds 3 and 4, with methyl groups in the ortho positions of all aryl rings, the dihedral angles change by 10° at most in the S_1 state, and by less than 7° in T_1. Corresponding displacements of the minimum dihedral angles in compound 1 reach values up to 15° and 17°, respectively, and up to 14° and 13°, respectively, in compound 2.

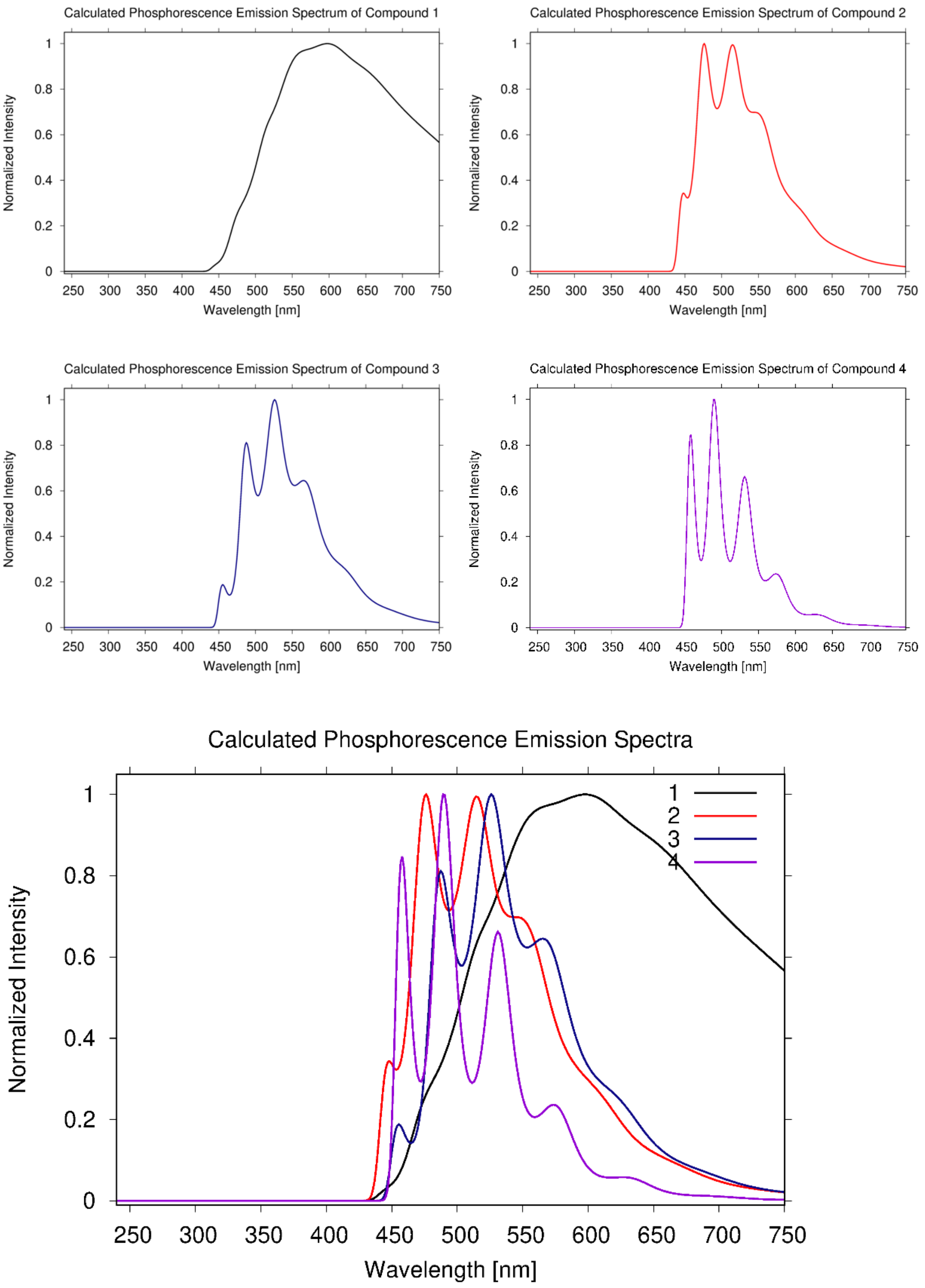

Figure S11. Calculated phosphorescence spectra of the isolated compounds **1**, **2**, **3**, and **4** at 77 K.

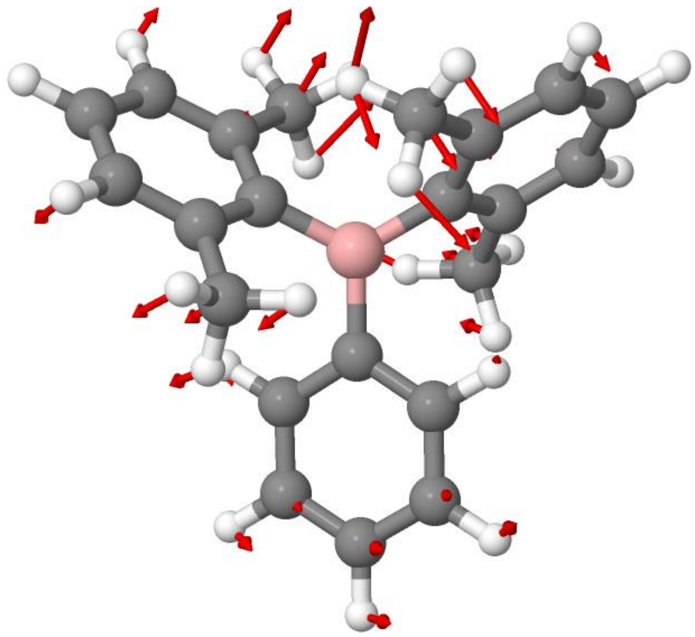

Figure S12. Low-frequency vibrational mode in the S_0 state of compound **1** showing the largest displacement with respect to the corresponding mode in the T_1 state. Excitation of this mode causes the extraordinary breadth of the phosphorescence emission of this compound. One methyl group in ortho-position of residue R3 is sufficient to prevent similar large-amplitude motions along in compound **2**.

IV. Experimental photophysical spectra

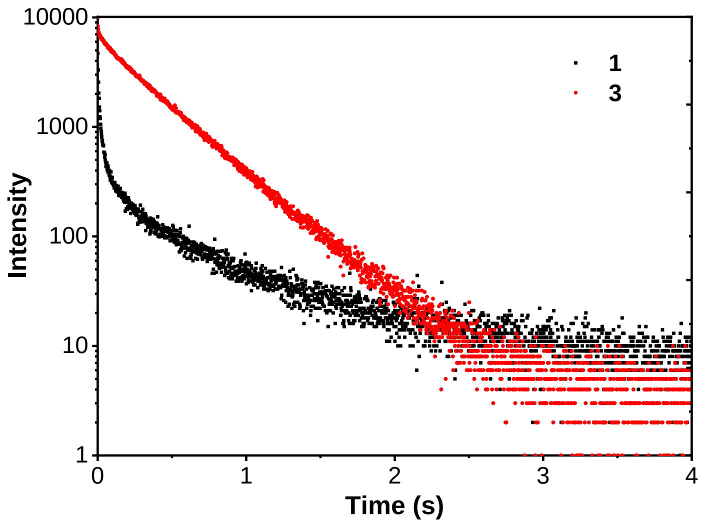

Figure S13. Decays of the phosphorescence emission from crystalline **1** (520 nm) and 3 (575 nm) at room temperature.

Table S4. Experimental photophysical properties of compounds **1-4** in hexane and crystalline state at RT, and in methylcyclohexane at 77 K.

Compound	State (T)	λ_{abs} nm	ε $M^{-1}cm^{-1}$	Φ_{PL} %	λ_f nm	Φ_f %	τ_f ns	τ_0^f ns	k_r^f s^{-1}	k_{nr}^f s^{-1}	λ_p nm	Φ_p %	τ_p s	τ_0^p s	k_r^p s^{-1}	k_{nr}^p s^{-1}
**1**	Solution (RT)[a]	299	14500	3.5	354	3.5	1.3	37.1	$3*10^7$	$7*10^8$	nd[b]		nd			
	Crystalline (RT)[c]			3.7	369	3.4	1.6	47.1	$2*10^7$	$6*10^8$	524	0.3	0.68 (79%)[d]	107	0.01	3.1
	Crystalline (77 K)	-		-	368		-	-			471, 502, 541		2.27			
	Frozen glass (77 K)[e]	-		-	349						404, 427		1.45			
**2**	Solution (RT)[a]	306	15600	6.6	359	6.6	2.0	30.0	$3*10^7$	$5*10^8$	nd		nd			
	Crystalline (RT)[c]	-		6.9	369	6.9	1.6	23.2	$4*10^7$	$6*10^8$	nd		nd			
	Crystalline (77 K)			-	352, 366		-	-			426, 449		1.22 (61%)[d]			
	Frozen glass (77 K)[e]	-		-	373		-	-			417, 442		1.57			
**3**	Solution (RT)[a]	318	15800	6.3	361	6.3	1.7	30.0	$3*10^7$	$6*10^8$	nd		nd			
	Crystalline (RT) c	-		18.2	371, 390	17.0	1.4	8.2	$1*10^8$	$6*10^8$	540, 575	1.2	0.47	16	0.06	5.1
	Crystalline (77 K)				372, 392, 415		1.4				488, 538, 582, 630		1.54			
	Frozen glass (77 K)[e]	-		-	375		-	-			421, 446		1.48			
	PMMA Film (RT)[f]			-	365		-	-								
	PMMA Film (77 K)			-	367		1.6	-			422, 447		1.57			
**4**	Solution (RT)[a]	324	16800	8.0	368	8.0	1.5	18.8	$5*10^7$	$6*10^8$	nd		nd			
	Crystalline (RT)[c]	-		6.3	381	6.3	1.5	23.8	$4*10^7$	$6*10^8$	nd		nd			
	Crystalline (77 K)	-		-	370			-			428,465		1.32 (68%)[d]			
	Frozen glass (77 K)[e]	-		-	374		-	-			425, 452		1.36			

[a] Measured in hexane at RT; [b] not detected (nd); [c] measured in the crystalline state at RT; [d] major component of lifetimes; [e] measured in methylcyclohexane at 77 K; the phosphorescence maxima of different vibrational levels have the same lifetimes; [f] measured in a PMMA (poly(methyl methacrylate) film at room temperature.

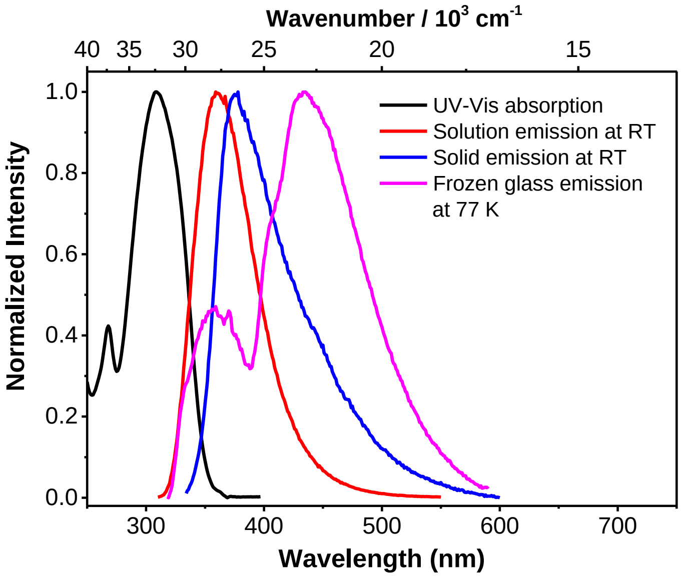

Figure S14. Normalized absorption and emission spectra of tris(2-methylphenyl)borane (E_x = 305 nm) in hexane.

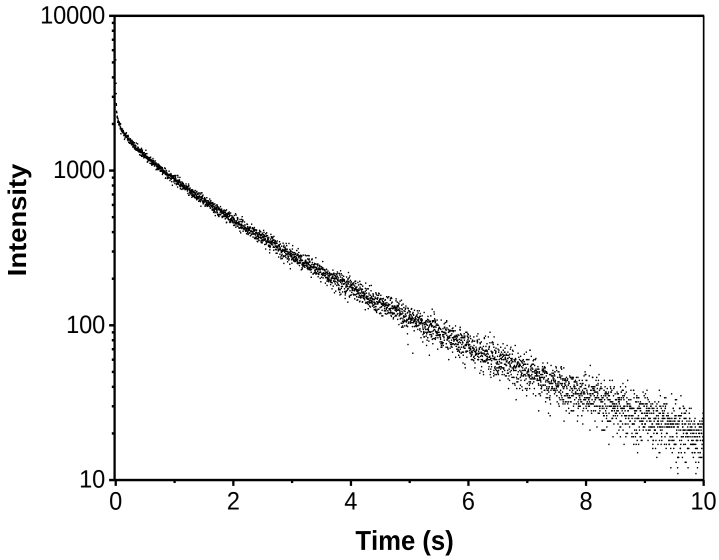

Figure S15. Decay of the 450 nm emission from tris(2-methylphenyl)borane in a frozen methylcyclohexane glass at 77 K.

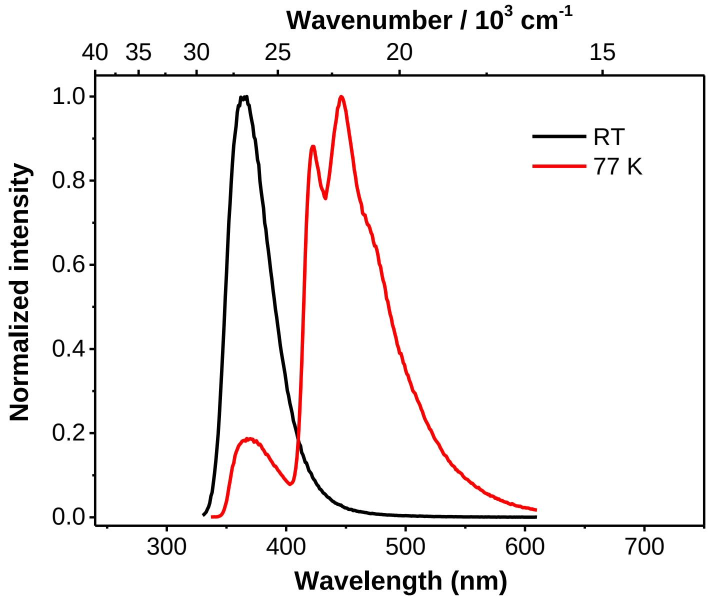

Figure S16. Normalized emission spectra of **3** in a PMMA film (1 %) at room temperature and 77 K (E_x = 305 nm).

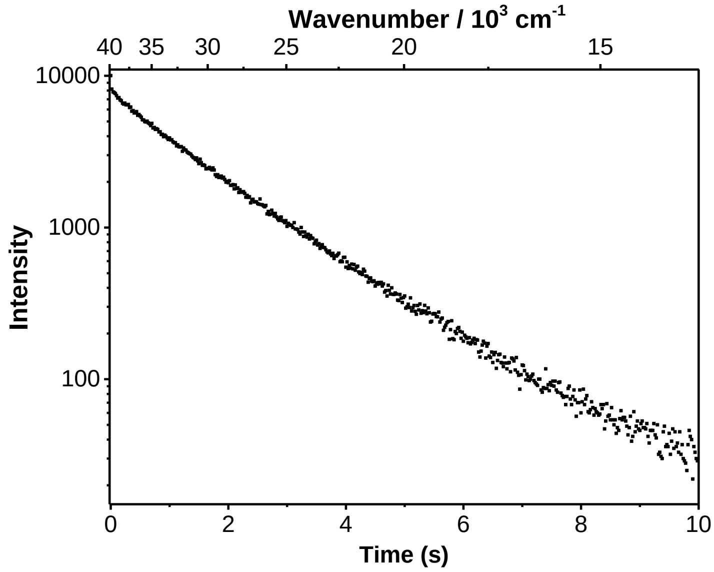

Figure S17. Decay of the 450 nm emission from **3** in a PMMA film (1 %) at 77 K.

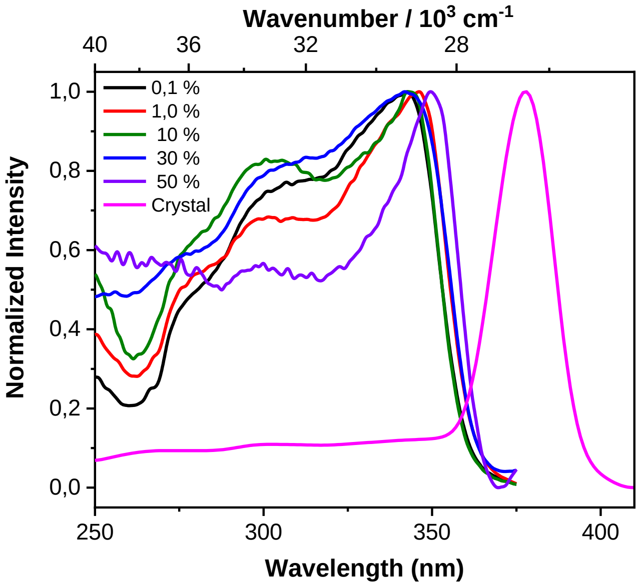

Figure S18. Normalized excitation spectra of compound **3** in 0.1, 1.0, 10, 30 and 50 % PMMA film and the crystalline state.

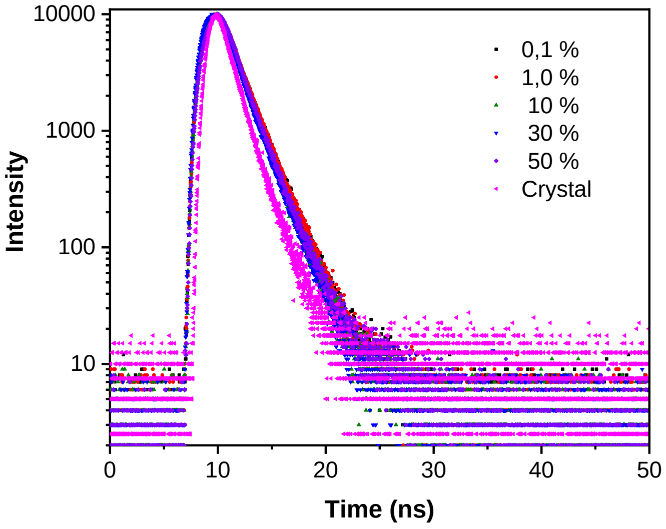

Figure S19. Decays of fluorescence at maximum emission wavelength of compound **3** in 0.1, 1.0, 10, 30 and 50 % PMMA film and the crystalline state.

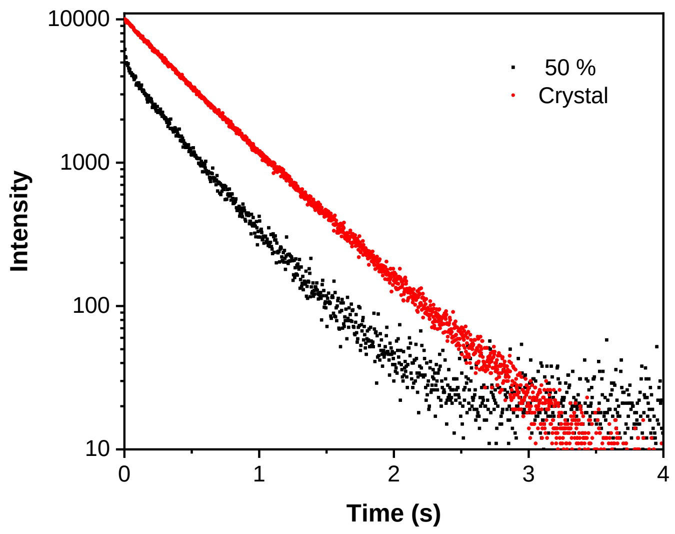

Figure S20. Decays of phosphorescence emission at 550 nm of compound **3** in 50 % PMMA film (407 ms) and the crystalline state (478 ms) at room temperature in air.

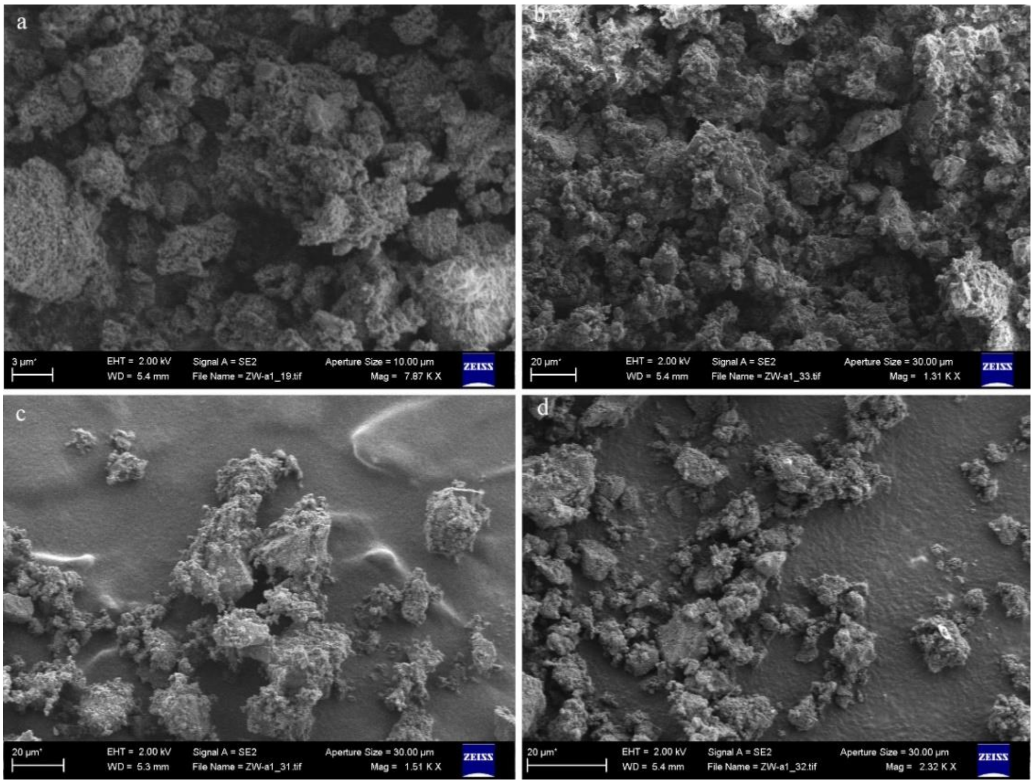

Figure S21. (a)-(d) SEM pictures of **3** in ball-milled powder state.

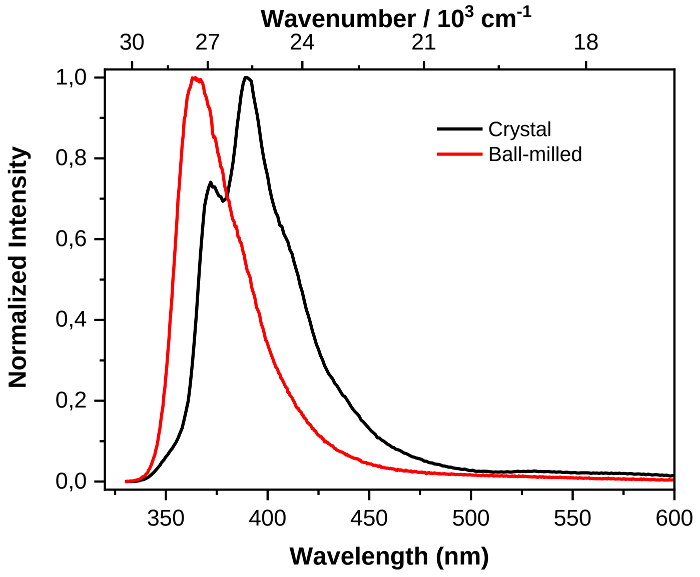

Figure S22. Normalized PL emission spectra of **3** in the crystalline state and ball-milled powder state at room temperature (E_x = 305 nm).

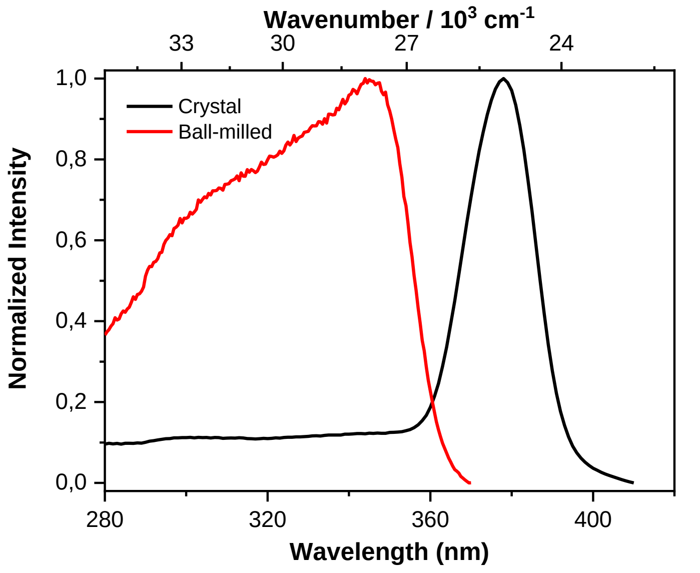

Figure S23. Normalized excitation spectra of **3** in the crystalline state and ball-milled powder state at room temperature.

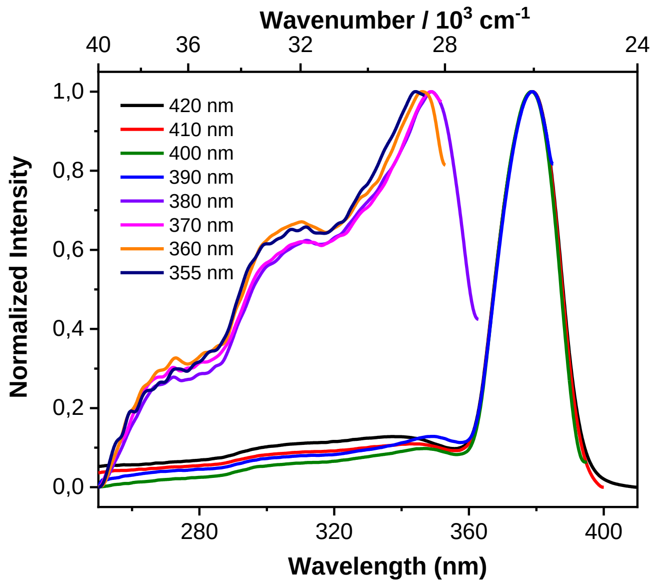

Figure S24. Normalized emission dependent excitation spectra of compound **3** in the crystalline state from 420 to 355 nm.

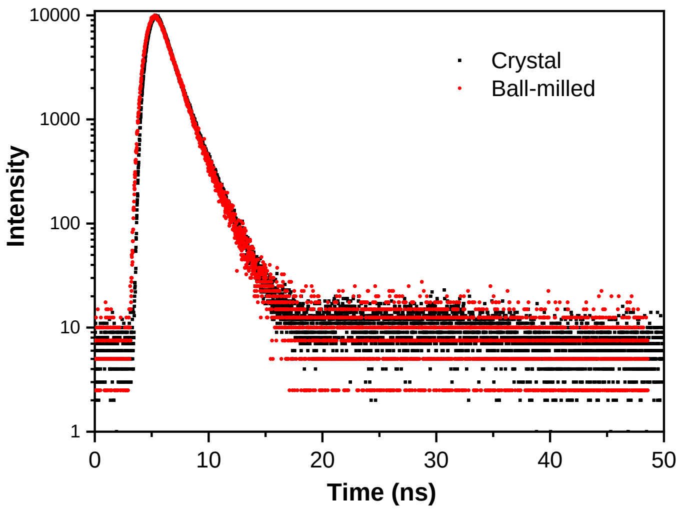

Figure S25. Fluorescence decay of **3** in the crystalline state and ball-milled powder state at room temperature.

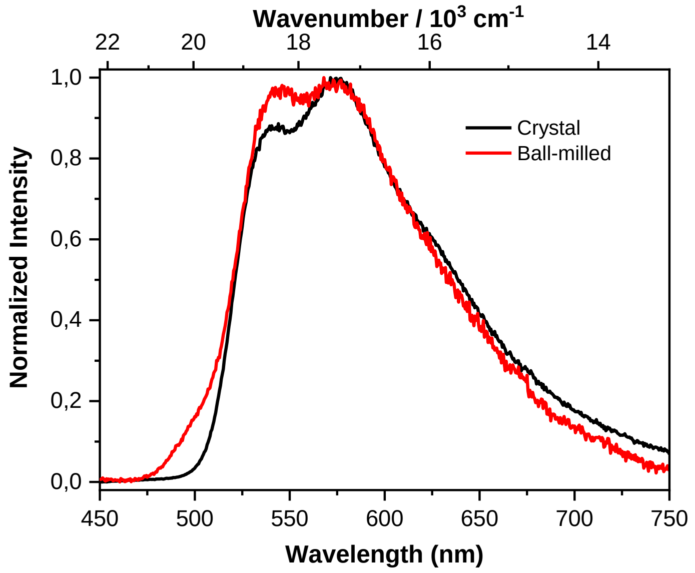

Figure S26. Normalized time-gated phosphorescence emission spectra of **3** in the crystalline state and ball-milled powder state at room temperature (E_x = 305 nm).

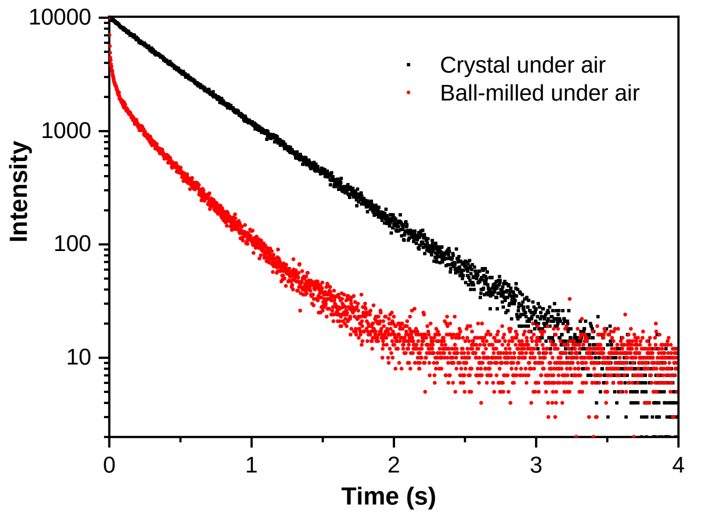

Figure S27. Phosphorescence decay of **3** in the crystalline state (470 ms) and ball-milled powder state (340 ms) at room temperature under air.

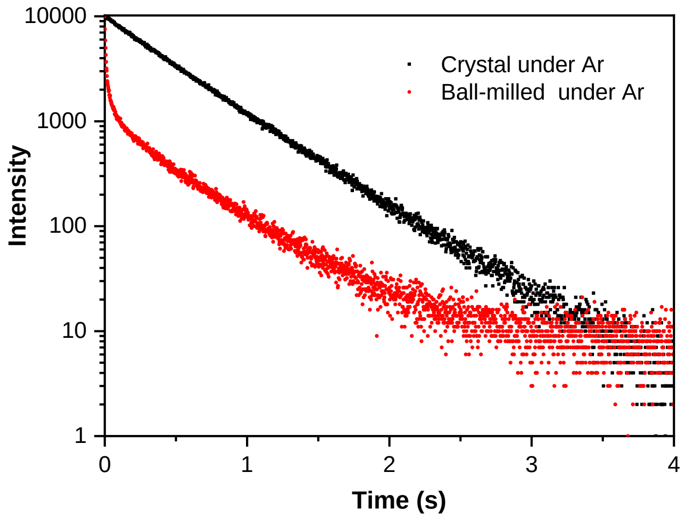

Figure S28. Phosphorescence decay of **3** in the crystalline state (478 ms) and ball-milled powder state (481 ms) at room temperature under argon.

Powder X-ray diffraction

Table S5. Unit cell parameters of **3** obtained from the LeBail refinement of powder X-ray diffraction data at room temperature. Ball-milled samples were obtained by placing compound **3** in a stainless steel vial (2 mL) and grinding with 5 steel balls (3 mm diameter) for 5 minutes at a frequency of 15 Hz in a Lab Wizz LMLW 320/2 ball mill.

**Data**	**# manually ground** (sample **A**)	**# ball-milled** (sample **B**)
λ / Å, radiation	1.5406, Cu-Kα_1	1.5406, Cu-Kα_1
θ range / °	5 – 60	5 – 60
a / Å	8.6841(9)	8.6437(9)
b / Å	9.7658(9)	9.7390(9)
c / Å	12.3669(8)	12.3531(14)
α / °	98.548(7)	98.497(8)
β / °	90.992(7)	90.908(7)
γ / °	107.646(7)	107.648(5)
Volume / Å^3	986.19(10)	978.0(2)
R_p	0.0525	0.0264
wR_p	0.1519	0.0387

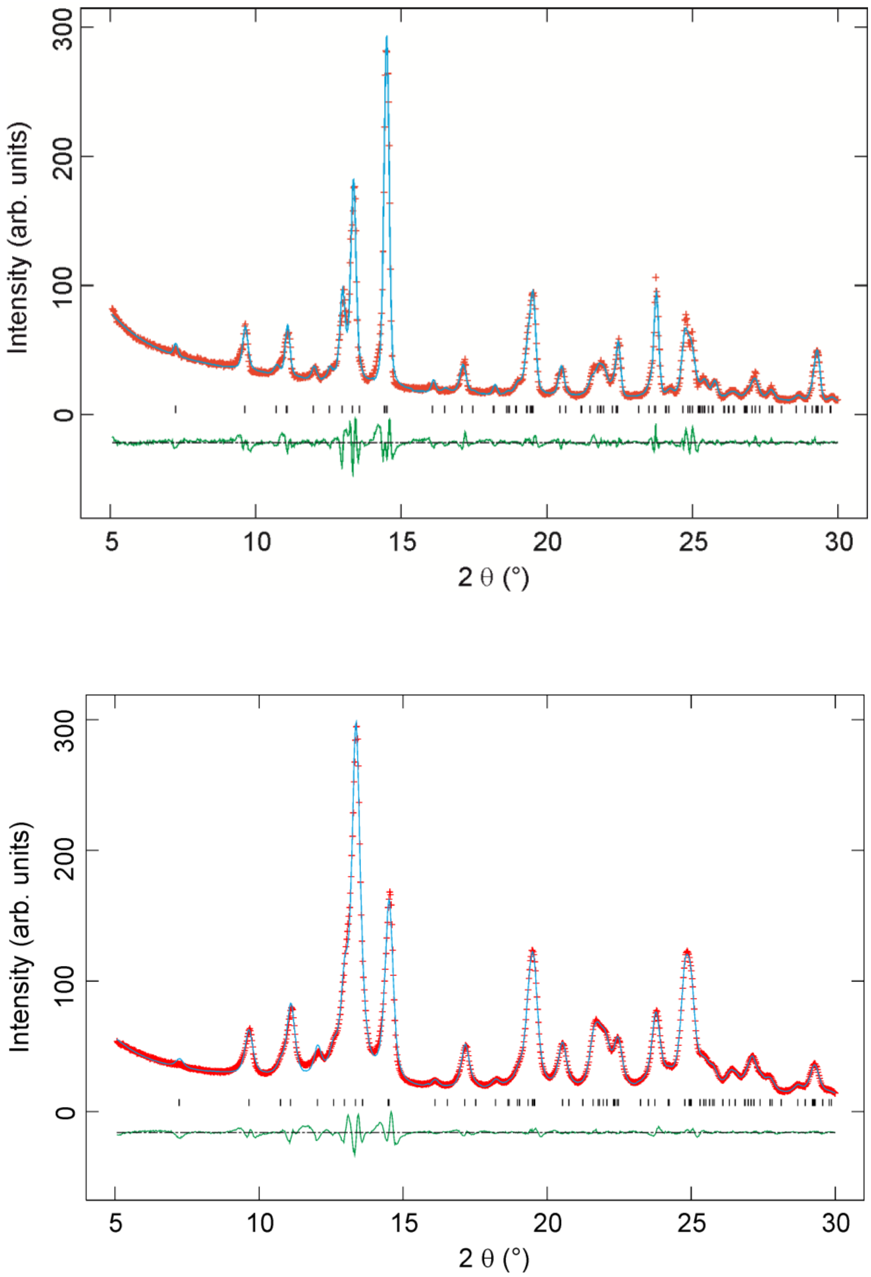

Figure S29. Powder X-ray diffraction patterns of compound **3** after grinding crystals in an agate mortar (sample **A**, top) and after ball-milling (sample **B**, bottom) in the selected range from 5 – 30° 2θ. Bottom: The increased reflection width is due to a smaller particle size after ball milling. Red crosses represent the experimental values. The continuous blue line shows the results of the Le Bail fit to the data. The difference between experimental data and LeBail fit is represented by the green line at the bottom of the plot. Vertical bars show the positions of the Bragg reflections of compound **3**.

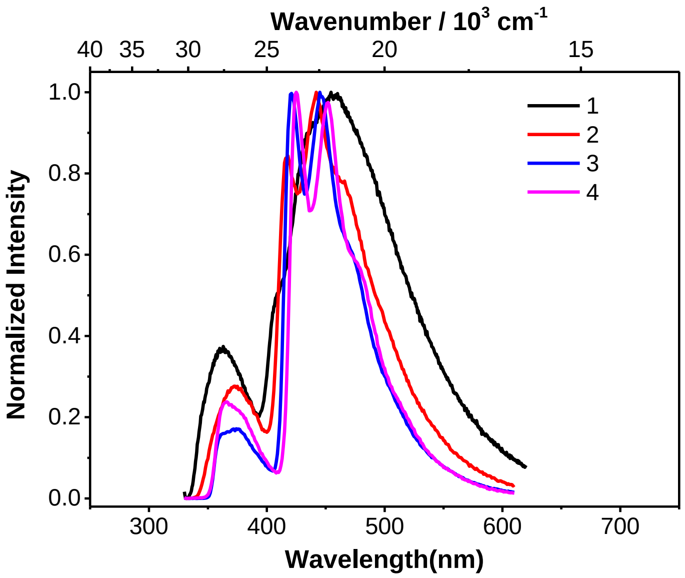

Figure S30. Normalized photoluminescence emission spectra of **1-4** in a frozen methylcyclohexane glass at 77 K (E_x = 305 nm).

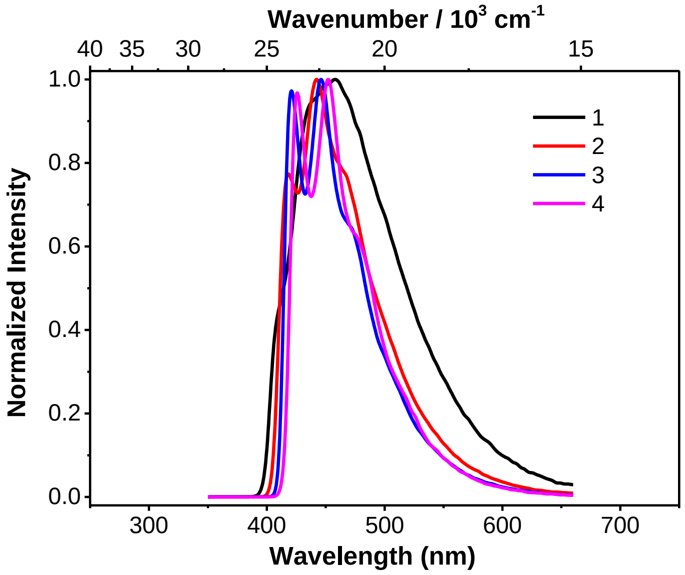

Figure S31. Normalized time-gated phosphorescence emission spectra of compounds **1-4** in a frozen methylcyclohexane glass at 77 K (E_x = 305 nm).

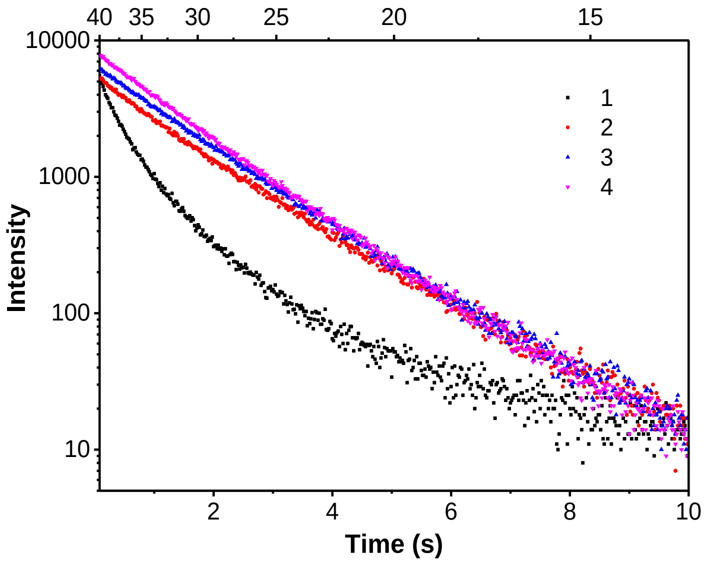

Figure S32. Decays of the phosphorescent emissions of **1-4** in a frozen methylcyclohexane glass at 77 K.

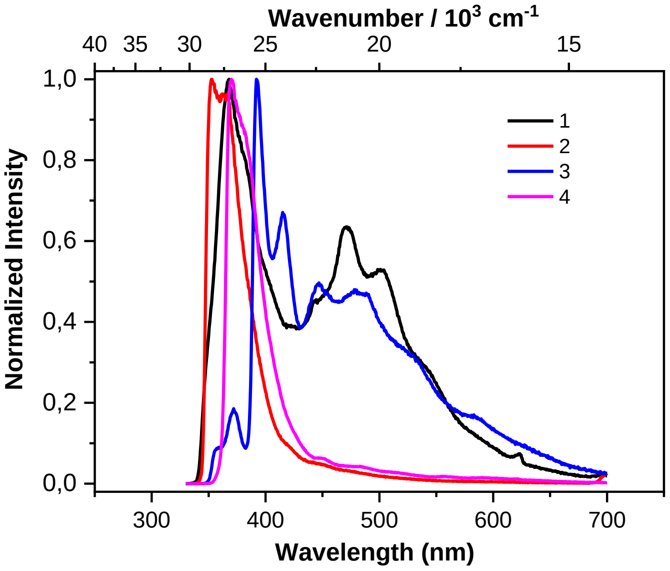

Figure S33. Normalized photoluminescent emission spectra of crystalline **1-4** at 77 K (E_x = 305 nm).

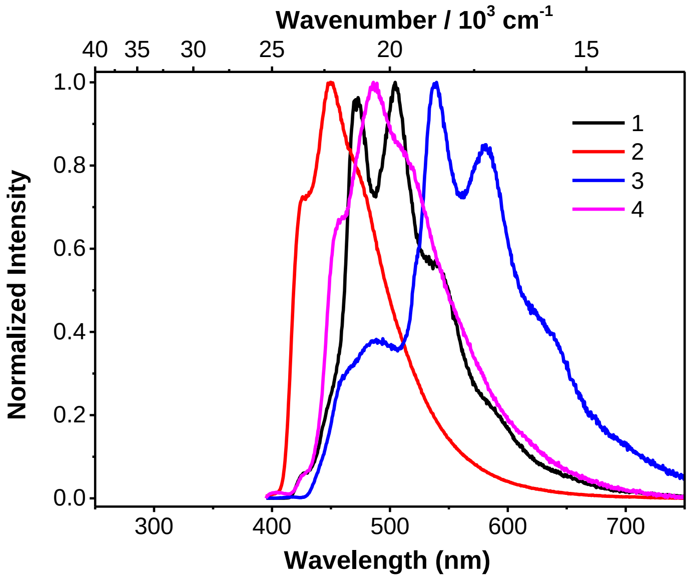

Figure S34. Normalized time-gated phosphorescence emission spectra of crystalline **1-4** at 77 K (E_x = 305 nm).

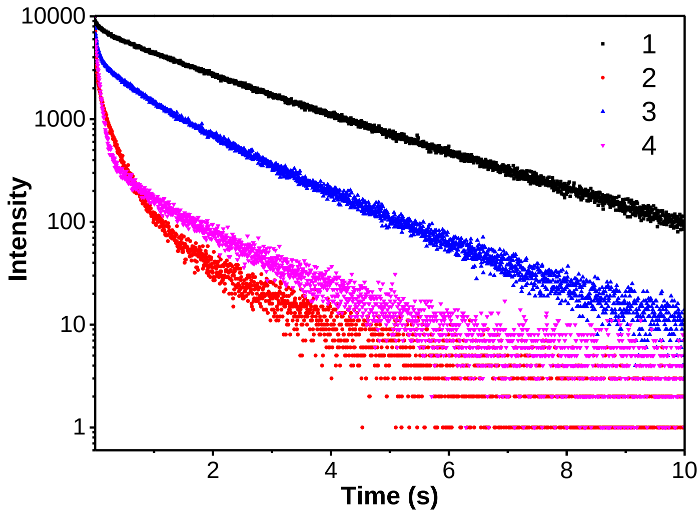

Figure S35. Decays of the maximum phosphorescent emissions of crystalline **1-4** at 77 K.

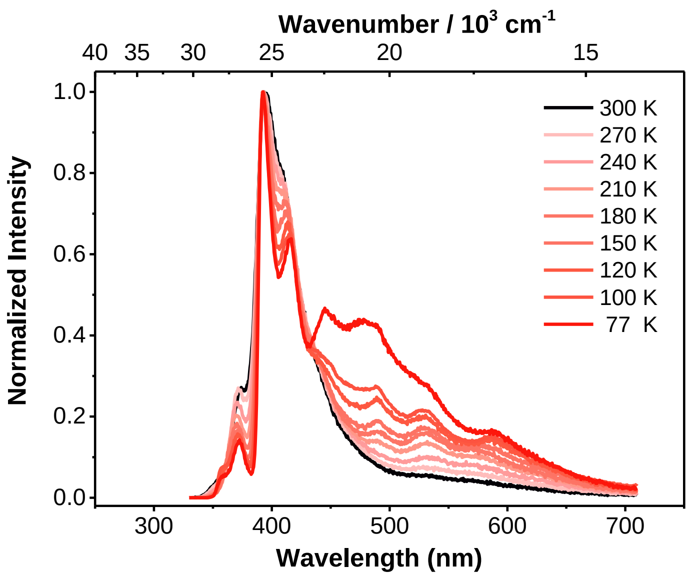

Figure S36. Normalized photoluminescent emission spectra of crystalline **3** at different temperatures (E_x = 305 nm).

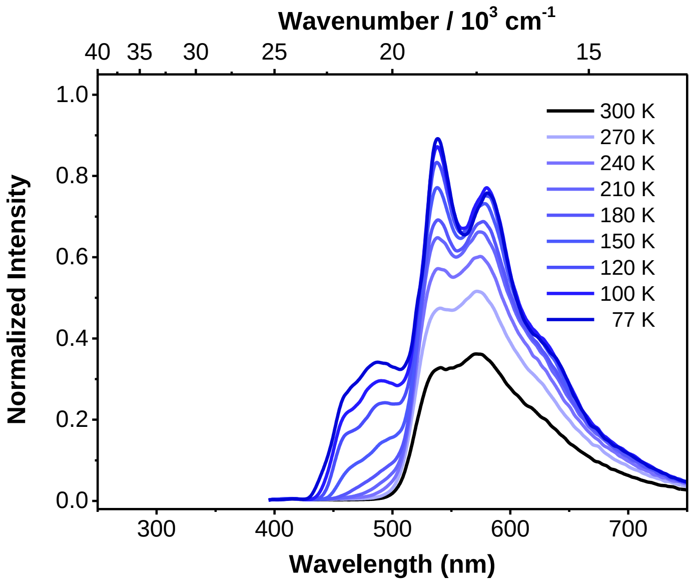

Figure S37. Normalized time-gated phosphorescence emission spectra of crystalline **3** at different temperatures (E_x = 305 nm).

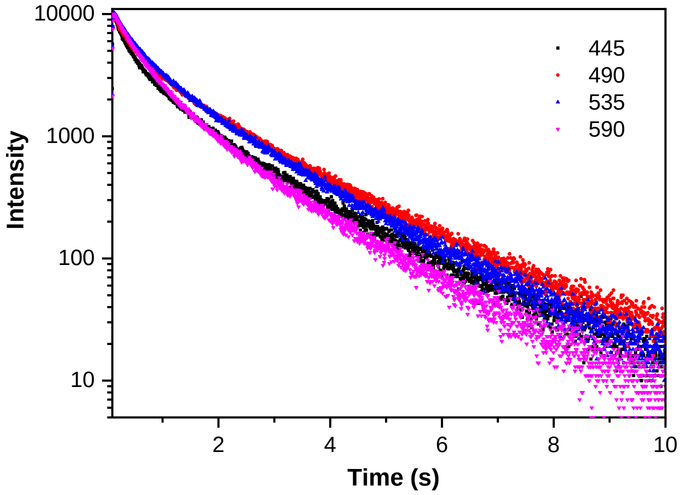

Figure S38. Decays of the phosphorescent emission of crystalline **3** at 445, 490, 535 and 590 nm at 77 K.

V. Single-crystal X-ray diffraction

Table S6. Single-crystal X-ray diffraction data and structure refinements for **1**, **2**, **3**, and **4**.

Data	**1**	**2**	**3**	**4**
CCDC number	1940099	1940100	1940101	1940107
Empirical formula	$C_{22}H_{23}B$	$C_{23}H_{25}B$	$C_{24}H_{27}B$	$C_{25}H_{29}B$
Formula weight / g·mol^{-1}	298.21	312.24	326.26	340.29
T / K	100(2)	100(2)	100(2)	100(2)
radiation, λ / Å	MoKα 0.71073	MoKα 0.71073	MoKα 0.71073	MoKα 0.71073
Crystal size / mm³	0.23×0.29×0.78	0.28×0.35×0.41	0.23×0.36×0.41	0.12×0.42×0.48
Crystal color, habit	colorless block	colorless block	colorless block	colorless plate
μ / mm^{-1}	0.063	0.064	0.063	0.063
Crystal system	Monoclinic	Monoclinic	Triclinic	Triclinic
Space group	$P2_1/c$	$P2_1/n$	$P\bar{1}$	$P\bar{1}$
a / Å	8.148(6)	8.138(5)	8.511(4)	8.445(3)
b / Å	10.828(3)	18.060(10)	9.675(2)	9.647(3)
c / Å	19.832(9)	12.161(7)	12.241(4)	12.890(5)
α / °	90	90	99.28(2)	99.166(15)
β / °	97.06(4)	90.293(11)	90.87(4)	91.49(3)
γ / °	90	90	107.44(2)	105.371(15)
Volume / Å^3	1736.5(16)	1787.4(17)	946.9(6)	997.1(6)
Z	4	4	2	2
ρ_{calc} / g·cm^{-3}	1.141	1.160	1.144	1.133
$F(000)$	640	672	352	368
θ range / °	2.069 - 28.310	2.019 - 28.338	2.514 - 26.732	1.604 - 28.441
Reflections collected	19166	19909	16834	24532
Unique reflections	4300	4440	4009	5019
Parameters / restraints	212 / 0	222 / 0	232 / 0	253 / 0
GooF on F^2	1.054	1.029	1.035	1.050
R_1 [I>2σ(I)]	0.0469	0.0501	0.0437	0.0480
wR^2 (all data)	0.1256	0.1350	0.1174	0.1316
Max. / min. residual electron density / e·Å^{-3}	0.364 / –0.236	0.382 / –0.207	0.365 / –0.230	0.366 / –0.244

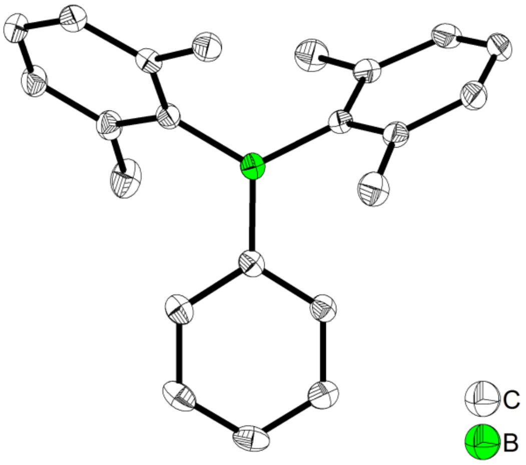

Figure S39. The solid-state molecular structure of **1** determined by single-crystal X-ray diffraction at 100 K. All ellipsoids are drawn at the 50% probability level, and H atoms are omitted for clarity.

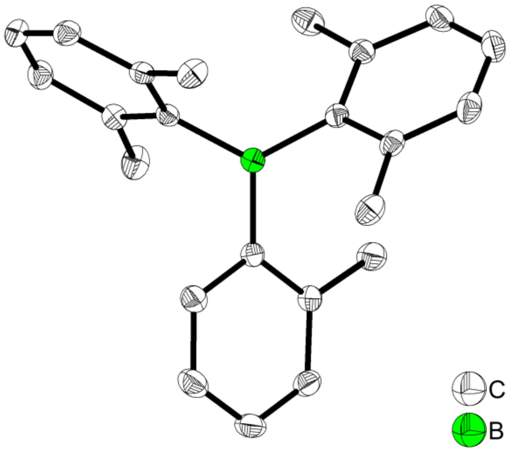

Figure S40. The solid-state molecular structure of **2** determined by single-crystal X-ray diffraction at 100 K. All ellipsoids are drawn at the 50% probability level, and H atoms are omitted for clarity.

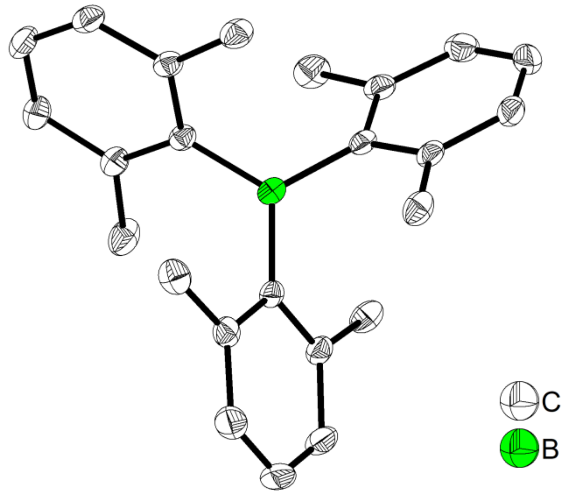

Figure S41. The solid-state molecular structure of **3** determined by single-crystal X-ray diffraction at 100 K. All ellipsoids are drawn at the 50% probability level, and H atoms are omitted for clarity.

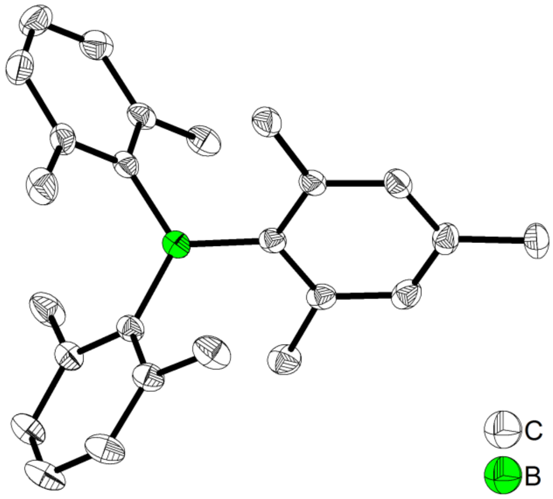

Figure S42. The solid-state molecular structure of **4** determined by single-crystal X-ray diffraction at 100 K. All ellipsoids are drawn at the 50% probability level, and H atoms are omitted for clarity. The major structural conformation with a refined occupancy of 0.809(4) is shown here.

Hirshfeld surface analysis and intermolecular interactions in detail

In order to compare and classify the types and magnitudes of the intermolecular interactions within single crystals of these four triarylboranes, which organize in a complex three-dimensional arrangement, the concept of Hirshfeld surface analysis was applied.[23] The molecules are most densely packed in compound **2**, as is clear from both the crystal packing coefficient c_k, which corresponds to the ratio of volume occupied by all molecules in the unit cell to the unit cell volume, and the surface of the crystal's void per formula unit, which is obtained from the Hirshfeld analysis (Table S8).[24] In order to quantify the nature and type of intermolecular interactions from the Hirshfeld surface analysis in a two-dimensional, graphical way, fingerprint plots and their breakdown to the individual contributions can be used.[25] This separation of relative contributions in crystals of **1**-**4** exhibited a strong contribution of H···H interactions (75 – 83 %), followed by a significant amount of C···H interactions (17 – 25 %) (Figures S43-S44). Only a very weak contribution of C···C interactions is observed for compound **3** (0.2 %). The similarity of the amounts and types of interactions in the four compounds is demonstrated in the two-dimensional fingerprint plots, which plot the distances from points on the surface to the nearest nuclei outside the surface, d_e, as a function of the distance to the nearest nucleus inside the surface, d_i.[23c] The largest differences in these plots are observed for compound **1**, which has close H···H contacts as demonstrated by the spike in the bottom left corner, and for compound **3**, which has a small percentage of C···C interactions. While this analysis shows the relative contributions of the different types of intermolecular interactions, we are now interested in their strengths in the individual crystal structures. Compound **1** exhibits several significant intermolecular interactions. Two strong C−H···C interactions (C···H = 2.835 and 2.839 Å, C−H···C = 168 and 166°) exist, three of intermediate strength (C−H···C = 138 – 160°), and one weak one (C−H···C = 127°). In addition, there is a short H···H contact (2.241 Å) between two aryl rings (Table S9). Compound **2**, although more densely packed than **1**, shows significantly fewer and weaker intermolecular interactions, i.e. three C−H···C interactions of intermediate strength (C−H···C = 145 – 155°), and one weak one (C−H···C = 114°). It is the only compound that has a nearly linear, weak C−H···π interaction towards the centroid of an m-xylyl ring with H···π = 2.907 Å. In addition, two close C···C contacts (C···C = 3.334 and 3.384 Å) are present, a strong one between two aryl rings, and a weak one

between the same aryl and a methyl group (Table S9). Compound **3** shows three linear intermolecular C−H···C interactions with C−H···C angles ranging from 164 to 172° which are strong, intermediate and weak according to their C···H distances of 2.841, 2.871, and 2.897 Å, respectively. This is quite similar to compound **1**. However, in addition to the C−H···C interactions, compound **3** also has a strong C···C interaction (C6···C6 = 3.319 Å) between two aryl rings with an approximately parallel alignment of their planes. This is the shortest nearest-neighbor (nn) C···C distance in all of the compounds. The interplanar separation between the aryl planes is only 2.980 Å; however, the offset shift is large (4.221 Å), resulting in a centroid-to-centroid distance of 5.167 Å, the latter two values being too large for a typical offset face-to-face π···π stacking interaction between two arenes (Table S10), which typically have values ranging from 3.3–3.8 Å for the interplanar separation, < 4.0 Å for the offset, and < 5.0 Å for the centroid-to-centroid distance.[26] There exists another arrangement of nearly parallel aryl rings, which has a longer C···C distance (3.495 Å) and interplanar separation (3.397 Å), but a smaller shift (3.493 Å) and, hence, a smaller centroid-to-centroid distance of 4.872 Å, all of those values being within the typical range of weak π···π interactions. The aryl rings, and hence the π···π interaction, are situated close to the voids, which are around the origin (Figure 5). A C···C offset aryl-aryl interaction is also present in compounds **2** and **4**; however, the C−H···C interactions are much weaker in these compounds. In crystals of compound **4**, intermolecular interactions are the weakest. This is in agreement with the loosest packing mode. There is a relatively close H···H contact (2.311 Å) between two methyl groups, one weak C−H···C interaction, and the weak C···C offset aryl-aryl interaction (Table S9).

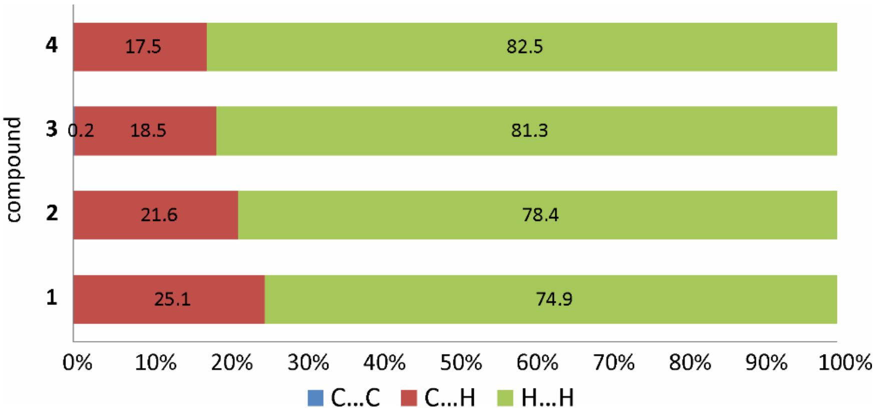

Figure S43. Percentage contributions to the Hirshfeld surface area for the various close intermolecular contacts in compounds **1**, **2**, **3**, and **4** at 100 K.

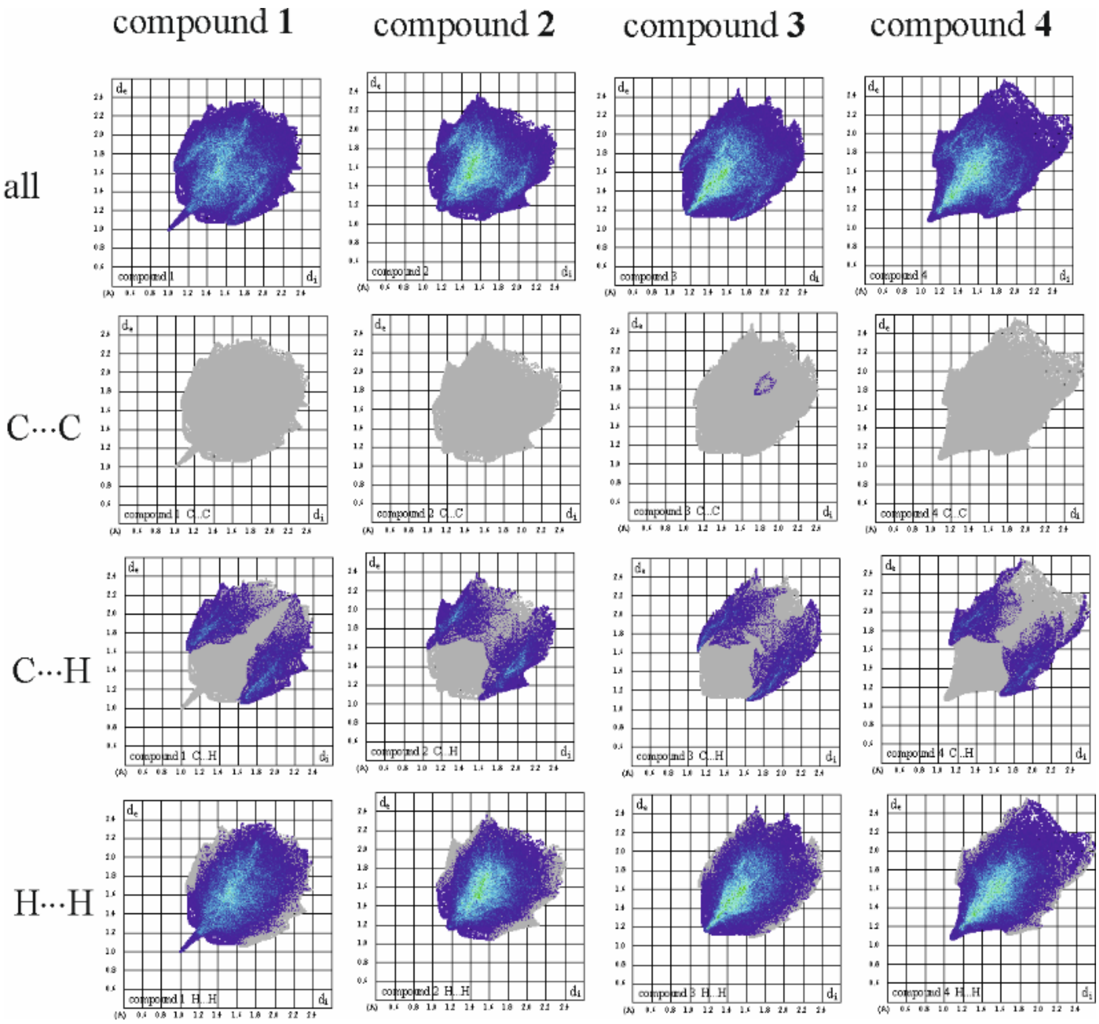

Figure S44. Two-dimensional fingerprint plots of molecules **1**, **2**, **3**, and **4** calculated from the Hirshfeld surfaces. The top row shows the complete fingerprint plots, while the other plots indicate the contributions of the individual intermolecular interactions (C···C, C···H, and H···H from top to bottom) within the grey area of all contributions.

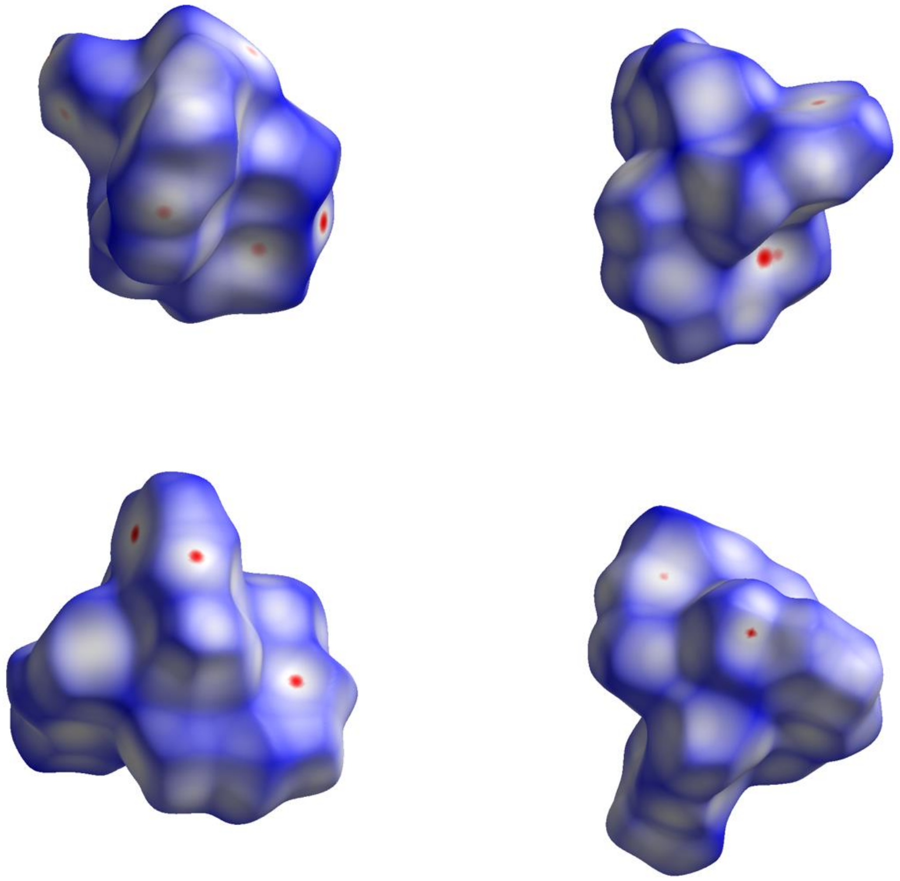

Figure S45. Hirshfeld surfaces of compounds **1** (top left), **2** (top right), **3** (bottom left), and **4** (bottom right) at 100 K mapped with d_{norm} over the range -0.16 to 1.40. Close contacts are shown red on the surface.

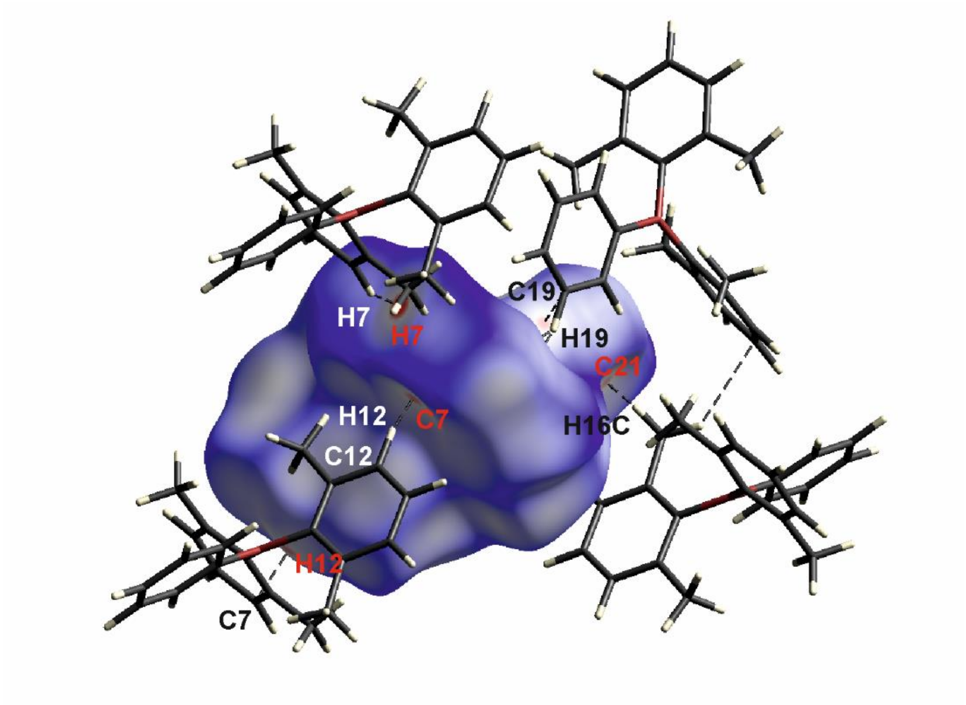

Figure S46. Hirshfeld surface of compound **1** mapped with d_{norm} over the range -0.16 to 1.40 at 100 K. Neighbouring molecules associated with close contacts are shown.

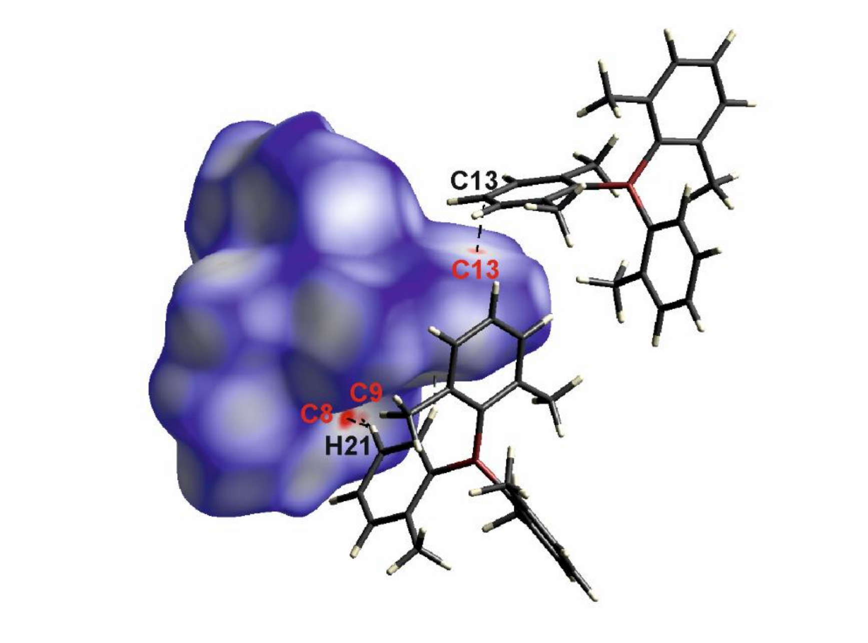

Figure S47. Hirshfeld surface of compound **2** mapped with d_{norm} over the range -0.16 to 1.40 at 100 K. Neighbouring molecules associated with close contacts are shown.

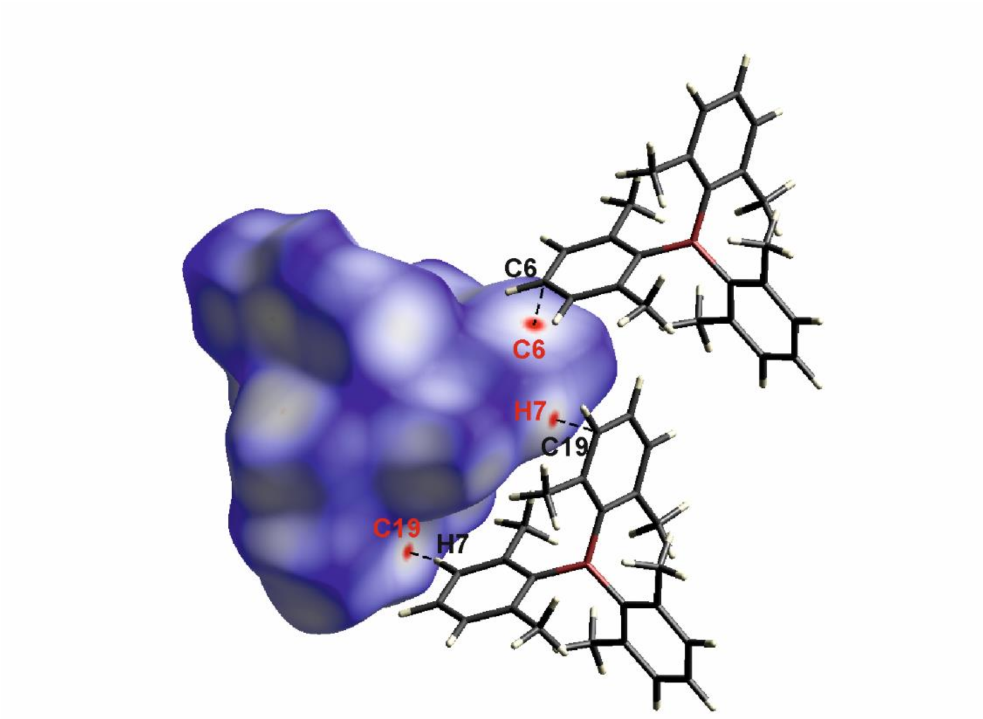

Figure S48. Hirshfeld surface of compound **3** mapped with d_{norm} over the range -0.16 to 1.40 at 100 K. Neighbouring molecules associated with close contacts are shown.

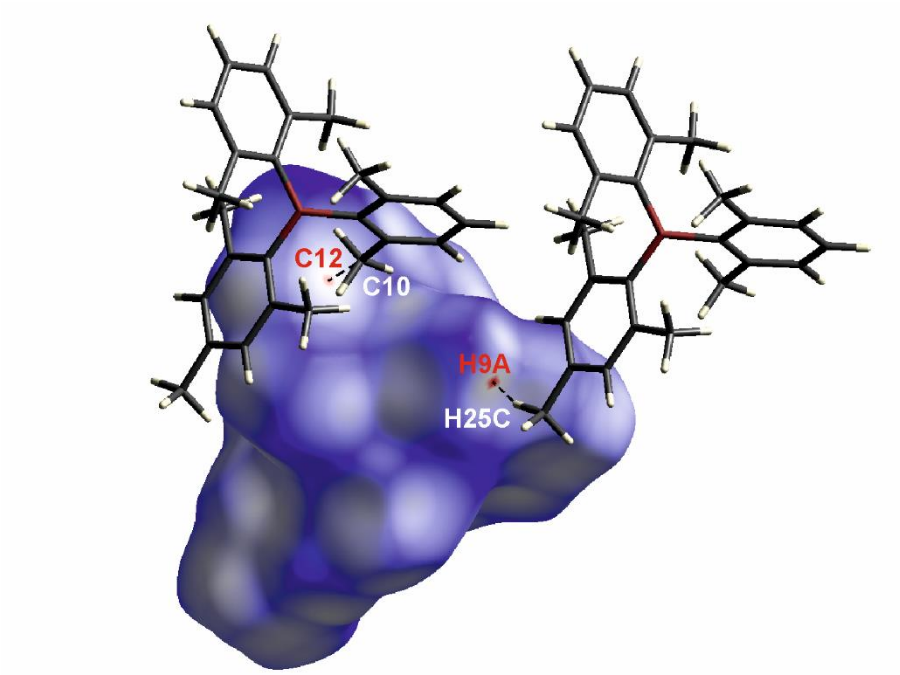

Figure S49. Hirshfeld surface of compound **4** mapped with d_{norm} over the range -0.16 to 1.40 at 100 K. Neighbouring molecules associated with close contacts are shown.

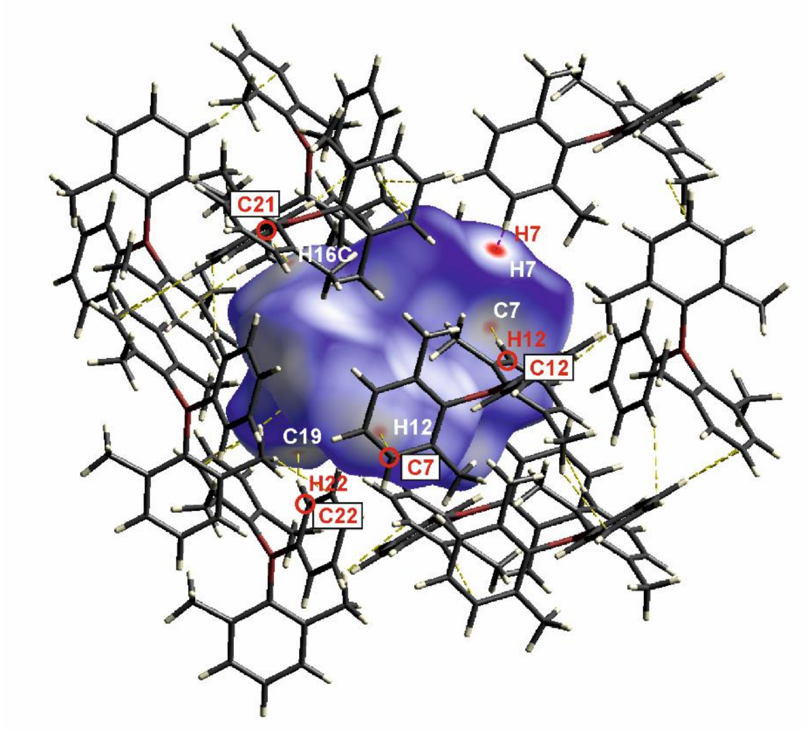

Figure S50. Hirshfeld surface of compound **1** mapped with d_{norm} over the range -0.16 to 1.40 at 100 K. Neighbouring molecules associated with close contacts are shown. Closest contacts are labelled.

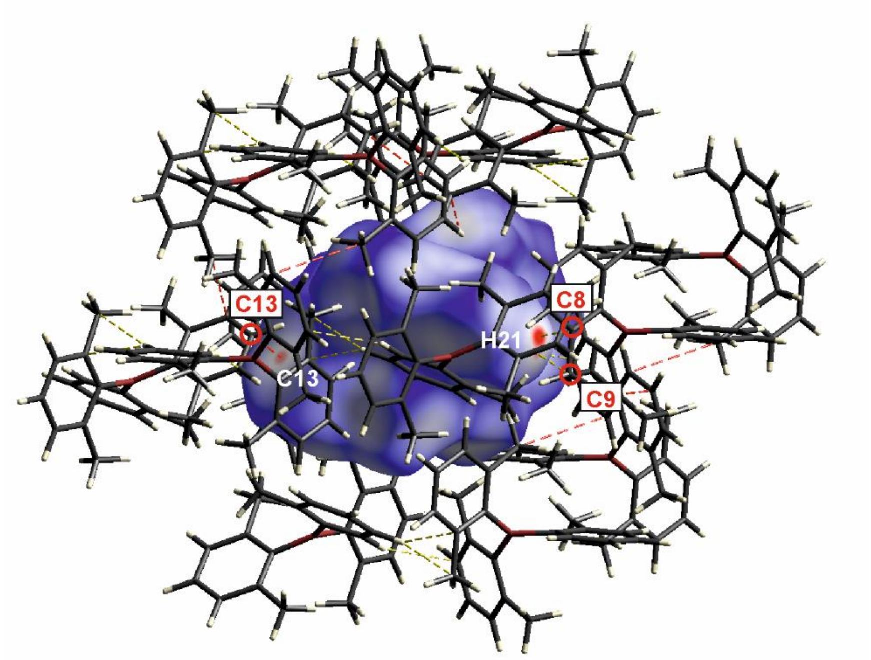

Figure S51. Hirshfeld surface of compound **2** mapped with d_{norm} over the range -0.16 to 1.40. Neighbouring molecules associated with close contacts are shown. Closest contacts are labelled.

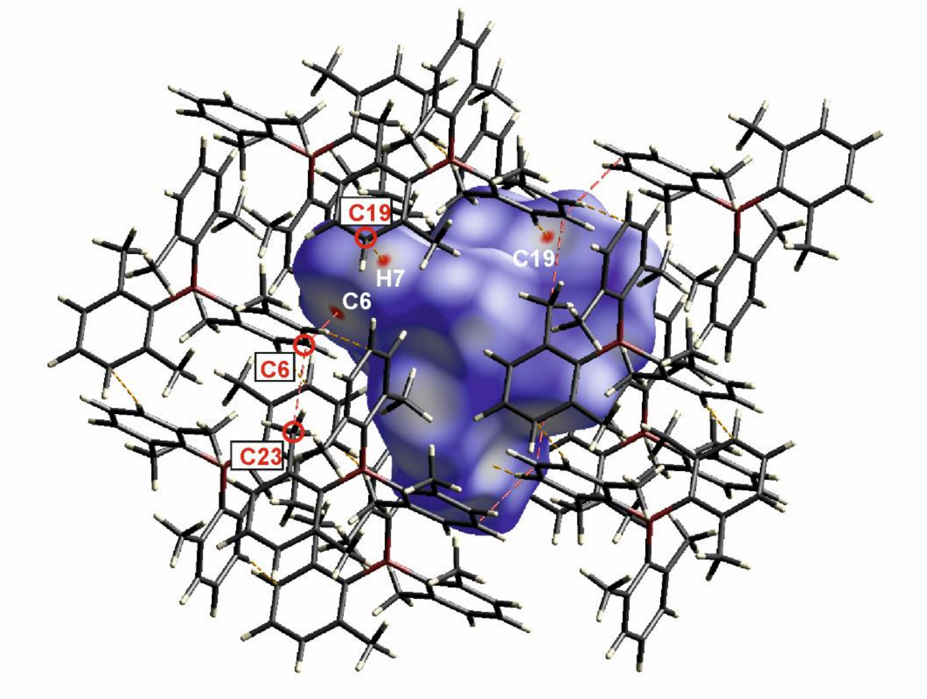

Figure S52. Hirshfeld surface of compound **3** mapped with d_{norm} over the range -0.16 to 1.40 at 100 K. Neighbouring molecules associated with close contacts are shown. Closest contacts are labelled.

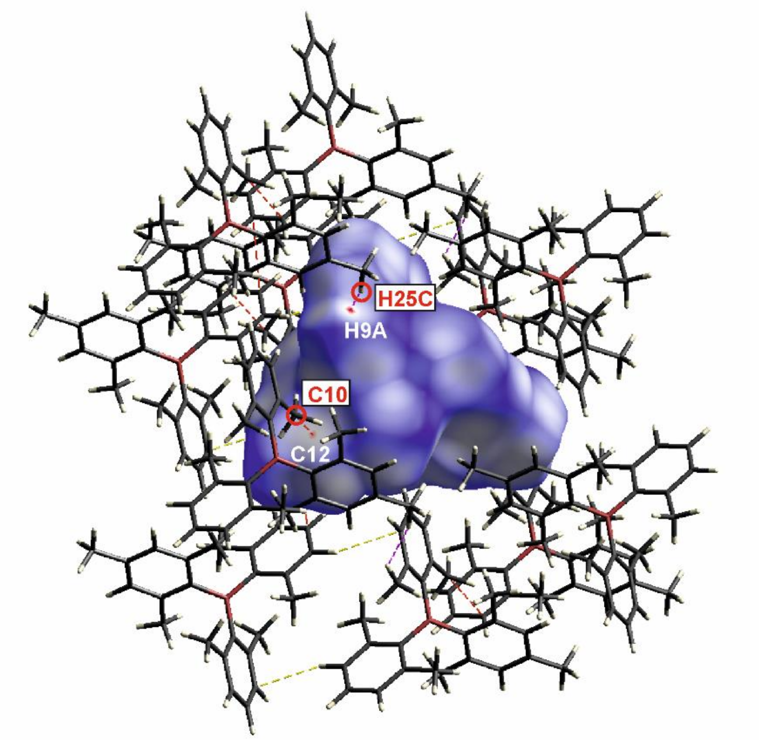

Figure S53. Hirshfeld surface of compound **4** mapped with d_{norm} over the range -0.16 to 1.40 at 100 K. Neighbouring molecules associated with close contacts are shown. Closest contacts are labelled.

Table S7. Selected bond lengths (Å) and angles (°) in compounds **1**, **2**, **3**, and **4** at 100 K**.**

	**1**	**2**	**3**	**4** [a]
Sum ∠ CBC	360.0(1)	359.9(1)	360.0(1)	360.0(1)
B–C	1.5867(18)<br>1.5819(18)<br>1.5686(19)	1.583(2)<br>1.581(2)<br>1.570(2)	1.5795(19)<br>1.5826(18)<br>1.5858(18)	1.5848(18)<br>1.5794(18)<br>1.5763(18)
∠ BC3 – aryl	65.28(6)<br>57.45(6)<br>16.09(9)	60.53(5)<br>56.67(6)<br>41.91(6)	53.52(7)<br>52.91(7)<br>53.92(6)	49.96(5)<br>54.85(6)<br>51.15(6)

[a] The mesityl group is disordered with one of the m-xylyl groups.

Table S8. Properties of crystals of compounds **1**, **2**, **3**, and **4** at 100 K: volume within van der Waals (V_m), Hirshfeld (V_H), and surface of the crystal voids (V_v), crystal packing coefficient (c_k), solvent accessible volume (V_{solv}), and percentage of intermolecular contacts.

	**1**	**2**	**3**	**4** [a]
V_m / Å^3	283.36	298.60	312.20	323.94
V_H / Å^3	427.11	440.18	466.68	491.63
V_v / Å^3	211.86	169.00	103.82	111.40
V_v / Å^3 per formula unit	52.97	42.25	51.91	55.70
c_k	0.654	0.668	0.659	0.650
V_{solv} / Å^3	0.0	0.0	9.0	0.0
C···C / %	0.0	0.0	0.2	0.0
C···H / %	25.1	21.6	18.5	17.5
H···H / %	74.9	78.4	81.3	82.5

[a] The mesityl group is disordered with one of the m-xylyl groups. The configuration with the major occupancy (80%) is considered in this comparison.

Table S9. Intermolecular C−H⋯C(π), C⋯C, and H⋯H interaction distances (Å) and angles (°) in compounds **1**, **2**, **3**, and **4** at 100 K. Distances and angles of strong interactions are in bold type.

Compound	C−H⋯C	H⋯C/H	C⋯C	∠(CHC)
1	H7⋯H7	**2.2413(12)**		
	C12−H12⋯C7	**2.8346(16)**	**3.770(2)**	**168.04(8)**
	C19−H19⋯C5	**2.8388(15)**	**3.767(2)**	**166.02(8)**
	C19−H19⋯C6	2.8866(19)	3.650(2)	138.19(8)
	C22−H22⋯C19	2.8555(15)	3.761(2)	159.93(8)
	C16(methyl)−H16C⋯C21	2.792(2)	3.634(3)	144.47(10)
	C16(methyl)−H16B⋯C13	2.8509(16)	3.532(2)	127.27(9)
2	C21−H21⋯C8	2.7521(17)	3.572(3)	144.95(10)
	C21−H21⋯C9(methyl)	2.859(2)	3.742(3)	155.05(10)
	C22−H22⋯C12	2.877(2)	3.756(3)	154.27(10)
	C10(methyl)−H10B⋯C14	2.8608(19)	3.385(2)	114.33(9)
	C12-H12⋯**π** (centroid of aryl#1)	2.9068(16)	3.830(3)	168.10(7)
	C13⋯C13		3.334(3)	
	C10(methyl)⋯C14		3.385(2)	
3	C7−H7⋯C19	**2.8407(17)**	**3.765(2)**	**164.41(9)**
	C14−H14⋯C21	2.8714(17)	3.815(2)	**171.96(9)**
	C5−H5⋯C12	2.897(2)	3.832(3)	168.18(9)
	C6⋯C6		**3.319(2)**	
	C6⋯C23(methyl)		3.392(2)	
4	H9A(methyl)⋯H25C (methyl)	2.3105(7)		
	C14−H14⋯C7	2.8931(18)	3.745(3)	149.84(9)
	C13A⋯C13A		3.392(2)	
	C10(methyl)⋯C12		3.3785(19)	

Table S10. Aryl···aryl (π···π) distances (Å) and angles (°) in crystals of **1**, **2**, **3**, and **4** at 100 K: nearest-neighbor (nn) C···C distances, centroid-centroid distances, interplanar separations, shifts, and slip angles. Aryl rings are numbered 1, 2, or 3 according to the C1, C2, or C3 atom being present and bonding to the boron atom, respectively. The closest interactions are in bold type.

Compound	Aryl···Aryl	nn C···C	Centroid-centroid distance	Interplanar separation	Shift	Slip angle
**1**	#1···#1	3.857(5)	6.123(3)	2.513(6)	5.583(4)	65.7
	#2···#2	4.315(5)	6.275(3)	3.507(5)	5.203(5)	56.0
	#1···#2	4.526(4)	6.587(3)	3.886(5) / 2.982(6)	5.319(5) / 5.874(4)	53.8/ 63.1
**2**	#2···#2	**3.3338(17)**	**5.000(2)**	**3.1607(13)**	**3.8745(19)**	**50.8**
	#3···#3	3.5833(12)	5.489(2)	3.0270(15)	4.578(3)	56.5
	#1···#1	4.1070(16)	6.348(3)	3.0521(15)	5.566(3)	61.3
**3**	#1···#1	**3.319(3)**	**5.1671(17)**	**2.980(3)**	**4.221(3)**	**54.8**
	#2···#2	**3.495(3)**	**4.8718(16)**	**3.397(3)**	**3.493(3)**	**45.8**
**4**	#2···#2	**3.392(3)**	**5.1873(18)**	**3.096(3)**	**4.162(2)**	**53.4**
	#3···#3	3.768(3)	5.2472(18)	3.550(3)	3.864(3)	47.4
	#2···#2	4.396(3)	6.077(2)	3.613(3)	4.887(3)	53.5

Table S11. Temperature-dependent single-crystal X-ray diffraction data and structure refinements of **3**, $C_{24}H_{27}B$. Formula weight = 326.26 g·mol^{-1}; F(000) = 352; Z = 2.

T / K	293(2)	240(2)	180(2)	120(2)	83(2)
CCDC number	1940102	1940103	1940104	1940105	1940106
λ / Å, radiation	0.71073, MoKα				
Crystal color, habit	Colorless, block				
Crystal size / mm³	0.28×0.40× 0.48	0.27×033× 0.34	0.27×033× 0.34	0.27×033× 0.34	0.27×033× 0.34
Crystal system	Triclinic				
Space group	$P\bar{1}$				
a / Å	8.661(2)	8.604(2)	8.549(2)	8.507(3)	8.511(3)
b / Å	9.7587(15)	9.718(2)	9.7014(13)	9.6857(13)	9.6752(16)
c / Å	12.379(2)	12.330(3)	12.2995(16)	12.2513(17)	12.207(2)
α / °	98.602(7)	98.854(7)	99.073(6)	99.278(7)	99.373(8)
β / °	90.91(2)	90.760(13)	90.765(12)	90.929(18)	91.212(19)
γ / °	107.622(7)	107.483(16)	107.392(18)	107.37(2)	107.48(3)
Volume / Å^3	983.9(3)	969.6(4)	959.3(3)	948.6(4)	943.4(4)
ρ_{calc} / g·cm^{-3}	1.101	1.117	1.130	1.142	1.149
μ / mm^{-1}	0.061	0.062	0.062	0.063	0.063
θ range / °	2.473 – 26.022	2.487 – 26.020	2.502 – 26.021	2.810 – 26.022	2.516 – 26.011
Reflections collected	14499	16739	16620	16349	16202
Unique reflections	3876	3811	3773	3726	3710
Parameters / restraints	232 / 0	232 / 0	232 / 0	307 / 0	307 / 0
GooF on F^2	1.027	1.047	1.028	1.029	1.057
R_1 [I>2σ(I)]	0.0525	0.0549	0.0513	0.0465	0.0448
wR^2 (all data)	0.1519	0.1542	0.1377	0.1247	0.1149
Max. / min. residual electron density / e·Å^{-3}	0.215 / –0.147	0.211 / –0.163	0.225 / –0.178	0.213 / –0.202	0.268 / –0.209

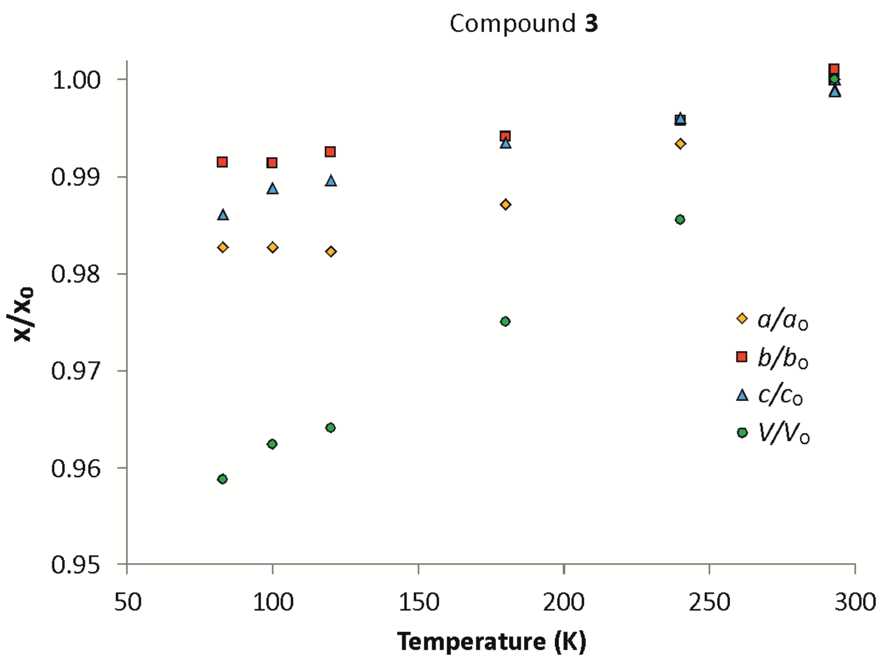

Figure S54. Temperature dependence of the normalized unit cell lengths and volume of compound **3**.

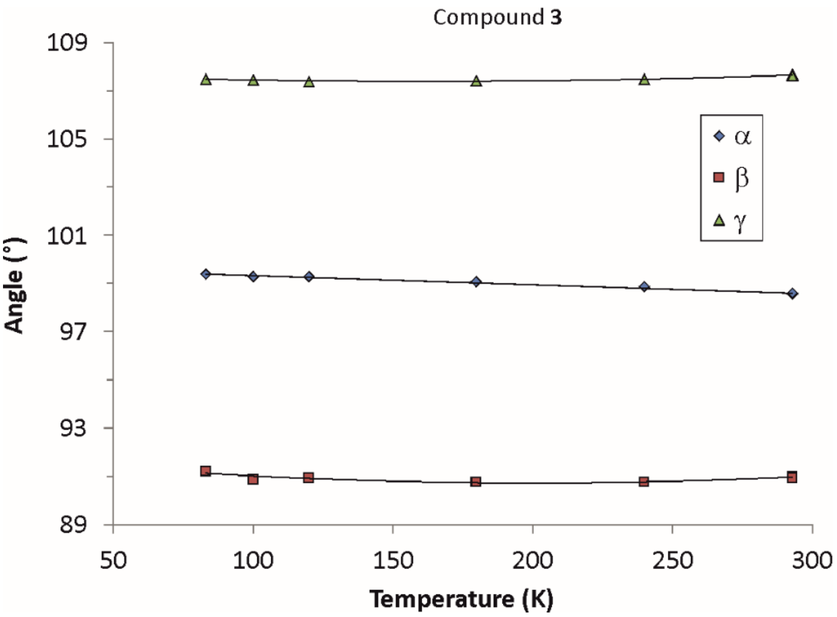

Figure S55. Temperature dependence of the unit cell angles (°) of compound **3**.

Table S12. Temperature dependence of **s**elected bond lengths (Å) and angles (°) of compound **3.**

**T (K)**	**293**	**240**	**180**	**120**	**100**	**83**
Sum ∠ CBC	360.0(2)	360.0(2)	360.0(2)	360.0(1)	360.0(1)	360.0(1)
B–C	1.577(3)<br>1.579(3)<br>1.587(3)	1.574(3)<br>1.583(3)<br>1.585(3)	1.576(3)<br>1.581(3)<br>1.590(3)	1.579(2)<br>1.584(2)<br>1.589(2)	1.5795(19)<br>1.5826(18)<br>1.5858(18)	1.579(2)<br>1.584(2)<br>1.588(2)
∠ BC3 – aryl	53.68(9)<br>53.06(8)<br>52.74(6)	53.66(9)<br>53.07(8)<br>53.15(6)	53.57(8)<br>52.91(7)<br>53.69(5)	53.46(7)<br>52.89(7)<br>53.91(5)	53.52(7)<br>52.91(7)<br>53.92(6)	53.30(7)<br>53.01(6)<br>53.86(5)

Table S13. Temperature dependence of the properties of crystals of compound **3**: Volume within van der Waals (V_m), Hirshfeld (V_H), and surface of the crystal voids (V_v), crystal packing coefficient (c_k), solvent accessible volume (V_{solv}), and percentage of intermolecular contacts. pfu…per formula unit.

**T (K)**	**293**	**240**	**180**	**120**	**100**	**83**
V_m / Å³	309.49	309.73	311.79	312.64	312.20	312.92
V_H / Å³	485.16	478.07	472.87	467.55	466.68	464.92
V_v / Å³	140.13	126.45	115.58	104.17	103.82	98.90
V_v / Å³ pfu	70.07	63.23	57.79	52.09	51.91	49.45
c_k	0.629	0.639	0.650	0.659	0.659	0.663
V_{solv} / Å³	11.0	10.9	9.1	9.0	9.0	9.0
C···C / %	0.2	0.2	0.2	0.3	0.2	0.2
C···H / %	17.0	17.6	18.1	18.6	18.5	18.8
H···H / %	82.8	82.2	81.7	81.1	81.3	81.0

Table S14. Temperature dependence of the intermolecular C–H···C and C···C interaction distances (Å) and angles (°) in compound **3**.

**T (K)**	**293**	**240**	**180**	**120**	**100**	**83**
H7···C19	2.924(2)	2.881(2)	2.860(2)	2.80(2)	2.841(2)	2.815(2)
C7···C19	3.816(4)	3.787(3)	3.779(3)	3.768(3)	3.765(2)	3.771(2)
∠(C7-H7-C19)	161.3(2)	162.0(2)	163.1(1)	162(1)	164.41(9)	162(1)
H14···C21	2.971(2)	2.929(2)	2.897(2)	2.86(2)	2.871(2)	2.85(2)
C14···C21	3.897(3)	3.865(3)	3.842(3)	3.823(3)	3.815(2)	3.815(2)
∠(C14-H14-C21)	174.1(2)	173.7(2)	172.9(1)	169(1)	171.96(9)	170(1)
H5···C12	3.028(3)	2.982(3)	2.934(2)	2.85(2)	2.897(2)	2.88(2)
C5···C12	3.943(4)	3.907(4)	3.870(3)	3.831(3)	3.832(3)	3.817(3)
∠(C5-H5-C12	168.4(2)	168.5(2)	168.5(1)	170(1)	168.18(9)	167(1)
C6···C6	3.421(4)	3.376(3)	3.343(3)	3.324(2)	3.319(2)	3.318(2)
C6···C23(methyl)	3.488(3)	3.460(3)	3.430(2)	3.402(2)	3.392(2)	3.376(2)

Table S15. Temperature dependence of the aryl···aryl (π···π) distances (Å) and angles (°) in crystals of **3**: nearest-neighbour (nn) C···C distances, centroid-centroid distances, interplanar separations, shifts, and slip angles. Aryl rings are numbered 1, 2, or 3 according to the C1, C2, or C3 atom being present and bonding to the boron atom, respectively.

**T (K)**	**Aryl···Aryl**	**nn C···C**	**Centroid-centroid distance**	**Interplanar separation**	**Shift**	**Slip angle**
**293**	#1···#1	3.421(5)	5.3311(19)	2.996(4)	4.409(3)	55.8
	#2···#2	3.633(6)	4.9301(19)	3.523(4)	3.449(4)	44.4
**240**	#1···#1	3.375(4)	5.2606(19)	2.995(4)	4.325(3)	55.3
	#2···#2	3.587(5)	4.9088(19)	3.479(4)	3.463(4)	44.9
**180**	#1···#1	3.343(4)	5.2075(16)	2.991(4)	4.263(3)	54.9
	#2···#2	3.544(4)	4.8973(16)	3.440(4)	3.486(3)	45.4
**120**	#1···#1	3.3239(7)	5.1722(6)	2.9827(9)	4.2255(10)	54.8
	#2···#2	3.4938(8)	4.8779(5)	3.3963(9)	3.5013(10)	45.9
**100**	#1···#1	3.319(3)	5.1671(17)	2.980(3)	4.221(3)	54.8
	#2···#2	3.495(3)	4.8718(16)	3.397(3)	3.493(3)	45.8
**83**	#1···#1	3.318(3)	5.1715(14)	2.973(3)	4.231(2)	54.9
	#2···#2	3.469(3)	4.8633(14)	3.375(3)	3.502(3)	46.1

VI. ^{1}H, $^{13}C\{^{1}H\}$, ^{11}B NMR spectra, GC-MS and HRMS

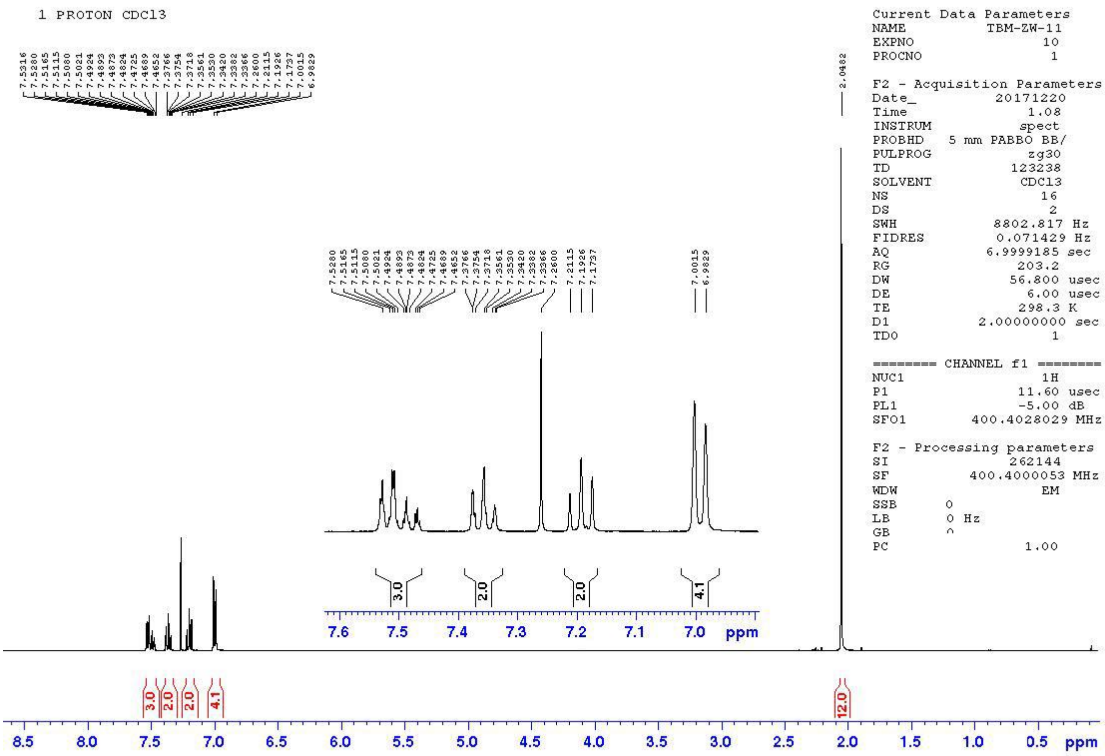

Figure S56. ^{1}H NMR spectrum (400 MHz, $CDCl_3$) of **1**.

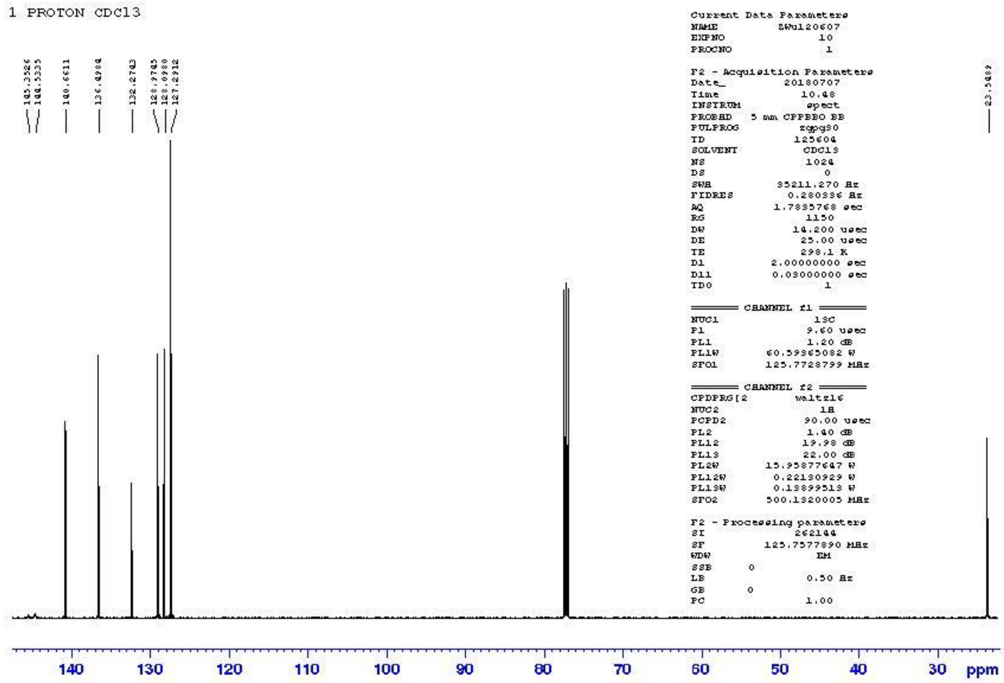

Figure S57. $^{13}C\{^{1}H\}$ NMR spectrum (126 MHz, $CDCl_3$) of **1**.

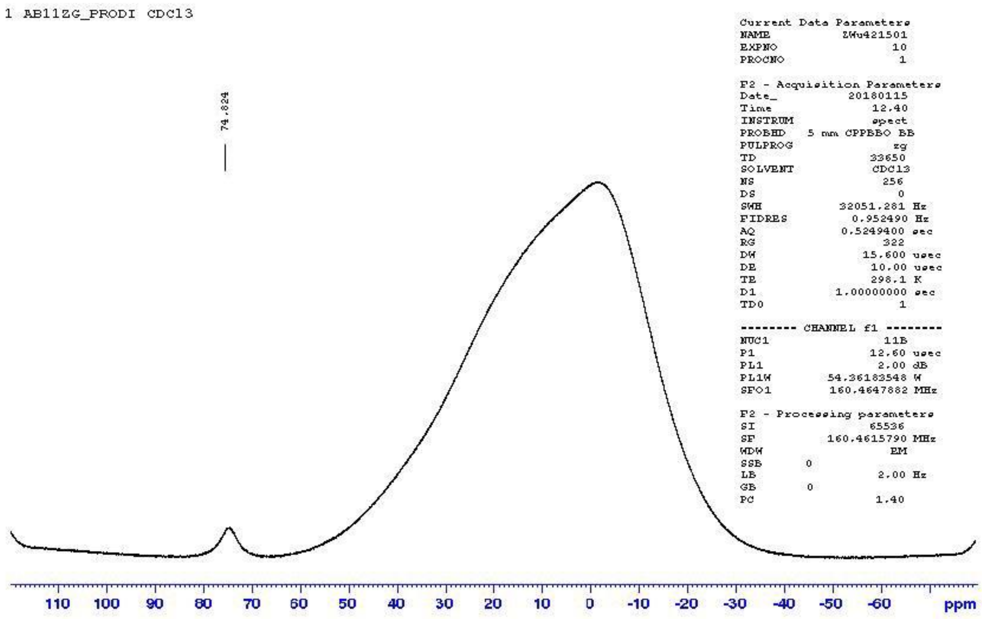

Figure S58. ^{11}B NMR spectrum (160 MHz, $CDCl_3$) of **1**.

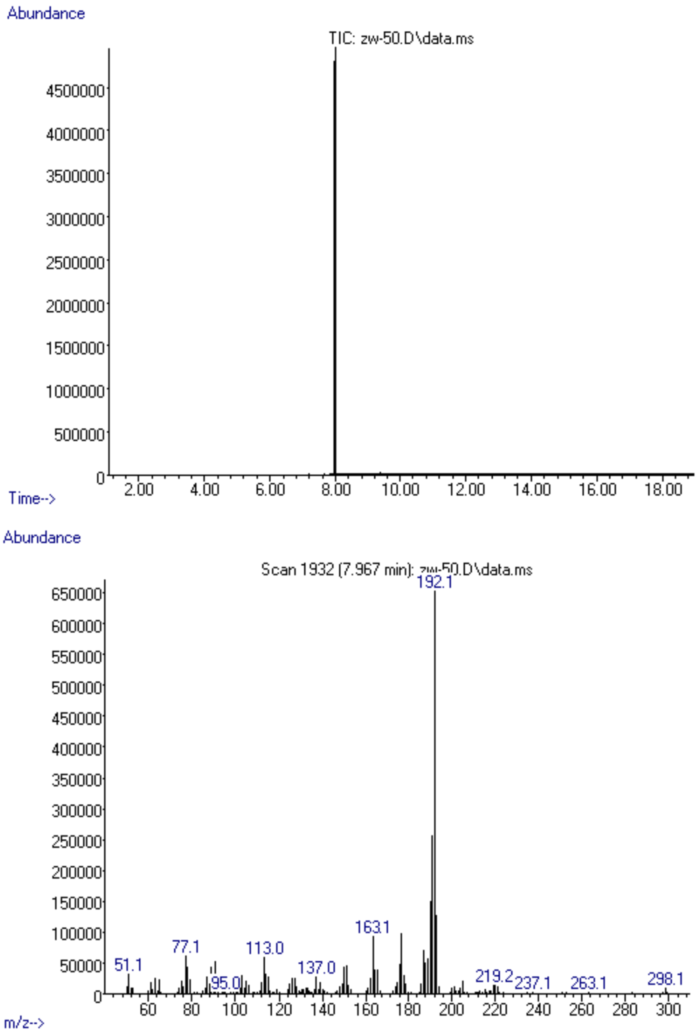

Figure S59. GC-MS total ion chromatogram (TIC) and MS $(EI)_{m/z}$ of **1**.

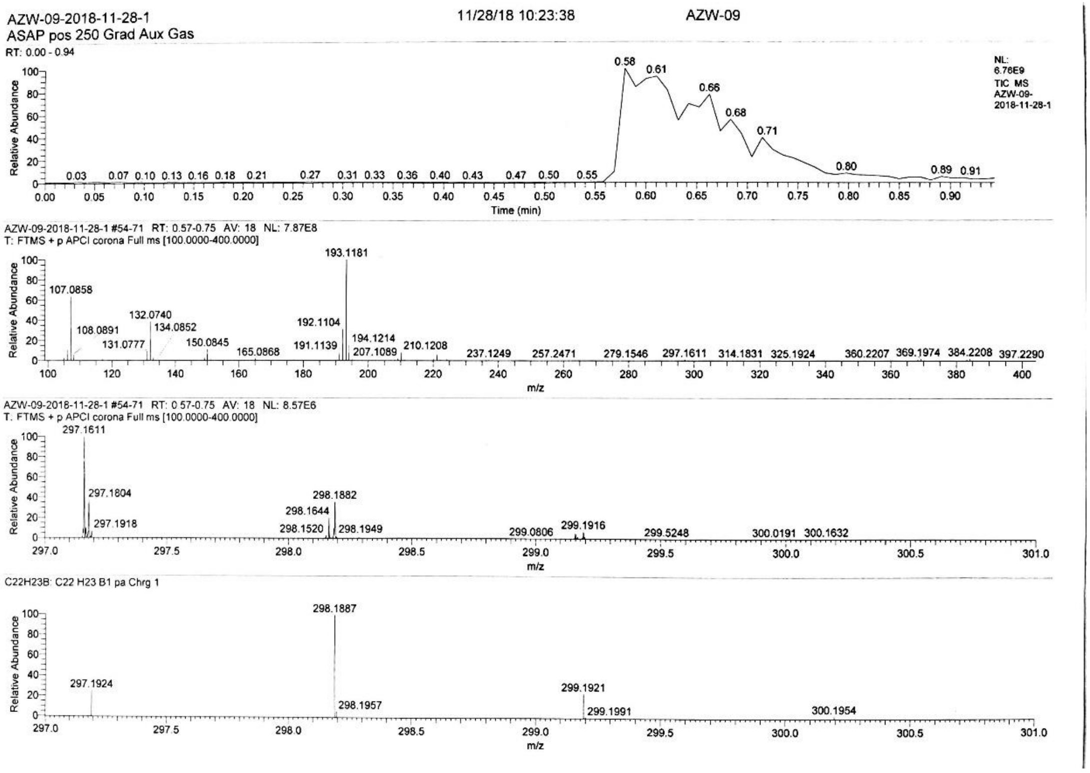

Figure S60. ESI-HRMS of **1**.

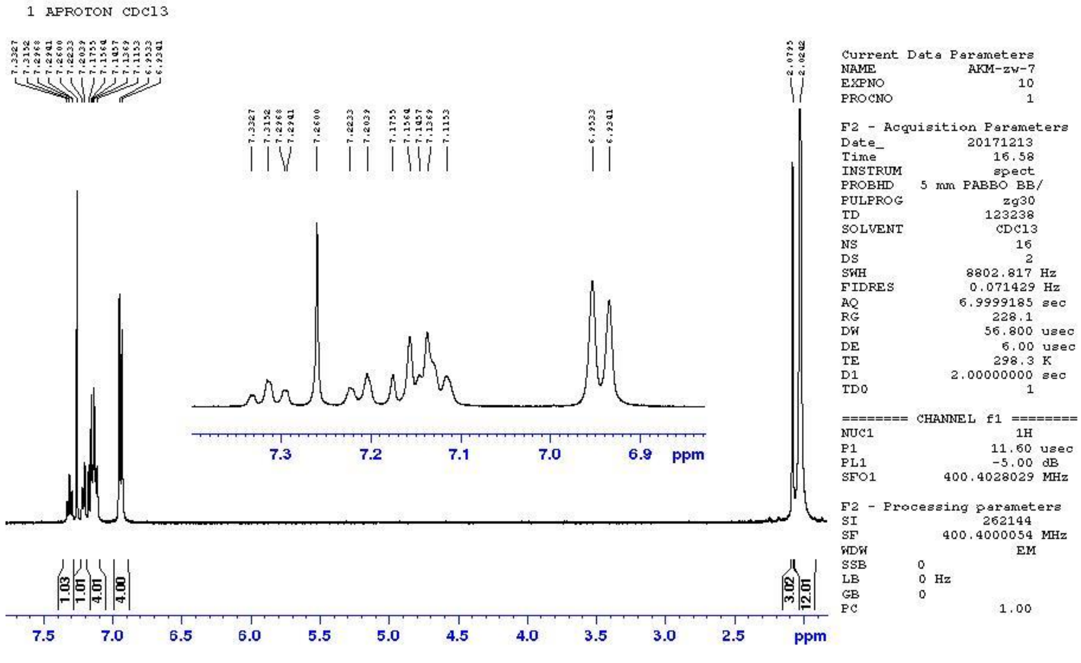

Figure S61. ^{1}H NMR spectrum (400 MHz, $CDCl_3$) of **2**.

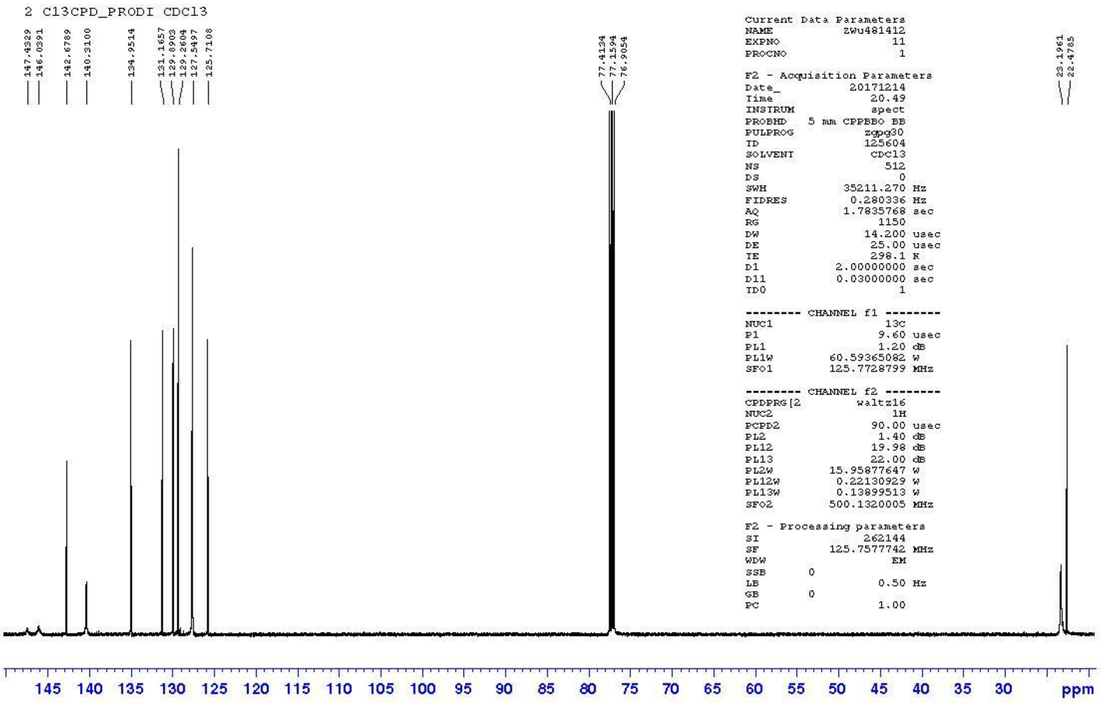

Figure S62. $^{13}C\{^1H\}$ NMR spectrum (126 MHz, $CDCl_3$) of **2**.

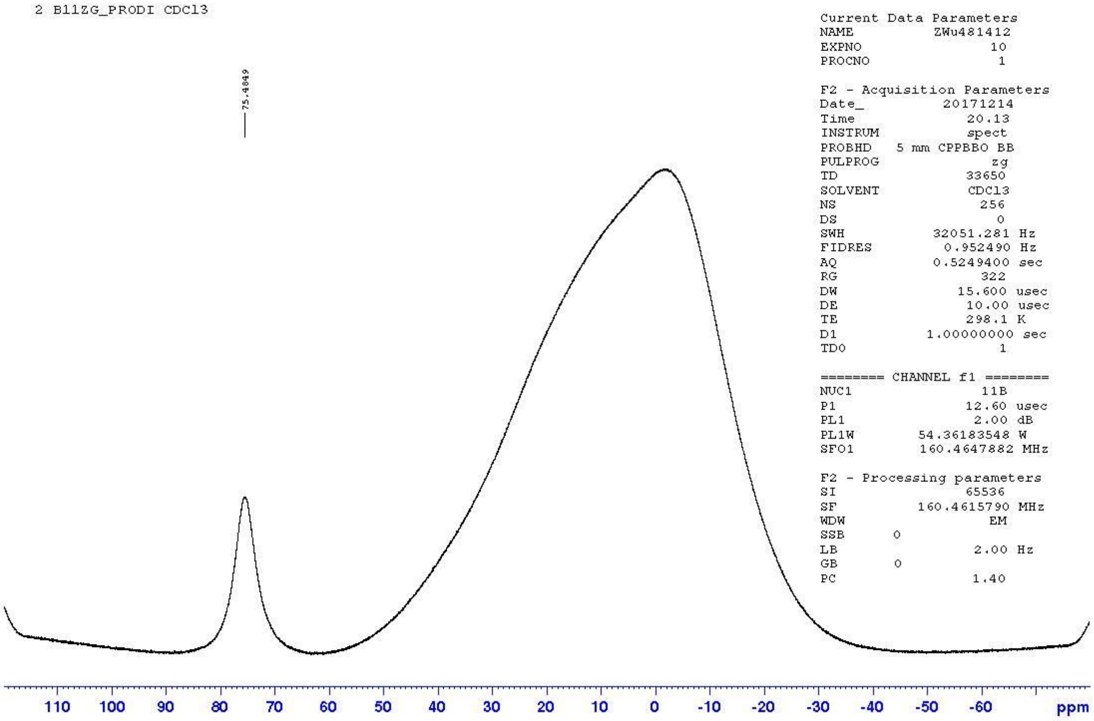

Figure S63. ^{11}B NMR spectrum (160 MHz, $CDCl_3$) of **2**.

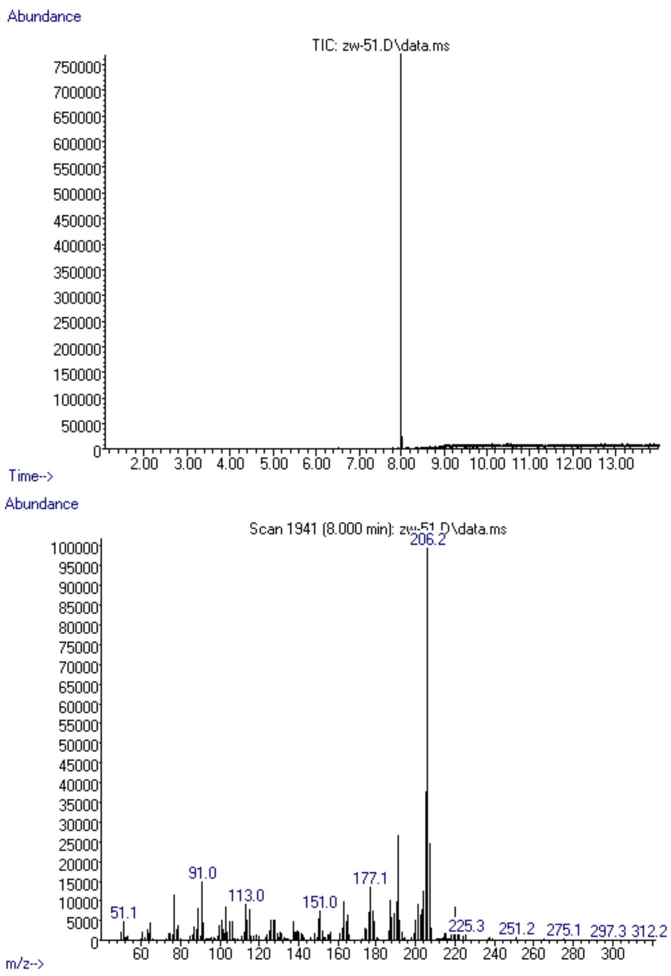

Figure S64. GC-MS total ion chromatogram (TIC) and MS $(EI)_{m/z}$ of **2**.

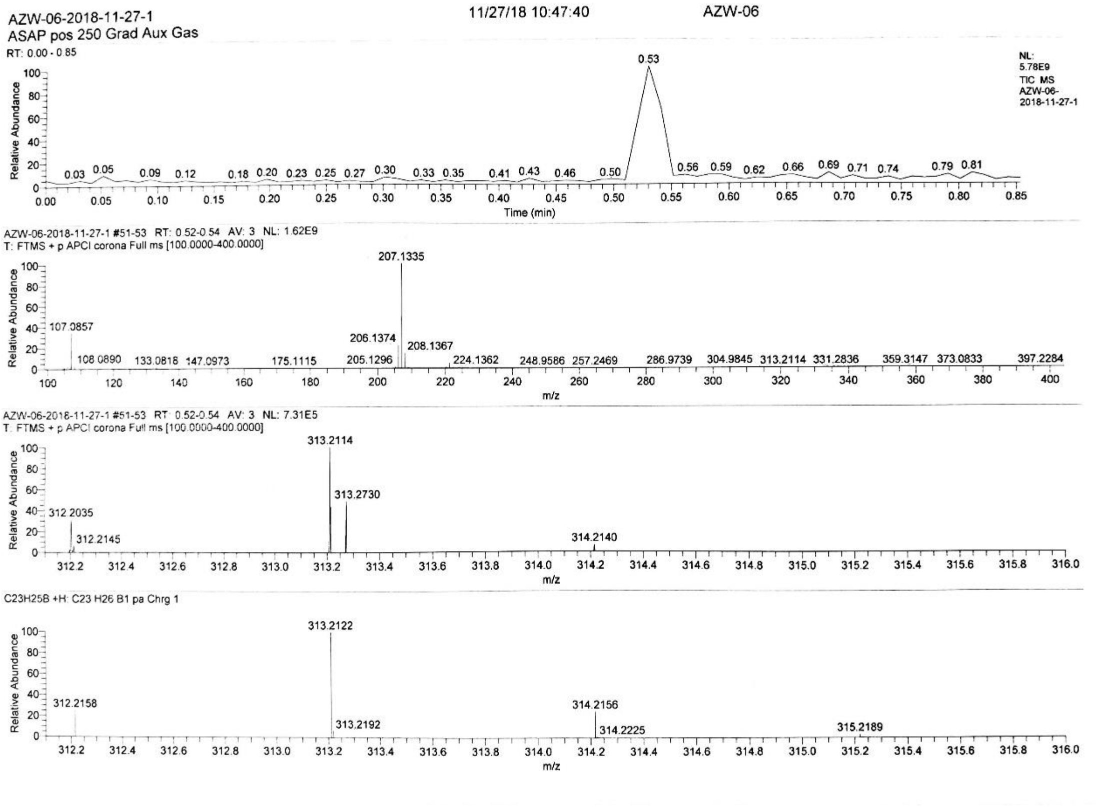

Figure S65. ESI-HRMS of **2**.

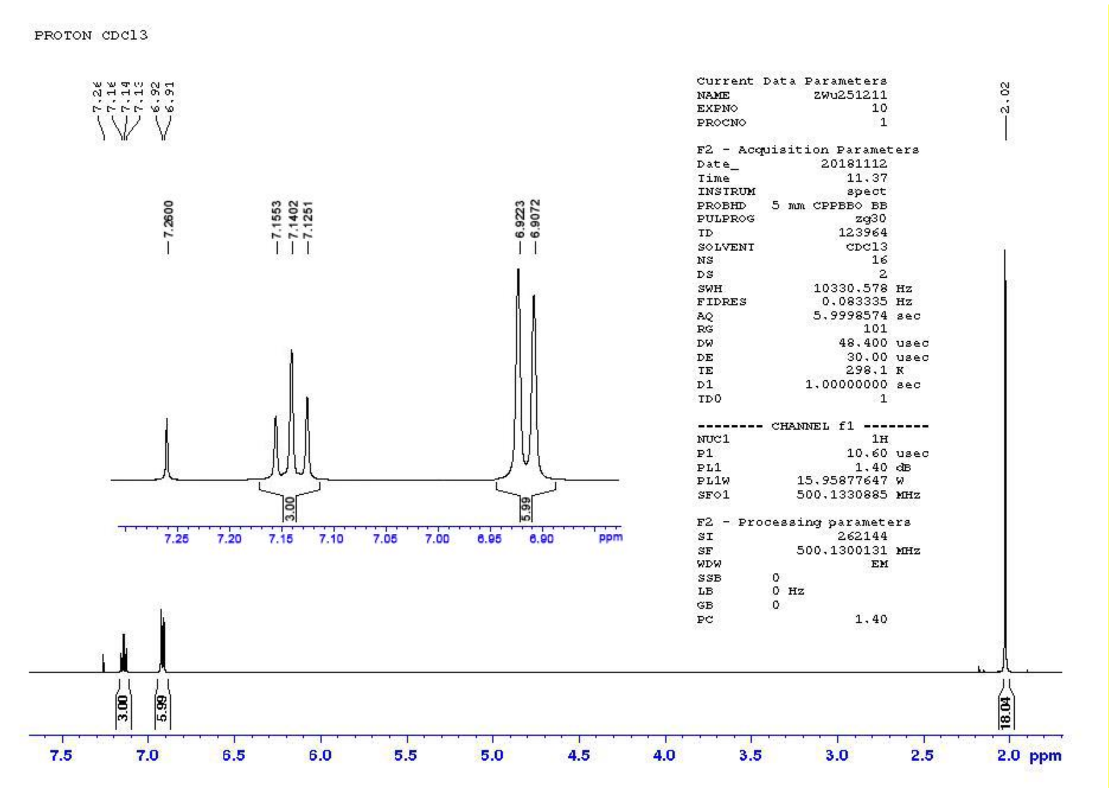

Figure S66. ^{1}H NMR spectrum (500 MHz, $CDCl_3$) of **3**.

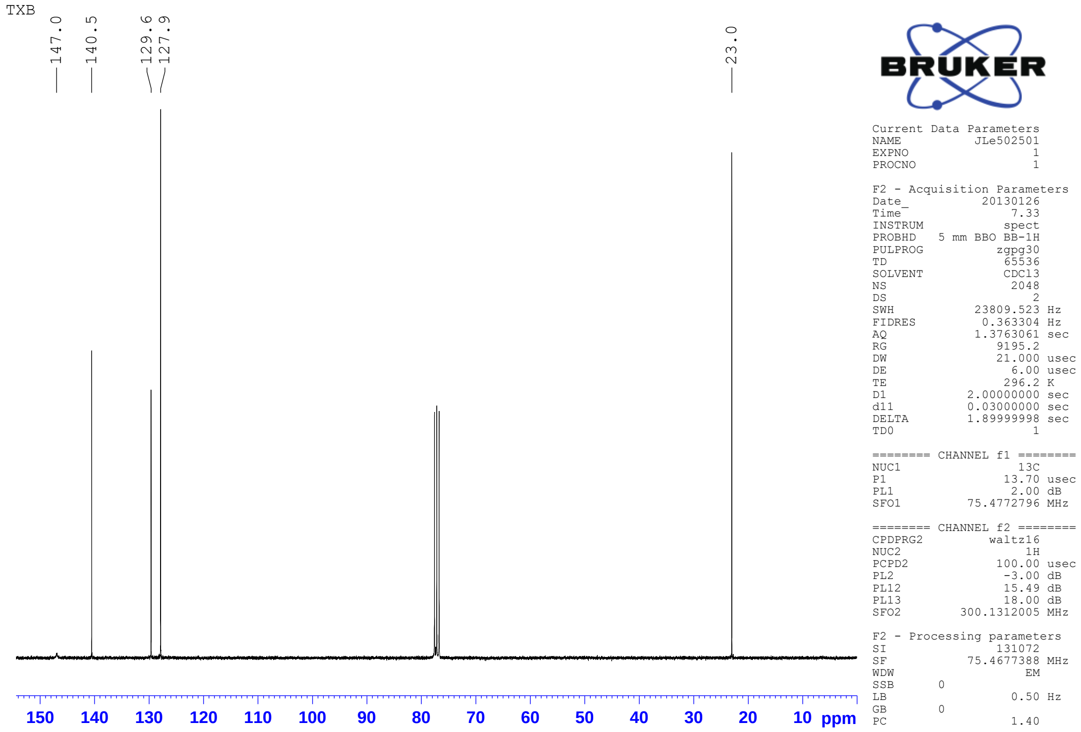

Figure S67. $^{13}C\{^{1}H\}$ NMR spectrum (126 MHz, $CDCl_3$) of **3**.

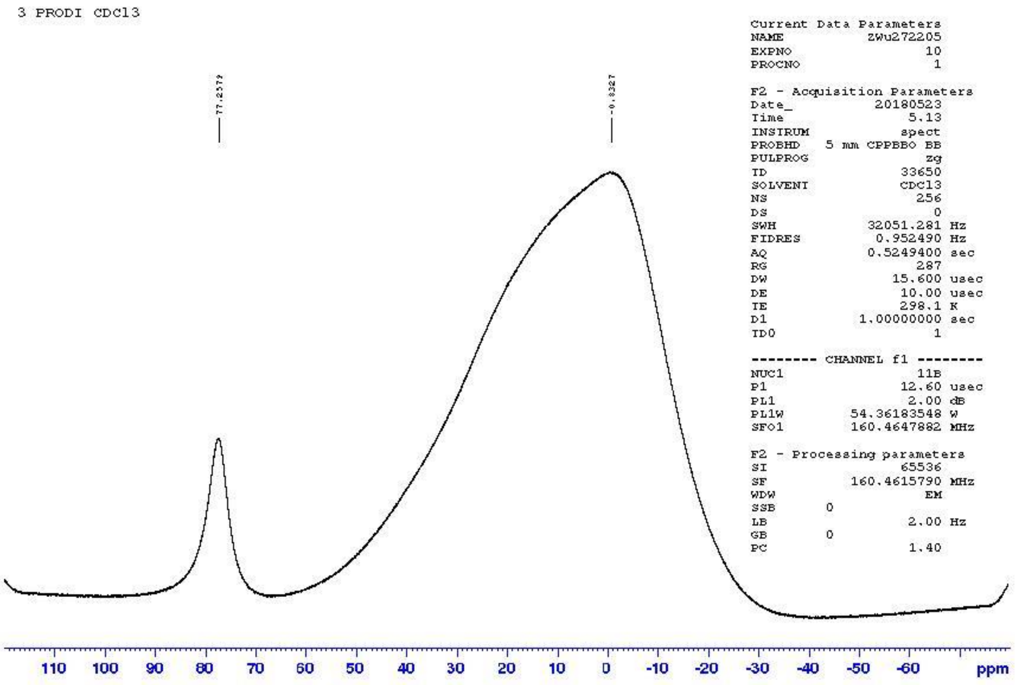

Figure S68. ^{11}B NMR spectrum (160 MHz, $CDCl_3$) of **3**.

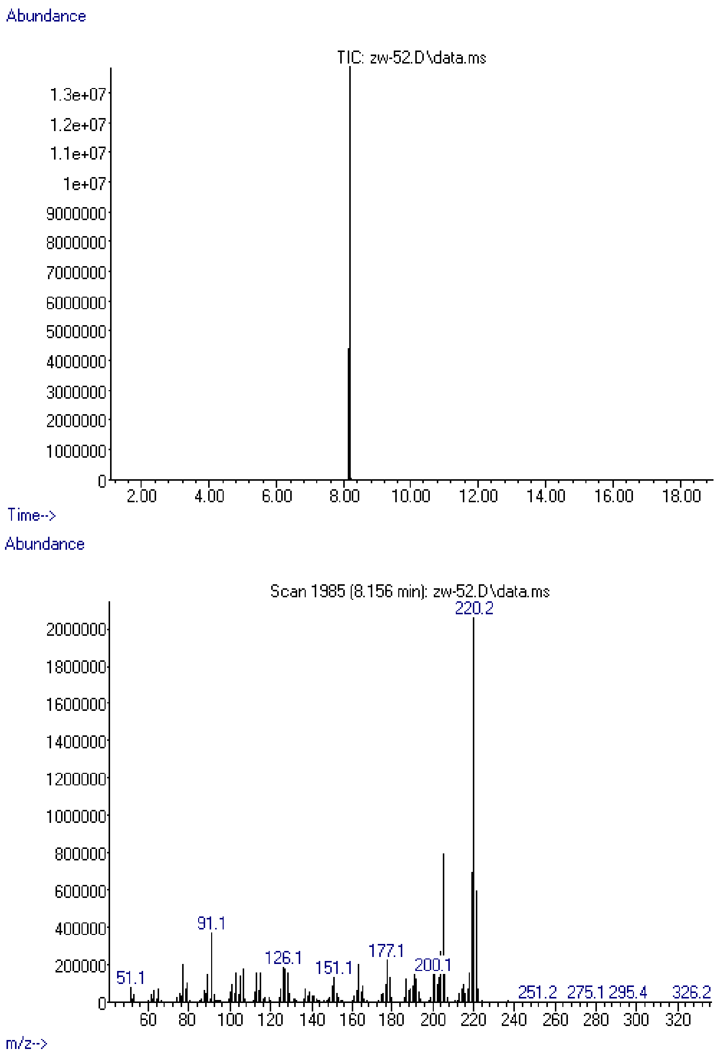

Figure S69. GC-MS total ion chromatogram (TIC) and MS $(EI)_{m/z}$ of **3**.

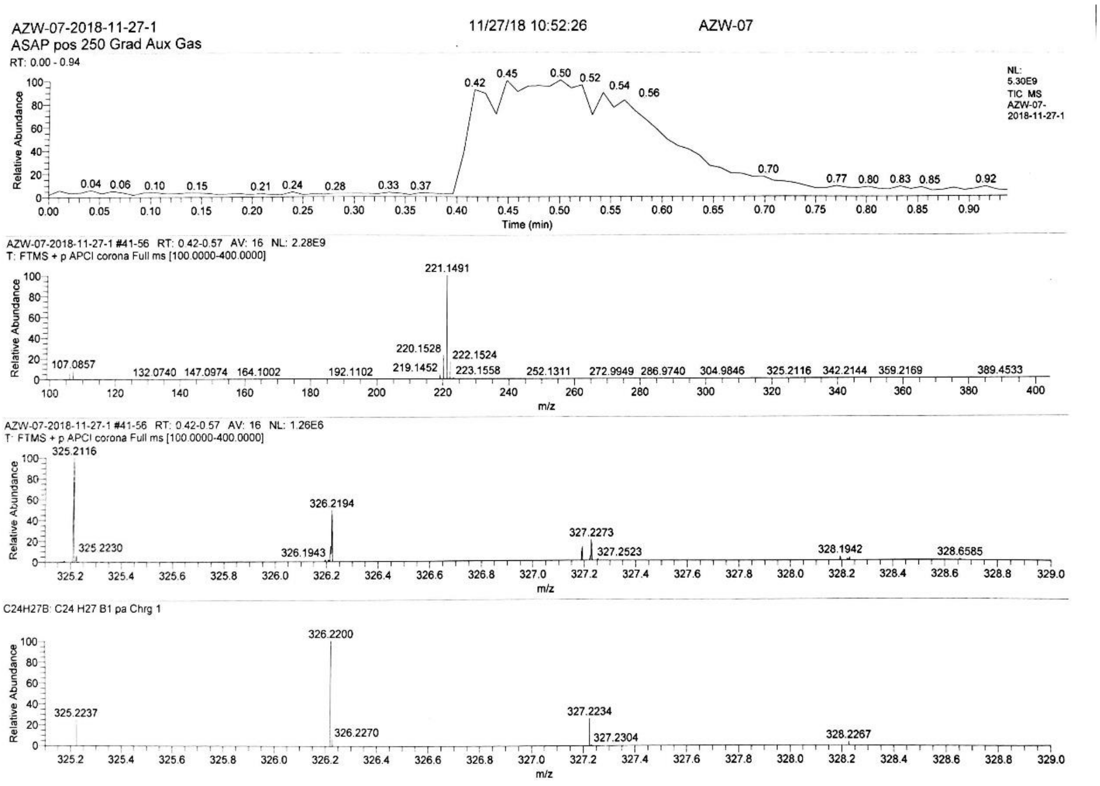

Figure S70. ESI-HRMS of **3**.

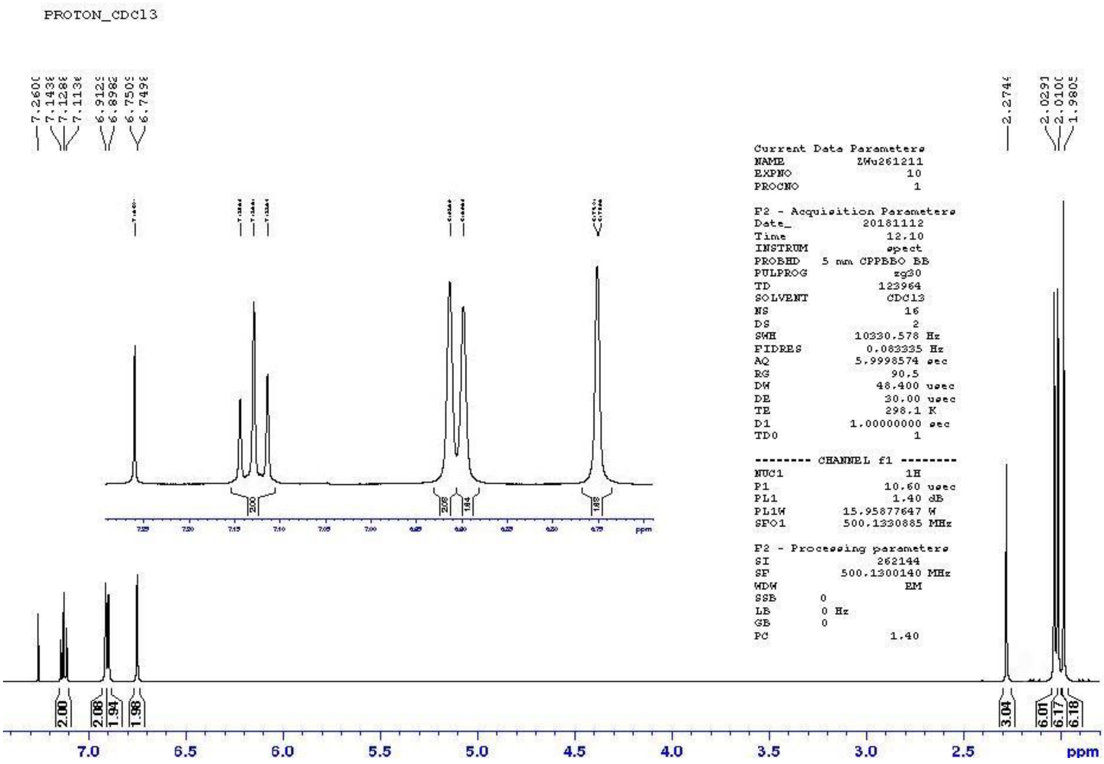

Figure S71. ^{1}H NMR spectrum (500 MHz, $CDCl_3$) of **4**.

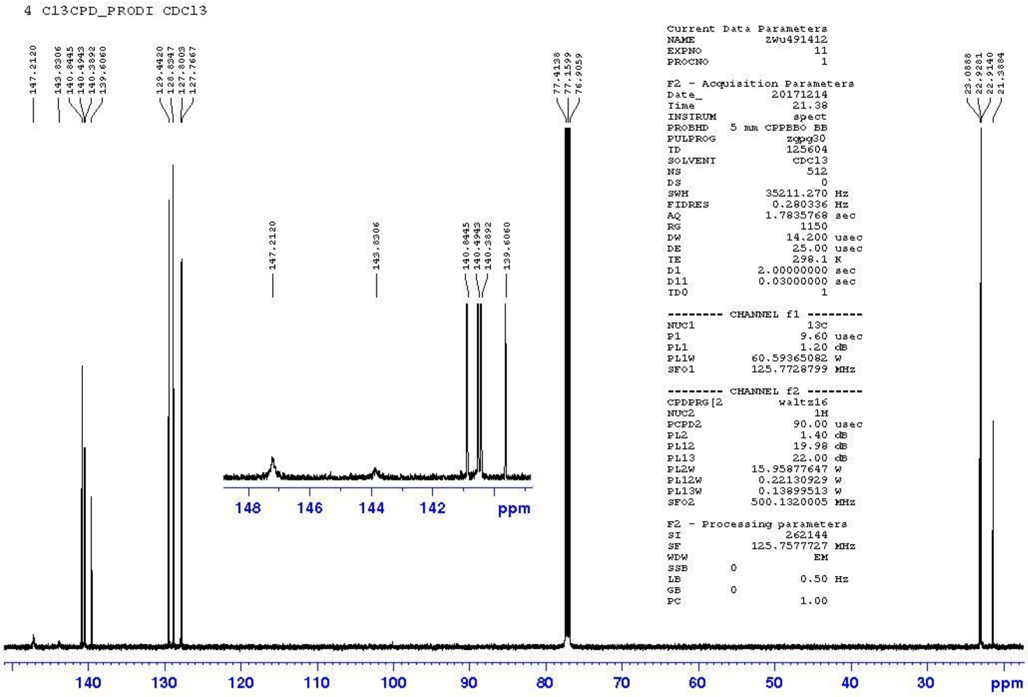

Figure S72. $^{13}C\{^1H\}$ NMR spectrum (126 MHz, $CDCl_3$) of **4**.

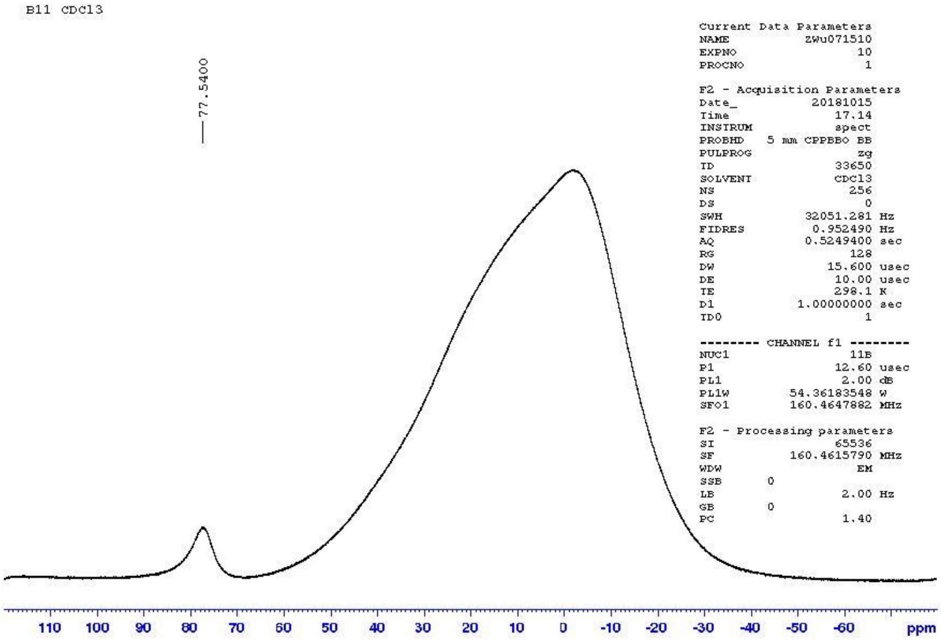

Figure S73. ^{11}B NMR spectrum (160 MHz, $CDCl_3$) of **4**.

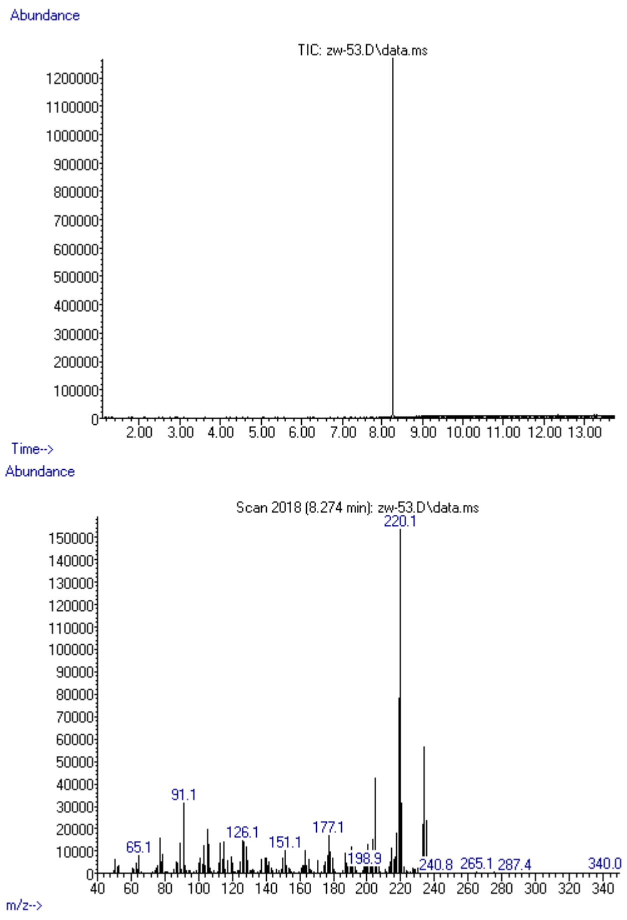

Figure S74. GC-MS total ion chromatogram (TIC) and MS $(EI)_{m/z}$ of **4**.

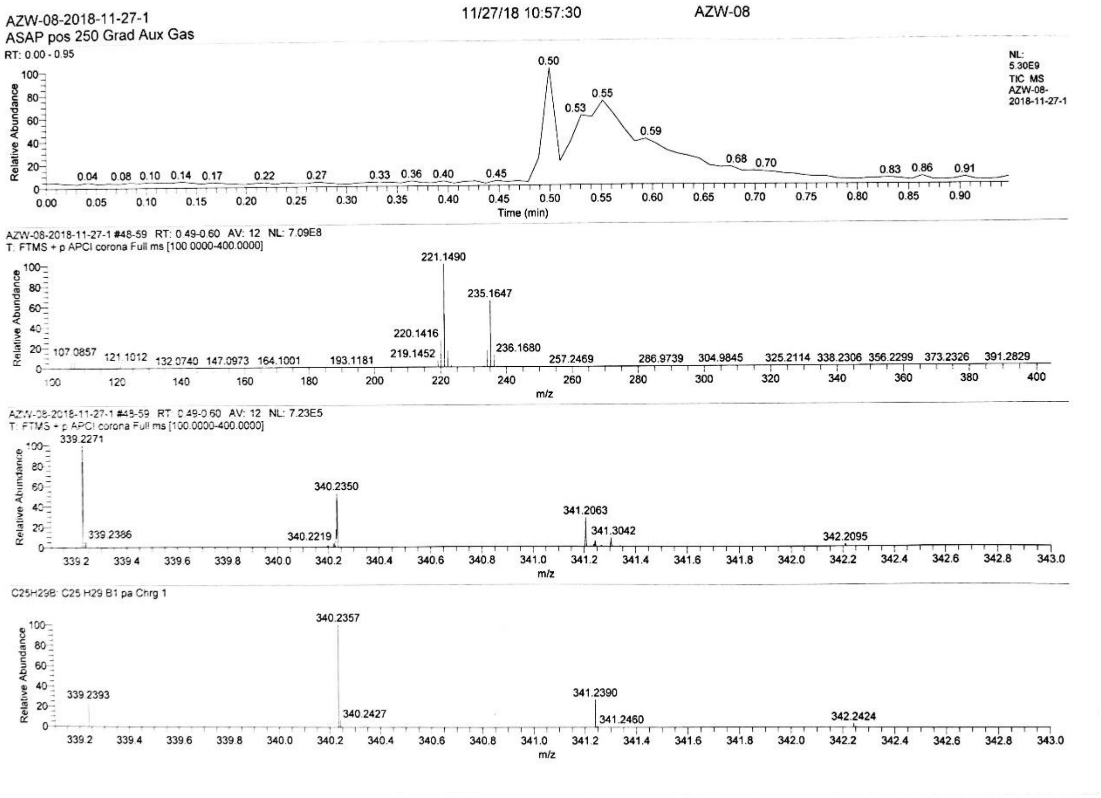

Figure S75. ESI-HRMS of **4**.

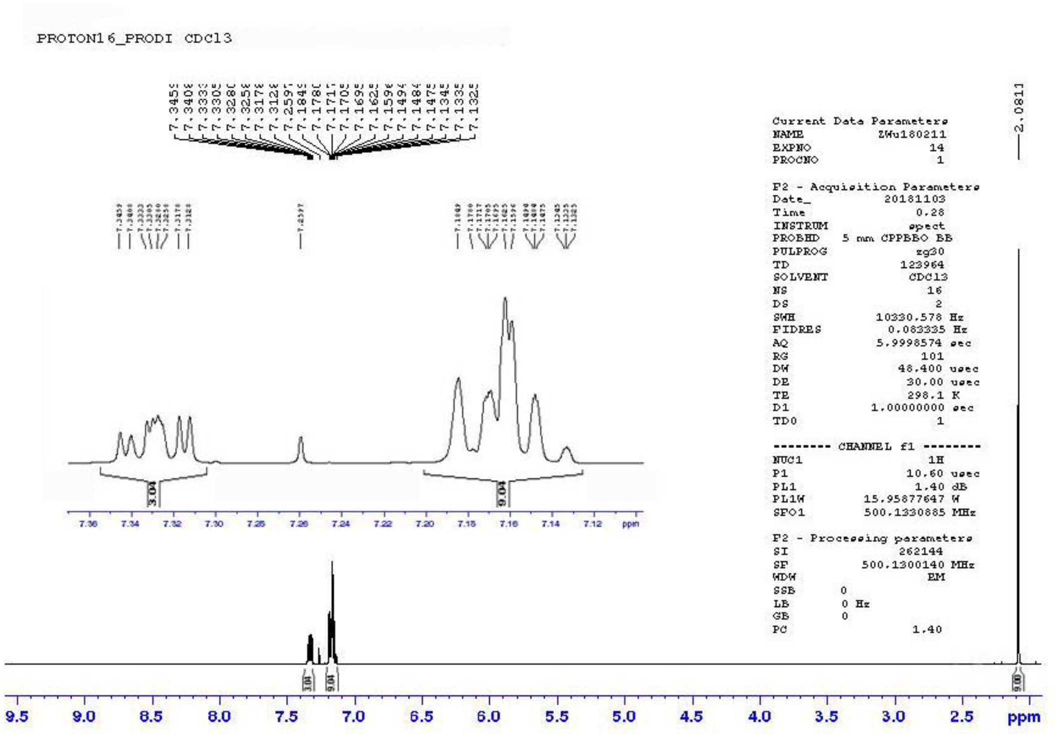

Figure S76. ^{1}H NMR spectrum (500 MHz, $CDCl_3$) of **tris(2-methylphenyl)borane**.

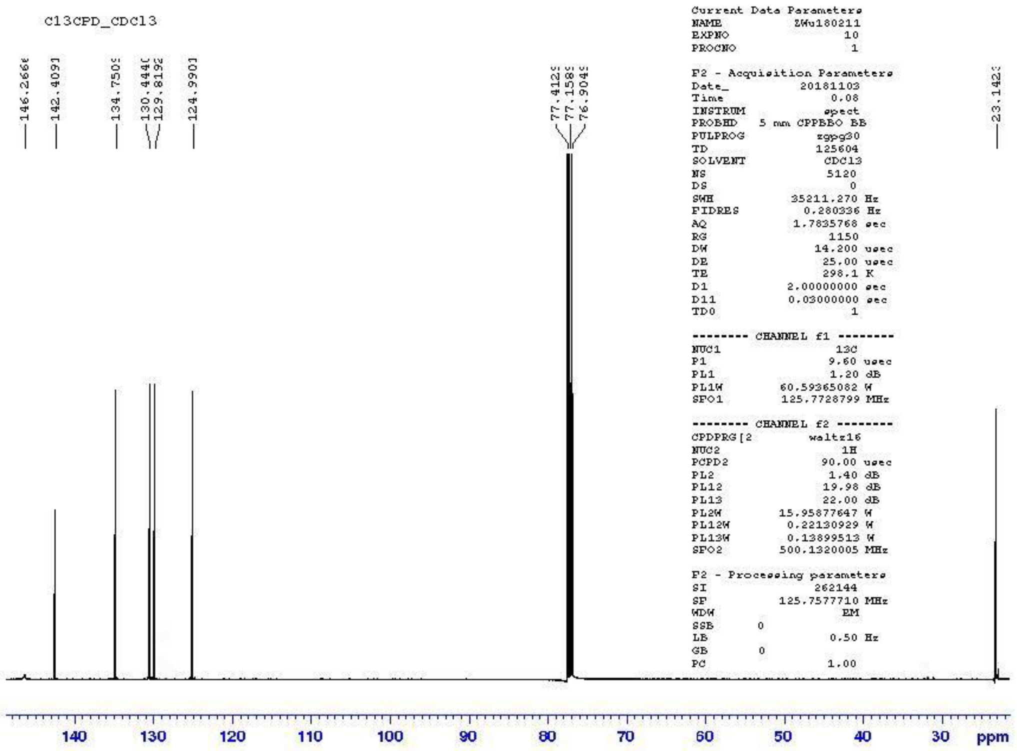

Figure S77. $^{13}C\{^{1}H\}$ NMR spectrum (126 MHz, $CDCl_3$) of **tris(2-methylphenyl)borane**.

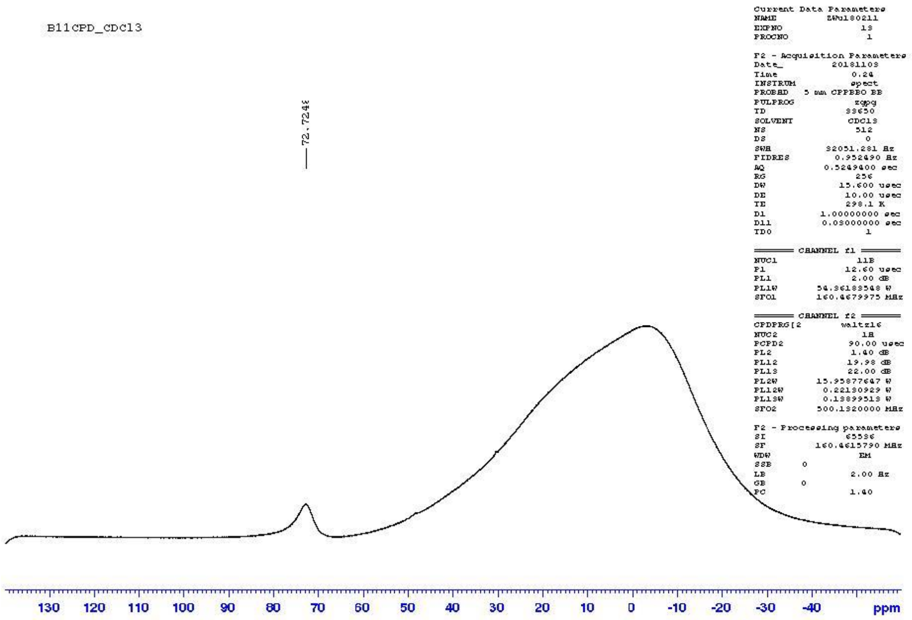

Figure S78. ^{11}B NMR spectrum (160 MHz, $CDCl_3$) of **tris(2-methylphenyl)borane**.

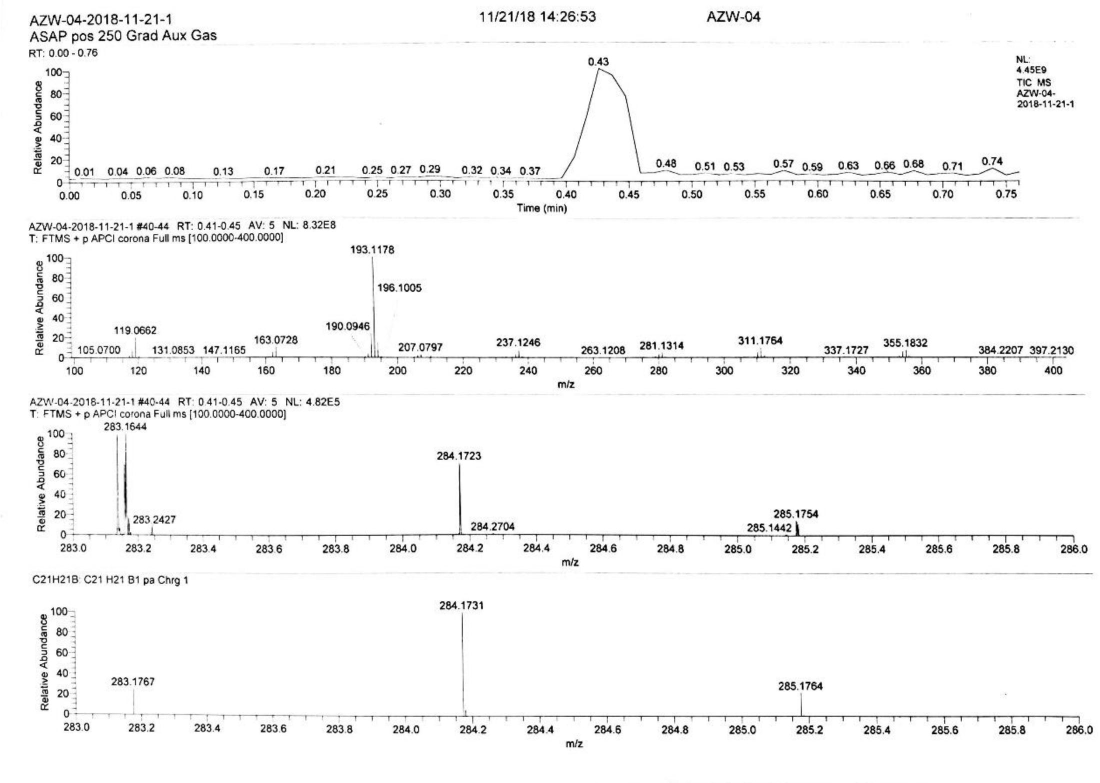

Figure S79. ESI-HRMS of **tris(2-methylphenyl)borane**.

VII. References

[1] H. Pan, G. L. Fu, Y. H. Zhao, C. H. Zhao, *Org. Lett.* **2011**, *13*, 4830-4833.

[2] B. H. Toby, *J. Appl. Crystallogr.* **2005**, *38*, 1040-1041.

[3] A. C. Larson, R. B. Von Dreele, Los Alamos National Laboratory Report LAUR, **2004**, pp. 86-748.

[4] G. M. Sheldrick, *Acta. Crystallogr. A Found. Adv.* **2015**, *71*, 3-8.

[5] G. M. Sheldrick, *Acta. Crystallogr. A Found. Adv.* **2008**, *64*, 112-122.

[6] C. B. Hübschle, G. M. Sheldrick, B. Dittrich, *J. Appl. Crystallogr.* **2011**, *44*, 1281-1284.

[7] K. D. Brandenburg, Crystal and Molecular Structure Visualization, Crystal Impact H. Putz & K. Brandenburg GbR, Bonn (Germany), **2017**.

[8] C. F. Macrae, I. J. Bruno, J. A. Chisholm, P. R. Edgington, P. McCabe, E. Pidcock, L. R. Monge, R. Taylor, J. van de Streek, P. A. Wood, *J. Appl. Crystallogr.* **2008**, *41*, 466-470.

[9] O. V. Dolomanov, L. J. Bourhis, R. J. Gildea, J. A. K. Howard, H. Puschmann, *J. Appl. Crystallogr.* **2009**, *42*, 339-341.

[10] M. J. Turner, J. J. McKinnon, S. K. Wolff, D. J. Grimwood, P. R. Spackman, D. Jayatilaka, M. A. Spackman, CrystalExplorer17 (2017), University of Western Australia, http://hirshfeldsurface.net.

[11] I. R. Morgan, A. E. J. Broomsgrove, P. Fitzpatrick, D. Vidovic, A. L. Thompson, I. A. Fallis, S. Aldridge, *Organometallics* **2010**, *29*, 4762-4765.

[12] TURBOMOLE V7.0, a development of University of Karlsruhe and Forschungszentrum Karlsruhe GmbH, 1989-2007; TURBOMOLE GmbH, since 2007; available from **http://www.turbomole.com**.

[13] M. von Arnim, R. Ahlrichs, *J. Comput. Chem.* **1998**, *19*, 1746-1757.

[14] a) A. D. Becke, *J. Chem. Phys.* **1993**, *98,* 5648-5652; b) C. Lee, W. Yang, R. G. Parr, *Phys. Rev. B: Condens. Matter Mater. Phys.* **1988**, *37*, 785-789; c) P. J. Stephens, F. J. Devlin, C. F. Chabalowski, M. J. Frisch, *J. Phys. Chem. A* **1994**, *98,* 11623-11627.

[15] G. Scalmani, M. J. Frisch, B. Mennucci, J. Tomasi, R. Cammi, V. Barone, *J. Chem. Phys.* **2006**, *124*, 94107.

[16] A. Schäfer, H. Horn, R. J. Ahlrichs, *J. Chem. Phys.* **1992**, *97*, 2571-2577.

[17] a) I. Lyskov, M. Kleinschmidt, C. M. Marian, *J. Chem. Phys.* **2016**, *144*, 034104; b) C. M. Marian, A. Heil, M. Kleinschmidt, *WIREs Comput. Mol. Sci.* **2019**, *9*, e1394.
[18] A. D. Becke, *J. Chem. Phys.* **2014**, *140*, 1372.
[19] a) M. Kleinschmidt, C. M. Marian, *Chem. Phys.* **2005**, *311*, 71-79; b) M. Kleinschmidt, J. Tatchen, C. M. Marian, *J. Comput. Chem.* **2002**, *23*, 824-833; c) M. Kleinschmidt, J. Tatchen, C. M. Marian, *J. Chem. Phys.* **2006**, *124*, 124101.
[20] J. Neugebauer, M. Reiher, C. Kind, B. A. Hess, *J. Comput. Chem.* **2002**, *23*, 895-910.
[21] a) M. Etinski, J. Tatchen, C. M. Marian, *J. Chem. Phys.* **2011**, *134*, 154105; b) M. Etinski, J. Tatchen, C. M. Marian, *Phys. Chem. Chem. Phys.* **2014**, *16*, 4740-4751.
[22] T. J. Penfold, E. Gindensperger, C. Daniel, C. M. Marian, *Chem. Rev.* **2018**, *118*, 6975-7025.
[23] a) J. J. McKinnon, A. S. Mitchell, M. A. Spackman, *Chem. Eur. J.* **1998**, *4*, 2136-2141; b) J. J. McKinnon, M. A. Spackman, A. S. Mitchell, *Acta Crystallogr B* **2004**, *60*, 627-668; c) M. A. Spackman, D. Jayatilaka, *CrystEngComm* **2009**, *11*, 19-32; d) M. A. Spackman, P. G. Byrom, *Chem. Phys. Lett.* **1997**, *267*, 215-220.
[24] M. J. Turner, J. J. McKinnon, D. Jayatilaka, M. A. Spackman, *CrystEngComm* **2011**, *13*, 1804-1813.
[25] a) J. J. McKinnon, D. Jayatilaka, M. A. Spackman, *Chem. Commun.* **2007**, *0*, 3814–3816; b) A. Parkin, G. Barr, W. Dong, C. J. Gilmore, D. Jayatilaka, J. J. McKinnon, M. A. Spackman, C. C. Wilson, *CrystEngComm* **2007**, *9*; c) M. A. Spackman, J. J. McKinnon, *CrystEngComm* **2002**, *4*, 378-392.
[26] a) T. Dahl, *Acta Chem. Scand.* **1994**, *48*, 95-106; b) C. A. Hunter, J. K. M. Sanders, *J. Am. Chem. Soc.* **1990**, *112*, 5525-5534; c) M. O. Sinnokrot, C. D. Sherrill, *J. Phys. Chem. A* **2004**, *108*, 10200-10207; d) M. O. Sinnokrot, E. F. Valeev, C. D. Sherrill, *J. Am. Chem. Soc.* **2002**, *124*, 10887-10893; e) L. Loots, L. J. Barbour, *John Wiley & Sons*, Chichester, UK, **2012**, 109-124.
